# Uncovering personalized glucose responses and circadian rhythms from multiple wearable biosensors with Bayesian dynamical modeling

**DOI:** 10.1016/j.crmeth.2023.100545

**Published:** 2023-07-31

**Authors:** Nicholas E. Phillips, Tinh-Hai Collet, Felix Naef

**Affiliations:** 1 Institute of Bioengineering, School of Life Sciences, Ecole Polytechnique Fédérale de Lausanne (EPFL), 1015 Lausanne, Switzerland; 2 Nutrition Unit, Service of Endocrinology, Diabetology, Nutrition and Therapeutic Education, Department of Medicine, Geneva University Hospitals (HUG), 1211 Geneva, Switzerland; 3 Diabetes Centre, Faculty of Medicine, University of Geneva, 1211 Geneva, Switzerland

**Keywords:** wearables, biosensors, continuous glucose monitor (CGM), circadian rhythms, Bayesian inference, Kalman filter

## Abstract

Wearable biosensors and smartphone applications can measure physiological variables over multiple days in free-living conditions. We measure food and drink ingestion, glucose dynamics, physical activity, heart rate (HR), and heart rate variability (HRV) in 25 healthy participants over 14 days. We develop a Bayesian inference framework to learn personal parameters that quantify circadian rhythms and physiological responses to external stressors. Modeling the effects of ingestion events on glucose levels reveals that slower glucose decay kinetics elicit larger postprandial glucose spikes, and we uncover a circadian baseline rhythm for glucose with high amplitudes in some individuals. Physical activity and circadian rhythms explain as much as 40%–65% of the HR variance, whereas the variance explained for HRV is more heterogeneous across individuals. A more complex model incorporating activity, HR, and HRV explains up to 15% of additional glucose variability, highlighting the relevance of integrating multiple biosensors to better predict glucose dynamics.

## Highlights

• Simultaneous monitoring of food intake, glucose levels, and physical and heart activity
• Glucose dynamics modeled with meal responses plus a 24-h rhythmic baseline
• Model parameters learned on a personalized level with exact Bayesian inference
• Addition of physical and heart activity helps better explain glucose variability

## Motivation

Wearable biosensors measure physiological variables with high temporal resolution over multiple days and are increasingly employed in clinical settings, such as continuous glucose monitoring in diabetes care. Such datasets bring new opportunities and challenges, and patients, clinicians, and researchers are today faced with a common challenge: how to best summarize and capture relevant information from multimodal wearable time series? Here, we aim to provide insights into individual glucose dynamics and their relationships with food and drink ingestion, time of day, and coupling with other physiological states such as physical and heart activity. To this end, we generate and analyze multiple wearable-device data through the lens of a parsimonious mathematical model with interpretable components and parameters. A key innovation of our method is that the models are learned on a personalized level for each participant within a Bayesian framework, which enables the characterization of interindividual heterogeneity in features such as the glucose response time following meals or underlying circadian baseline rhythm. This framework may prove useful in populations at risk for cardiometabolic diseases.

## Graphical abstract

**Figure:**
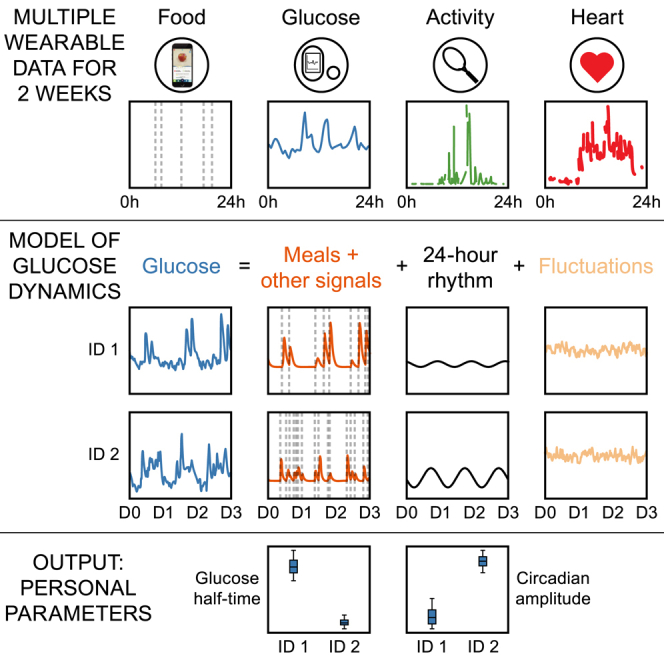

## Teaser

Phillips et al. simultaneously measure consumed food and drinks, continuous glucose levels, and physical and heart activity and use dynamical modeling to integrate these data streams. The method uses Bayesian inference to learn personalized parameters related to the dynamical responses to external stressors and 24-h rhythms of the wearable signals.

## Introduction

Wearable biosensors and smartphone applications are increasingly used to measure multiple physiological variables, including glucose levels, food consumption, and physical and heart activity. In contrast to traditional lab measurements taken at a single time point, the high-resolution wearable time series data record dynamic changes of physiological variables in response to external perturbations and as a function of the time of day. While these wearable data have the potential to provide a dynamic view of health states,^1^ a major challenge in both clinical and research settings is how to extract physiologically relevant information from wearable time series data, and, in particular, when multiple data modalities are combined.

Glucose regulation is a prime example of a dynamic and complex physiological system, as the body is confronted with irregular inputs (i.e., food intake, especially of carbohydrates) and controlled glucose uptake by organs (e.g., muscles, liver). As such, glycemic regulation employs a range of homeostatic mechanisms, including the glucose-insulin negative feedback loop, whereby insulin secretion by the pancreas is tightly regulated to avoid both low (hypoglycemic) and high (hyperglycemic) levels of glucose.^2^^,^^3^ Understanding glucose regulation is important for human health, as long-term chronic hyperglycemia in diabetes can lead to micro- and macrovascular complications,^4^ and glucose levels show a non-linear association with vascular diseases even in populations without diabetes.^5^^,^^6^^,^^7^

As glucose homeostasis is inherently dynamic and glucose levels fluctuate throughout the day, continuous glucose monitors (CGMs) have gained popularity due to their high temporal resolution. CGMs measure glucose in interstitial fluid continuously for up to 10–14 days with satisfactory clinical accuracy compared with reference capillary blood glucose values.^8^^,^^9^ Standardized CGM-derived metrics such as the coefficient of variation (CV) and the time in range (the fraction of time spent within the desired range of 3.9–10.0 mmol/L, or 70–180 mg/dL) have been adopted in clinical practice to assess glycemic control in diabetes with insulin treatment.^10^^,^^11^^,^^12^^,^^13^ At a more fine-grained level, CGMs have been combined with smartphone records of ingestion events to predict postprandial (postmeal) glycemic responses (PPGRs), where the PPGR is often defined as the area under the glucose curve for the 2 h following a recorded ingestion event.^14^^,^^15^^,^^16^

Nonetheless, neither the standardized CGM metrics nor the PPGR approach provides a complete picture of the entire glucose time series and its fluctuations over the 24-h clock. Physiological processes in humans, including glucose metabolism, follow circadian rhythms,^17^^,^^18^^,^^19^^,^^20^ and responses to oral glucose tests are more pronounced in the evening than in the morning.^21^ A pre-breakfast rise in glucose levels, termed the “dawn phenomenon,” has been observed since the early 1980s and is often linked with a concomitant early morning rise of cortisol,^22^^,^^23^ but the amplitude and phase of circadian rhythms in baseline glucose levels have thus far not been well described at an individual level. Identifying the relative contribution of the circadian rhythms to the glucose time series would be particularly helpful for the interpretation of 24-h CGM reports, which are often discussed with patients to identify patterns of low and high glucose values and to guide treatment.^24^

In addition to glucose, other physiological responses are accessible with biosensors, such as heart rate (HR; beats per minute) and heart rate variability (HRV), where HRV is typically quantified with metrics such as the root-mean-square of successive differences (RMSSD) between heart beats.^25^ Epidemiological data have linked low HRV with high glucose levels,^26^^,^^27^ and a reduction in HRV has been shown to predict the development of autonomic neuropathy before symptom onset among diabetic patients.^28^ The simultaneous measurement of HR and HRV can provide insights into the autonomic nervous system activity,^29^ as HR receives inputs from both the sympathetic nervous system (SNS; the “flight or fight'' response) and parasympathetic nervous system (PNS; the “rest and digest” response), while the HRV-derived RMSSD metric is dominated by the PNS via vagal nerve activity.^30^ Both HR and HRV are modulated by physical activity, which can now also be conveniently measured with a triaxial accelerometer. Furthermore, it is known that glucose levels are affected during exercise, which motivates attempts to connect physical activity from wearable device signals to continuous glucose data.^31^^,^^32^^,^^33^

Regarding the analysis of wearable data streams, a diverse range of glucose models have been proposed over the last decades,^34^^,^^35^^,^^36^ ranging from minimal models^37^ to more detailed simulators with dozens of parameters^38^ and neural networks.^39^^,^^40^ Recent efforts have also attempted to utilize additional multimodal wearable signals to either improve glucose forecasting or provide more accessible proxies for glucose without using CGMs.^33^^,^^41^^,^^42^^,^^43^ Many of these methods are specialized toward short-range forecasting, which is certainly useful in applications like the artificial pancreas.^44^ In a different context, researchers and clinicians need new wearable data analysis tools to perform statistical comparisons between individuals and quantify changes in glucose regulation across multiple time points and different disease states, but such approaches to extract personalized summary metrics from the global recordings remain comparatively unexplored.

In this study, we acquired multiple wearable biosensor data to monitor food and drink ingestion, glucose excursions, physical activity, HR, and HRV in individuals in free-living conditions. Our aim was to quantify how external perturbations (such as ingestion events and physical activity) and baseline circadian rhythms determine temporal glucose levels on a personalized level. To this end, we develop data-driven computational models to analyze data streams from multiple wearables with distinct model components that capture the interactions between physiological variables, 24-h rhythms, and random fluctuations. Individual-specific parameters are learned in a Bayesian framework providing parameter uncertainties and enabling statistical comparisons between participants. We subdivide the problem of analyzing the multiple signals by creating three successive mathematical models that include different subsets of variables. Our three-tiered modeling reveals the high degree of personalization across a wide range of metrics, even within a healthy population, from glucose decay kinetics, circadian rhythms in baseline glucose levels, and the dependence between HR and HRV. Future studies will be able to re-use the framework to describe personalized longitudinal changes over time in response to interventions and to cardiometabolic diseases.

## Results

### Measuring multivariable physiological time series in free-living conditions

To quantify the personalized dynamics of individuals in free-living conditions, we measured ingestion events, glucose levels, physical activity, HR, and HRV for 25 participants over a 2-week period. Participants (16 males, 9 females) were young (mean age 33.0 ± SD 11.0), had a normal weight (mean BMI 22.7 ± 2.8 kg/m^2^; one person with overweight and one person with obesity), and had a normal blood pressure (systolic 117.6 ± 11.4 mm Hg, diastolic 75.3 ± 7.9 mm Hg) (participant characteristics shown in Figure S1). Participant ID 14 was previously diagnosed with diabetes, but currently treated only with lifestyle measures (and not pharmacological treatment), and hypertension, treated with perindopril. Participants were asked to record all food and drink consumption and add a manual free text annotation of the content with the smartphone application myCircadianClock.^45^ Each ingestion event was automatically time stamped by the app. The adherence (defined as at least two meals separated by at least 5 h in a given day^46^) was above 83% for all participants (Table S1).

We measured glucose levels continuously using the Abbott FreeStyle Libre Pro CGM, which records interstitial glucose levels every 15 min over a 2-week period. As the device is blinded, participants were unable to access their glucose data during the study period, thus avoiding feedback on their eating behavior. Five participants wore two sensors (on different arms), with the aim of validating that parameters estimated from the model were consistent between the two sensors (noted ID A and B in the figures). Physical activity, HR, and HRV were measured for each participant over the 2-week study period using the CamNtech Actiheart version 5 device, and the physical and heart activity data were also blinded to participants during the study.

### Multiple wearable time series data reveal complex dynamical responses as a function of external inputs and time of day

**Figure 1:**
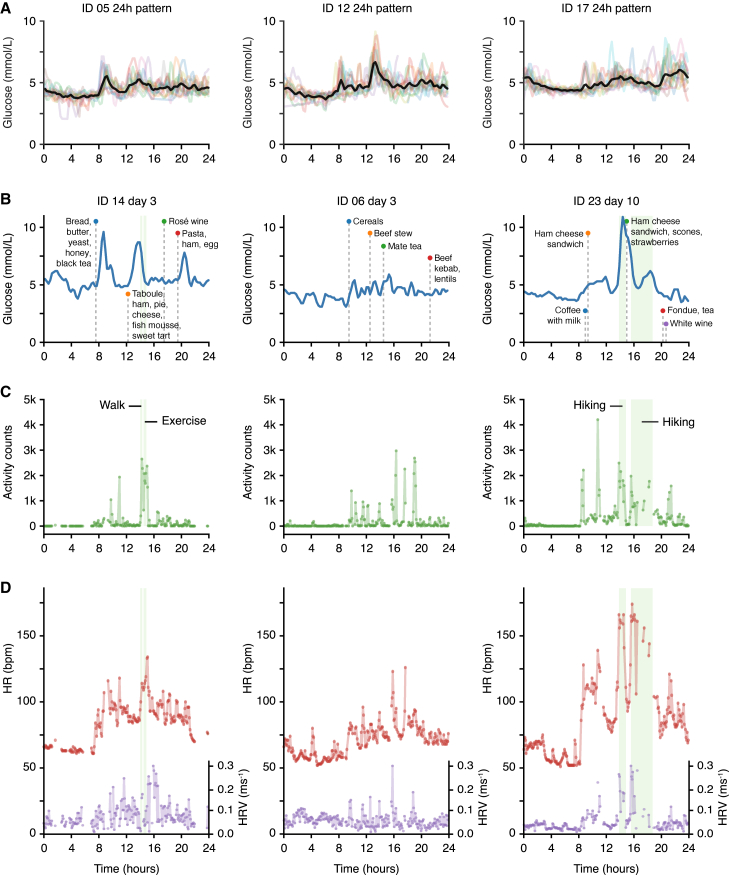
Exploratory analysis of wearable signals: examples of 24-h trends and responses to external stressors (A) Continuous glucose monitoring (CGM) data: superposition of all recorded days of data shown on the same 24-h scale for three different participants (see related Figure S7 for all participants). Black, average over all days; colored lines, data for individual days; time axis, wall clock time. (B) Selected day examples of CGM glucose levels alongside recorded ingestion events for three participants (same individuals shown in B–D). Blue, glucose levels; green shade, recorded activity events; time axis, wall clock time; vertical dashed lines, ingestion events. (C) Selected day examples of physical activity measured with the CamNtech Actiheart device. Green, physical activity; green shade, recorded activity events; time axis, clock time. (D) Selected day examples of HR and HRV measured with the CamNtech Actiheart device. Green shade, recorded activity events; purple, heart rate variability (HRV) (quantified with RMSSD^−1^ in ms^−1^); red, heart rate (HR) in beats per minute (bpm); time axis, wall clock time. See also Figures S2–S7 for visualization of all data for all participants.

Before developing a detailed mathematical model, we performed initial data exploration to identify the key features that we wanted to capture in the model (overview of all data streams shown in Figures S2–S6). First, we superposed the recorded days of glucose data based on time of day and found marked individual-specific mean 24-h patterns, with the highest mean glucose levels occurring at different times of the day depending on the individual (Figure 1A; all participants shown in Figure S7). These unique 24-h trends could be caused by either food or drink ingestion (i.e., external perturbations) and/or an underlying circadian baseline trend in glucose. This motivated the inclusion of both ingestion events and circadian rhythms in the model of glucose dynamics as separate components.

Further exploratory analysis of the multiple wearable signals showed rich interactions between subsets of the five measured variables (i.e., ingestion events, glucose, activity, HR, and HRV). As expected, glucose levels often rose following ingestion events, and for some of the individuals, recorded meals seemed to lead to large, predictable peaks in glucose (Figure 1B, IDs 14 and 23), while others showed a more complex relationship, with small postprandial glucose spikes that were barely larger than the glucose fluctuations between meals (Figure 1B, ID 06). Based on these observations, and compared with CGM analysis methods that focus exclusively on PPGRs for 2–3 h,^14^^,^^15^^,^^16^ our goal is now to dynamically model the entire glucose time series over 2 weeks, including the fluctuating glucose levels occurring overnight or during longer intervals between ingestion events.

Visual inspection of the physical and heart activity data showed that spikes in physical activity typically coincided with an increased HR and HRV (as measured with RMSSD^−1^) (Figures 1C and 1D). By creating a joint dynamical model of the three signals (physical activity, HR, and HRV), we aimed to uncover the interindividual heterogeneity in the coupling between the multiple signals as well as the underlying circadian rhythms.

Finally, we observed spikes in glucose levels following physical activity for some individuals (Figures 1B–1D, ID 23), which could be caused by the release of glucose under the influence of adrenaline/epinephrine or glucagon. However, to establish more firmly whether physical and heart activity signals can explain glucose variation, we develop below a dynamical model to mathematically assess the extent to which the total glucose signal across the 2-week study period is predictable by the combined meal, physical, HR, and HRV data.

### Slow glucose dynamics is associated with large postprandial glucose spikes

**Figure 2:**
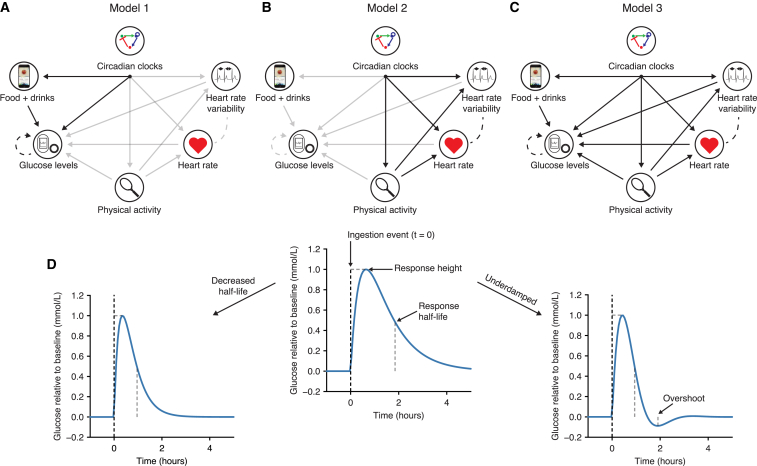
Schematic showing the three different models and parameter interpretation (A–C) The glucose and ingestion events interaction model (A, model 1), the physical and heart activity interaction model (B, model 2), and the full model (C, model 3). Solid arrows represent direct unidirectional influences, while dashed lines represent correlated fluctuations that are not specifically directional. (D) A meal or drink event causes a glucose increase to a specific meal height relative to the baseline glucose value. The response half-life determines how quickly glucose returns to baseline. Underdamping (defined as a negative damping coefficient) leads to an overshoot below the baseline values.

**Figure 3:**
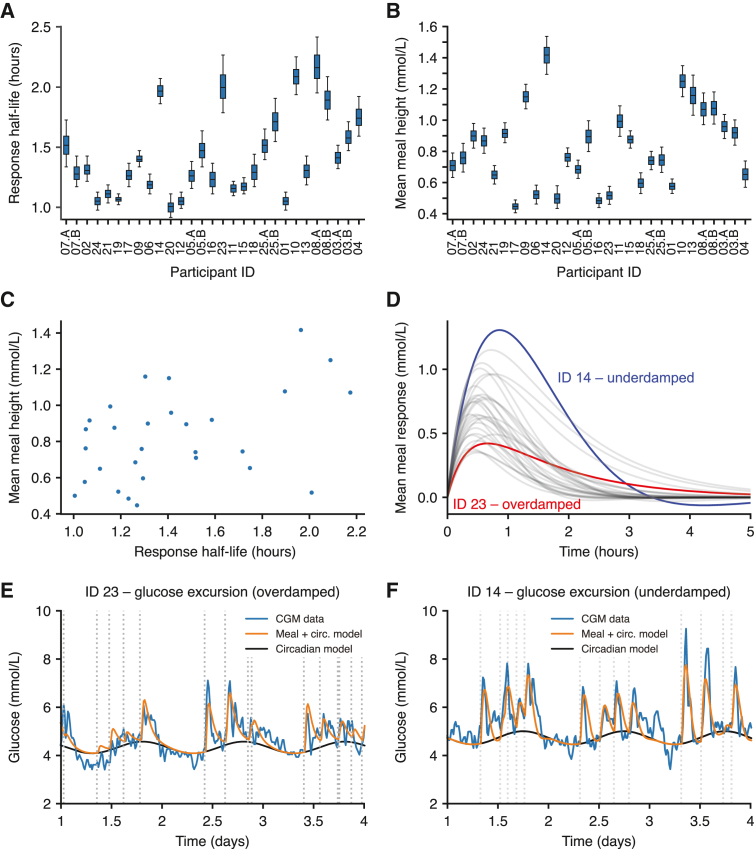
Characterizing participant-specific postmeal glycemic responses (A) The inferred glucose response half-life for each participant, defined as the model-predicted time it would take for glucose levels to fall to 0.5 mmol/L following a peak of 1 mmol/L. The boxplots represent the 25th, 50th (median), and 75th percentiles of the posterior distribution and the whiskers represent the 5th and 95th percentiles. (B) The average meal glucose spike height calculated as the mean height over all meals consumed during the experiment. (C) The average meal height as a function of the glucose meal response half-life. Points represent the mean posterior value for each participant. (D) Average meal response profiles using the posterior mean parameter values. (E and F) Examples comparing the CGM data (blue) with the model prediction incorporating circadian dynamics (black) plus meal consumption (orange) for two participants with overdamped and underdamped dynamics, respectively. The time stamps of meals are shown as dashed lines. Participant order is the same in Figures 3A, 3B, 4A, and 4B. See also Figures S8 and S9.

The overall data modeling strategy is shown in Figures 2A–2C, where we first focus on ingestion events, glucose, and circadian rhythms (model 1, Figure 2A), then the relationships between the physical and the heart activity signals (model 2, Figure 2B), before finally adding interactions from the physical activity and heart signals to the glucose levels (model 3, Figure 2C). Based on the visual exploration (Figure 1) and physiological knowledge, we first built a minimal dynamical model of glucose levels (model 1) that included the following four features: (1) the ability to produce a continuous postprandial glucose response following an ingestion event, (2) negative feedback (representing the regulating action of insulin, depicted as a feedback loop in Figure 2A), (3) a random component that captures the glucose fluctuations between ingestion events and overnight, and (4) a circadian baseline rhythm (discussed in the next section).

These features were modeled with a system of stochastic differential equations (SDEs) (see STAR Methods), where ingestion events act to perturb glucose to higher levels. We refer to the increase in glucose levels relative to the baseline level caused by meals as the “meal height” (Figure 2D). As meals can cause different glucose responses according to their content, we allow each meal with a unique text entry to have a separate meal height parameter and report the mean across all meals for each participant. After ingestion causes a glucose increase, glucose levels return to their steady-state values (reflecting homeostasis). The decay kinetics and precise shape of the response will depend on the parameters of the model (Figure 2D), which are learned for each participant. Specifically, this individual-specific response to a meal perturbation can be summarized with three parameters: a half-life reflecting the time taken for glucose to return to baseline levels, the mean increase in glucose levels caused by meal consumption (referred to as the mean meal height), and a damping coefficient specifying whether the response profile is akin to an overdamped (a rapid glucose increase followed by a monotonous slower decay, i.e., non-dipping) or an underdamped (leading to a slower initial increase followed by decay and overshoot, i.e., dipping) response. To account for noisy fluctuations in the data, the glucose dynamics is also subjected to random perturbations in the corresponding SDE, meaning that the glucose time series data can show noisy deviations from the idealized meal response.

For each participant, the entire glucose time series is probabilistically matched (using exact likelihood calculations) to the model using a Gaussian state space model (a.k.a. a Kalman filter), and we infer each of the model parameters using Markov chain Monte Carlo (MCMC) sampling within a Bayesian framework that yields uncertainty estimates for each parameter (STAR Methods and supplemental information).

We first verified model performance by assessing the correlation coefficient between the fitted meal response function and the data (Figure S8). The correlation coefficient generally ranged from 0.5 to 0.8 but was particularly low for participant ID 04. Visual inspection of this participant’s raw data showed large glucose spikes following physical activity (Figure S9) and hence were not explained in this initial model, which we address below with more complex modeling. While all model parameters are shown in Figure S8, we here focus on three summary metrics of the glucose dynamics.

Response half-lives ranged from 1 to 2.2 h (Figure 3A), thus showing a dynamic range of 220%. The mean meal response heights ranged from 0.5 to 1.5 mmol/L above baseline (Figure 3B), and a more detailed examination revealed that glucose responses for a given individual vary according to the specific item consumed (Figure S9). The posterior parameter distributions for each participant (Figures 3A and 3B) quantify the uncertainty associated with the parameter estimates for each participant; in some cases the distributions were overlapping between two individuals, while in other cases the distributions were clearly separated (e.g., half-life comparing IDs 20 and 23, Figure 3A).^47^ Comparing parameter values across participants, we found a positive relationship between response half-lives and mean meal heights, with slower glucose response half-lives associated with larger postprandial glucose spikes (R = 0.44, p = 0.02, Figure 3C). This suggested that postprandial glucose control (i.e., the height of glucose spikes following meals) depends on glucose clearance time, which might be determined physiologically by insulin sensitivity or β cell function (discussion).

The damping coefficients describing the shapes of glucose responses were clustered around 0 across all participants (Figure S8), where values of 0 represent “critical” damping at the border between overdamped (non-dipping profiles, damping coefficient >0) and underdamped (profiles with a dip, damping coefficient <0). Interestingly, glucose responses were proposed to be critically damped in an early glucose model,^48^ which would be consistent with our finding that the inferred values are scattered around 0. However, we clearly find individual-specific response profiles, with participant ID 14 showing a distinct underdamped glucose response compared with the critically damped response in ID 23 (Figures 3D–3F). The inferred meal response and circadian time functions (orange) are smoother than the glucose data (blue, Figures 3E and 3F), but the full model that also adds random fluctuations produces glucose traces that closely resemble the glucose data (Figure S9).

The measured glucose CV, a metric of glycemic control used in clinical settings,^10^^,^^11^ showed significant associations with both the response half-lives (linear regression p = 0.03) and the average meal heights (p = 0.01, linear regression model R^2^ using both variables = 0.63). While the damping coefficient was not significantly associated with glucose CV, the individual shapes of glucose responses might play a role in other aspects of glucose dynamics such as overshooting and hunger.^49^ Our results highlight that glucose response half-lives play a role in glycemic control and may be a relevant metric for both fundamental research and clinical purposes.

### Circadian rhythms in baseline glucose levels are individual-specific

In addition to the input from ingestion events, the model also allows for an underlying circadian rhythm in glucose levels described with three parameters: a baseline level that specifies the glucose at the trough of the oscillation, an amplitude parameter denoting the difference between the trough and the peak of the oscillation, and the peak time of the oscillation. These circadian parameters are inferred for each individual jointly with the meal response parameters when fitting model 1 to the glucose data using MCMC (STAR Methods).

**Figure 4:**
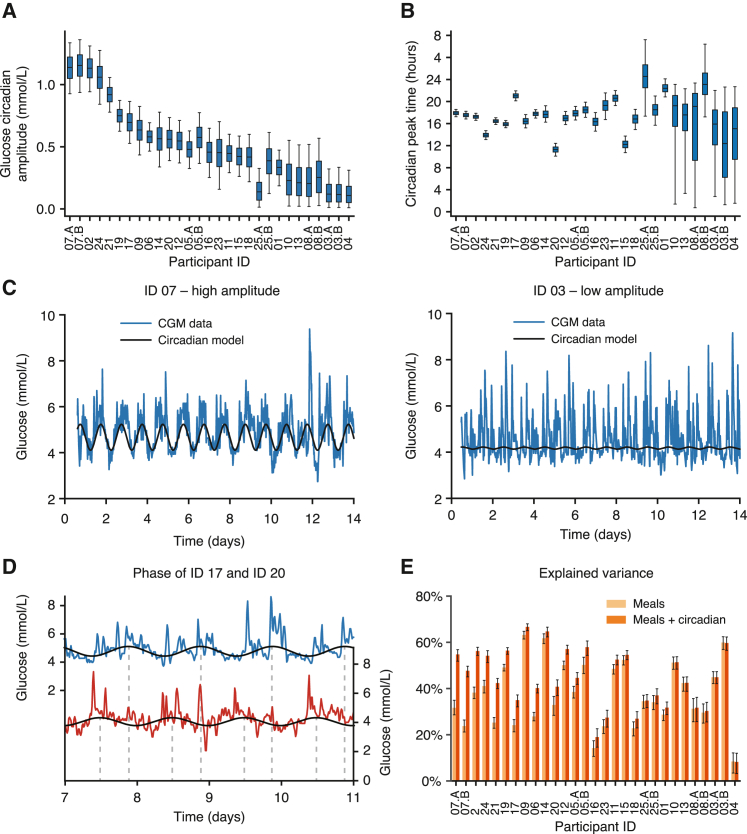
Amplitudes and peak times of circadian baseline levels of glucose are highly heterogeneous between participants (A) The amplitude of the 24-h sinusoidal circadian rhythm in baseline glucose levels after model fitting to the CGM data for all participants. The boxes represent the 25th, 50th (median), and 75th percentiles of the posterior distribution and the whiskers represent the 5th and 95th percentiles. (B) The circadian peak time of the glucose circadian rhythm across all participants. (C) Examples of participants with a high (ID 07) and a low (ID 03) amplitude glucose circadian rhythm. Blue, CGM data; black, fitted model of circadian baseline (using mean posterior parameter values). (D) Examples showing two participants with large phase difference in underlying glucose rhythm (ID 20 peak phase 10:00, ID 17 peak phase 20:00). (E) The explained variance in glucose levels using just the meal component of the model (light orange) compared with the inclusion of the circadian rhythm (dark orange). Error bars represent the 5th and 95th percentiles of the posterior distribution. Participant order is the same in Figures 3A, 3B, 4A, and 4B. See also Figure S10.

The amplitudes of underlying circadian glucose rhythms were participant specific (Figure 4A), being virtually null for some individuals, while exceeding 1 mmol/L for others (Figure 4C, IDs 03 and 07). Notably, the parameter uncertainty was small enough that there was no overlap in the estimates for IDs 03 and 07 (Figure 4A). To identify subjects whose profiles do not support a circadian baseline trend, we fitted an alternative model that lacked a circadian baseline and compared the two models using the Bayesian information criterion (BIC). For IDs 10, 13, 08, 25, 03, and 04 (which have the weakest amplitude according to Figure 4A), the BIC indicated evidence for the model lacking the circadian baseline, while the BIC favored the model with an additional circadian component for all remaining participants (Figure S10). The combination of the amplitude posterior estimates and heterogeneous model preference according to BIC thus suggests that circadian baseline glucose oscillations are individual-specific physiological characteristics.

The peak times of the glucose circadian oscillations similarly varied between participants (Figure 4B, with the same participant order as in Figure 4A), with peak times of the circadian baselines typically falling around the mid-afternoon, but with significant shifts in some individuals. For example, participant ID 20 had a peak time at 10:00, while it occurred much later for participant ID 17, falling at 20:00 (Figure 4D). The peak time distributions showed tight confidence intervals for participants with large amplitudes and wide intervals for participants with weaker amplitudes (Figure 4B). This relationship is probably caused by a lower signal-to-noise ratio for participants with a low circadian amplitude.

Overall, the underlying circadian glucose rhythm can explain >15% of glycemic variability in addition to the meal model for participants with large amplitudes (Figure 4E). While we have not tested whether it would be possible to modify either the peak time or the amplitude of this rhythm, these personalized parameters should prove to be useful in applications such as personalized meal timing (discussion).

### HR is well predicted by physical activity and time of day, but the predictability of HRV varies between individuals

We next focused on the physical activity, HR, and HRV data, where we aimed to model the dependencies between the variables and quantify the ability of subsets of the three signals to explain the variance of others, in addition to the contribution of circadian oscillations. For this, we created a new model (model 2, Figure 2B) that incorporated the influence of physical activity on HR and HRV, and we used MCMC to sample from model parameters and quantify differences between individuals (all parameters shown in Figure S11).

**Figure 5:**
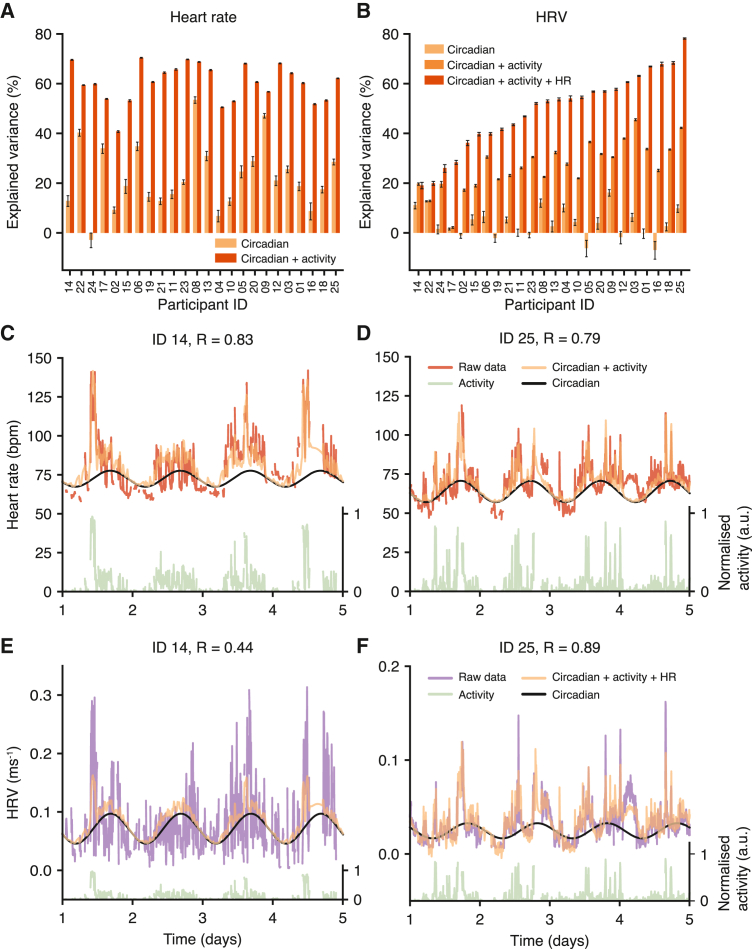
HRV predictions using multisignal inputs and circadian rhythms are more heterogeneous than for HR (A) The amount of variance of the HR signal explained by circadian rhythms (light orange) and a combined model with circadian rhythms and physical activity (dark orange). Error bars represent the 5th and 95th percentiles of the posterior distribution. (B) The amount of variance of the HRV (RMSSD^−1^) signal explained by circadian rhythms (light orange), a combined model with circadian rhythms and physical activity (medium orange), and a combined model with circadian rhythms, physical activity, and HR (dark orange). (C and D) Examples comparing HR data with model predictions for two participants. Red, HR data; black, baseline circadian rhythm; green, physical activity (shown on normalized scale where 1 represents the maximum value); orange, model prediction with circadian rhythm and integrating activity. (E and F) Examples comparing HRV data with model predictions for two participants. Purple, HRV data; black, baseline circadian rhythm; green, physical activity (shown on normalized scale where 1 represents the maximum value); orange, model prediction using circadian rhythms, physical activity, and HR. Participant order is the same in (A) and (B). See also Figure S11.

For HR, the combination of a circadian trendline and physical activity as two inputs was consistently predictive, explaining 40%–65% of HR variance across all participants (Figure 5A). Figures 5C and 5D show an example of the predicted HR (orange) for two different participants using the underlying circadian trend (black) and integrating the physical activity (green). While the circadian contribution to the explained HR variance differs for these two participants (Figure 5A), the correlation between the predicted and the observed HR was ∼0.8 for both participants, demonstrating that time of day and activity state are necessary for optimal personalized modeling of HR, which is consistent with previous studies.^50^

The predictability of HRV was, in contrast, much more heterogeneous between participants, with total variance explained between 20% and 80% (Figure 5B). This notable difference is illustrated with two participants, showing a favorable prediction for participant ID 25 (R = 0.89, Figure 5F) compared with ID 14 (R = 0.44, Figure 5E). In addition to inputs from physical activity and the circadian trend, we evaluated whether the correlations between HR and HRV could be exploited by using HR to predict HRV (which is technically more difficult to measure than HR). The dependence between HR and HRV showed marked interindividual differences, where for ID 25 the HR signal explained 40% of the variance compared with using only activity and circadian trend, but for ID 14 the addition of HR made no difference in HRV prediction (Figures 5E, 5F, and S11). Given that HR and HRV receive different inputs from the SNS and PNS,^25^ the strength of this dependence may be a function of the autonomic nervous system. Of note, ID 14 was previously diagnosed with diabetes (currently treated only with lifestyle measures and not pharmacological treatment), and autonomic dysfunction is a known complication of diabetes.^51^

### Integrating physical and heart activity signals helps explain glycemic dynamics

As a final modeling step, we integrated the physical and heart activity signals with the glucose-ingestion model to quantify how much of the glucose dynamics can be accounted for with physical activity, HR, and HRV (model 3, Figure 2C). To simplify the model inference, the parameters describing the physical and heart activity model in isolation (model 2, Figure 2B) were locked to their posterior mean values, and we added three new parameters describing the input of physical activity, HR, and HRV on glucose levels, respectively (model 3, Figure 2C). These influences were left unconstrained and could have positive, negative, or zero effect on glucose levels.

**Figure 6:**
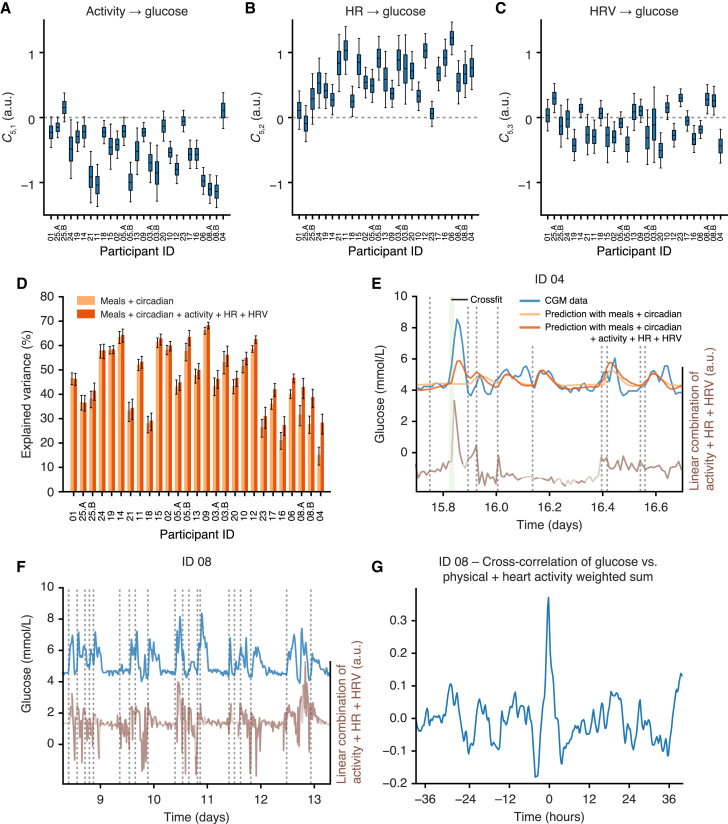
Adding physical activity, HR, and HRV into the glucose dynamics model can help explain glucose dynamics (A–C) Posterior distributions (shown as boxplots) of the model coefficients *C*_5,1_, *C*_5,2_, and *C*_5,3_ across all participants, which correspond to the influence on glucose of physical activity, HR, and HRV, respectively. The boxplots represent the 25th, 50th (median), and 75th percentiles of the posterior distribution and the whiskers represent the 5th and 95th percentiles. (D) A comparison of the variance explained in the glucose signal using just the meal and circadian rhythm model (light orange) compared with the prediction that also incorporates physical activity, HR, and HRV (dark orange). Error bars represent the 5th and 95th percentiles of the posterior distribution. (E) Example from ID 04 shows that the physical and heart activity data partially explain an exercise-induced glucose spike. Blue, glucose data; light orange, prediction using meal and circadian model components; dark orange, prediction including meal and circadian model components, physical activity, HR, and HRV; green shade, recorded activity events; brown, the weighted sum of the physical activity, HR, and HRV variables according to the inferred coefficients *C*_5,1_, *C*_5,2_, and *C*_5,3_; vertical dashed lines, ingestion events. (F) Example from ID 08 showing how glucose dynamics (blue) track with the weighted sum of the physical activity, HR, and HRV variables according to the inferred coefficients *C*_5,1_, *C*_5,2_, and *C*_5,3_ (dark brown, data only; light brown, using filtered estimations from model 2 to fill missing physical and heart activity data; vertical dashed lines, ingestion events). (G) The cross-correlation of the glucose (blue in F) with the weighted sum of the physical activity, HR, and HRV variables (brown in F) using all recorded data. Participant order is the same between (A), (B), and (C). See also Figure S12.

Model fitting revealed that the effect of physical activity accelerometer counts on glucose (parameter *C*_5,1_) was generally negative, the effect of HR (parameter *C*_5,2_) was generally positive, and the effect of HRV (parameter *C*_5,3_) was typically neutral across all participants (Figures 6A–6C). Given that we observed increased glucose levels during some periods of exercise during data visualization (Figure 1B), the negative influence of physical activity accelerometer counts on glucose as revealed by the model parameter C_5,1_ (Figure 6A) was not expected. To test the robustness of this prediction, we therefore re-fitted the data using three simpler models, where there was only one input at a time from the physical and heart activity signals (Figure S12). The influence of physical activity on glucose remained negative, even when it was the sole input from model 2 into glucose levels, further suggesting that the overall dominating effect of physical activity is to deplete glucose levels among the participants of our study. Meanwhile, as HR acts to increase glucose levels (*C*_5,2_), increased HR during intense exercise can still lead to a net increase in predicted glucose levels.

We next quantified the importance of heart and physical activity for glucose predictions by comparing the variance explained with meals and circadian rhythms compared with all components, including physical activity, HR, and HRV (Figure 6D). Overall, the amount of additional explained variance by the physical and heart activity signals was modest for most participants but contributed up to 15% in some individuals (Figure 6D).

Among the participants whose glucose dynamics benefit most from the physical and heart activity signals, the mode of action also differed. For participant ID 04, where the prediction of glucose was the lowest with model 1 (Figure 4E), the inclusion of physical and heart activity signals allowed for partial prediction of exercise-induced glucose spikes that were otherwise absent from the glucose model that contained meals only (Figure 6E). For participant ID 08, there were no notable isolated glucose spikes that could be predicted by the physical and heart activity data (Figure 6F). Instead, the linear combination of the physical and heart activity variables (weighted according to the inferred coefficients *C*_5,1_–*C*_5,3_) appeared to track the glucose levels (Figure 6F), and the cross-correlation profile showed a maximum correlation between the two signals without time delay (Figure 6G). This suggests that for this mode of action, the physical and heart activity signals contribute diffusely to capture glucose baseline trends spread over the time series.

## Discussion

This study makes two contributions to the quantitative analysis of glucose dynamics in terms of study design and computational analysis. First, we used several wearable biosensors to measure multiple, interconnected data streams simultaneously. Inferring dynamical models would not be possible without such high-resolution time series. On a practical level, the main advantages of health measurements with wearable biosensors are their efficiency, non-invasiveness, and relative simplicity: in our study it took a team of two researchers 2 days to complete questionnaires and set up devices to record data for all 25 participants simultaneously. Second, we developed a data analysis method that combines stochastic dynamical modeling with Bayesian inference to learn personal parameters along with their associated uncertainty, and this parameter uncertainty was necessary to compare participants. The main insight of such personalized modeling is the possibility to robustly quantify individualized response patterns across a range of parameters and metrics, from the glucose half-lives and circadian oscillations, the coupling between HR and HRV, to the benefit of adding additional signals to predicting glucose.

The output of this modeling has wide implications for understanding both the biological underpinnings of cardiometabolic dysfunction as well as consequences for the use of wearables in a clinical setting. We first started by combining the food and drink events with the glucose time series, where we found that slow glucose dynamics are associated with large postprandial glucose spikes. Mechanistically, slow glucose disposal could relate to the quantity of ingested carbohydrates, the rate of gut absorption,^52^ its metabolism,^53^ the suppression of endogenous glucose production,^54^ insulin resistance, or β cell function.^2^

We also detected highly personalized circadian rhythms in the baseline glucose levels by quantifying amplitude and peak-time parameters in each individual along with uncertainty estimates (Figures 4A and 4B). While diurnal rhythms in β cell function and insulin sensitivity have previously been shown within healthy populations,^55^^,^^56^^,^^57^ it was unexpected to find significant differences in the amplitudes and phases of circadian oscillations in baseline glucose levels between healthy individuals. The extent to which these 24-h rhythms are determined by central or peripheral clocks or whether they are largely entrained by meal timing^58^ cannot be deduced without further information. Future studies will determine whether this circadian glucose baseline trend is predictive of responses to specific meal times, e.g., in time-restricted eating, an intervention that restricts eating to a specific window within the 24-h clock.^59^

Our results also have practical implications for clinicians as the physical and heart activity data explained up to 15% of glucose variability in our study, although this was highly variable between participants. From a clinical perspective, the incorporation of these additional physical and heart activity signals for some individuals might help both patients and clinicians understand glucose dynamics that seem otherwise disconnected from meal consumption (e.g., Figure 6E). As outlined in the motivation paragraph, clinicians and diabetic patients can link glucose excursion with ingestion events and intensive physical activity but find it challenging to do so for the remaining glucose dynamics observed throughout the day.

There are multiple possible approaches to modeling multimodal data such as we collected, and the particular structure of the glucose model has often been dictated by the data available and by the stated goal.^34^^,^^35^^,^^36^ Models based on differential equations range from simple, minimal models^37^ to mechanistically detailed descriptions that include more variables, more spatial compartments, and dozens of additional parameters.^38^ Inspired by these more complex models, there are many possible extensions that could be added to our glucose model, such as glucose absorption rates of mixed meals due to food content in carbohydrates, but also fat, fiber, and protein contents, which are known to slow down nutrient absorption,^14^ although the addition of meal-specific response shapes would effectively double the number of meal-related parameters. In addition to differential equation models, time series methods such as wavelets can also be applied to multimodal continuous wearable signals to detect, for example, changes in period and amplitude over time, to identify transient events, to assess associations between signals, and to perform signal denoising.^60^^,^^61^^,^^62^ Explicit models and time series analysis methods are ultimately complementary tools that come with their own sets of advantages and limitations.

An area that has seen a broad spectrum of time series models concerns short-term glucose forecasting, typically for applications in closed-loop insulin delivery systems.^33^^,^^44^ Gaussian state-space models that are conceptually similar to ours but with more variables have been deployed in artificial pancreas devices.^63^^,^^64^ Machine learning methods, including support vector machines (SVMs) and neural networks, have also been used for short-range forecasting,^39^^,^^40^ where the advantage of such methods is that more complex non-linear dependencies and long-range memory can be captured. As our focus was on explaining the total time series rather than short-term forecasting, we here traded some of this flexibility for explainability by assuming a relatively simple dynamical model with interpretable components and parameters. In the future, we envisage several applications, such as larger-scale epidemiological studies (e.g., do inferred parameters track with health state?) and clinical trials to see whether parameters change in response to an intervention. Particularly for clinical trials, point estimation of parameters is not adequate, and uncertainty estimates are required to perform statistical tests for a given individual, which we achieve here through MCMC. In health care, there is increasing interest in digital twins^65^^,^^66^ to integrate multiple clinical data streams, devise personalized treatments, and perform risk modeling. As our approach contains interpretable parameters, it lends itself readily to simulating hypothetical situations (e.g., by altering circadian amplitude or glucose response time). Overall, our method transforms a multivariable wearable data input into a series of metrics that describe the dependencies between physiological variables, including the relaxation timescales after external perturbations and circadian properties, and this approach provides a platform for probing physiological changes across circadian perturbations, aging, and cardiometabolic disorders.

### Limitations of the study

Our study population was young and in good health overall, and we lack additional, more detailed health information or standard clinical metrics such as glycated hemoglobin (HbA1c). Since we opted here for a small-scale study with a focus on the methodology and models to combine multiple wearable sensors, we have sought to identify differences between individuals without attempting to associate them with either good or bad health outcomes. Future studies that use larger and more diverse cohorts could use the proposed method to relate the inferred personal glycemic parameters to lifestyle, environmental, or genetic factors, and it would also be useful to explore whether candidates that would benefit from inclusion of physical and heart activity to glucose modeling can be predicted in advance.

With respect to the modeling, a potential limitation of our approach is the use of a linear differential equation model, which may not be able to capture more complex phenomena such as eventual decreases in hepatic glucose production during prolonged exercise.^67^ A recent study based on deep learning found that the addition of wristband activity data improved the root-mean-square error of 60-min glucose forecasting by 2.25 mg/dL (0.1 mmol/L) from a baseline of 35.3 mg/dL (2.0 mmol/L), and hence, more substantial improvements in glucose predictions may prove to be a difficult challenge even with more flexible models.^68^

## STAR★Methods

### Key resources table

**Table:** 

REAGENT or RESOURCE	SOURCE	IDENTIFIER
**Deposited data**		

Food app, CGM and ActiHeart data	This paper	https://github.com/naef-lab/MultiSensor https://doi.org/10.5281/zenodo.8028677

**Software and algorithms**		

Python version 3.7.4	Python Software Foundation	https://www.python.org
The MultiSensor Study code	This paper	https://github.com/naef-lab/MultiSensor https://doi.org/10.5281/zenodo.8028677

### Resource availability

#### Lead contact

Further information and requests for resources should be directed to and will be fulfilled when possible by the lead contact, Felix Naef (felix.naef@epfl.ch).

#### Materials availability

This study did not generate new physical materials.

### Experimental model and study participant details

The Multi-Sensor Study (MSS) was approved by the local ethics committee (CER-VD, BASEC no. 2019-02245) and each participant signed a written informed consent. Recruitment was performed via posters at the École Polytechnique Fédérale de Lausanne (EPFL), Lausanne University Hospital (CHUV) and the University of Lausanne (UNIL) and via presentations given in the EPFL School of Life Sciences.

We included adults aged ≥ 18 years, with a smartphone compatible with the myCircadianClock app (iOS or Android systems^45^) and able to take pictures of food/drinks, and who self-identified as disciplined enough and motivated to record all data for two weeks. The exclusion criteria were major illness/fever, surgery over the previous month, eating disorder, major mental illness, unable to give informed consent, taking medicines including paracetamol, aspirin or vitamin C supplements, enrolled in another interventional clinical trial (medication, medical device), shift work or travel to a different time zone before and during the study. 25 participants (16 males, 9 females) were recruited, and participant characteristics are shown in Figure S1.

### Method details

#### Devices and experimental design

At baseline, we collected data on demographics, medical history, physical activity (short form of International Physical Activity Questionnaire, IPAQ-SF),^69^ chronotype (The Munich ChronoType Questionnaire),^70^ sleeping habits (Pittsburgh Sleep Quality Index)^71^ and eating timing (with a custom questionnaire on eating habits during work and free days).

For each participant, we collected data for two weeks using the following devices: 1) Timestamps of food/drinks and text annotations collected with the smartphone application (app) myCircadianClock^45^; 2) Continuous glucose monitoring (CGM) using the Abbott FreeStyle Libre Pro device; 3) Physical activity, heart rate (HR) and heart rate variability (HRV using RMSSD^–1^) using the CamNtech ActiHeart device version 5. Participants were instructed to take pictures of all consumed food and drink with the research-dedicated myCircadianClock smartphone. Recorded entries included a timestamped picture and a free-text annotation, and entries with the same annotation were considered as the same meal type. Participants could annotate photographs either immediately or in the following hours. Optionally, participants could type text-only entries without any picture, e.g., if the smartphone ran out of battery, or if it was not socially acceptable to take pictures in the current context. Participants were also asked to optionally log physical exercise using the app. While the CamNtech Actiheart device is waterproof, participants were permitted to briefly remove the device during showers and baths. Specific information on individual device technical failure, handling of missing data and data quality is included in the Supplementary Information (Methods S1).

#### Pre-processing CGM data

We used nonparametric regression with Gaussian processes (GPs) to remove the long-term trends observed in the data. After mean-centring the data, we fitted a GP with a squared exponential kernel $K_{SE}\left(t,t^{\prime }\right)=\left(-{\left|t-t^{\prime }\right|}^{2}/2l^{2}\right)$ and a length scale *l*=48 hours using GPflow.^72^

#### Data analysis

See the Supplementary Information (Methods S1) for detailed computational methods which are summarised here. We use a linear Gaussian state space model (otherwise known as a Kalman filter^73^) to analyse the time series generated by the wearable devices, which was implemented using the ‘LinearGaussianStateSpaceModel’ distribution within TensorFlow Probability.^74^ We will first describe the general data analysis framework before providing details on each of the three models used (Models 1-3). For each model we define a dynamic model that describes the time evolution of the underlying physiological variables and a measurement model that incorporates measurement noise. For the dynamic model, we use a system of stochastic differential equations (SDEs).

(Equation 1) dx(t)=Wx(t)dt+dβ,

where W is a matrix describing the interactions between the variables x(t), and β is a brownian noise term with covariance matrix Q. The specific forms of W and Q are unique for each model and will be described below. To keep the model exact while benefiting from the generic framework of Gaussian state space models (a.k.a. as Kalman filters), we then convert this system of continuous-time SDEs into a model where time is discrete (see Methods S1 for details).

(Equation 2) $$x\left(t_{k}\right)=F_{k-1}x\left(t_{k-1}\right)+N\left(0,\Sigma_{k-1}\right)\text{,}$$

where $F_{k}$ is the state-transition model and $\Sigma_{k}$ is the covariance of the process noise. The measurement model describes the observation process and assumes that variables are observed with normally distributed measurement noise

(Equation 3) $$y\left(t_{k}\right)=H_{k}x\left(t_{k}\right)+N\left(m_{k},R_{k}\right)\text{,}$$

where $H_{k}$ is the observation matrix and $m_{k}$ and $R_{k}$ represents the mean and covariance of the observation noise, respectively. The goal is to use the wearable time series data $y_{1:T}$ to estimate parameters (denoted by θ) for each participant. Within a Bayesian inference framework, the parameters of the model can be estimated from the data as follows

(Equation 4) $$p\left(\theta |y_{1:T}\right)\propto p\left(\theta \right)p\left(y_{1:T}|\theta \right)\text{,}$$

where p(θ) is the prior distribution of parameters and $p\left(y_{1:T}|\theta \right)$ is the likelihood of observing the temporal data $y_{1:T}$ given the set of parameters θ. Considering the time series sequence of data, the likelihood term for a given set of parameters θ can be expressed as

(Equation 5) $$p\left(y_{1:T}|\theta \right)=p\left(y_{1}|\theta \right)\prod_{k=2}^{T}p\left(y_{k}|y_{1:k-1},\theta \right)\text{,}$$

and the sequence of distributions $p\left(y_{k}|y_{1:k-1},\theta \right)$ are calculated within a Kalman filtering framework. Once the likelihood and priors are specified for each model, we used the Hamiltonian Monte Carlo sampler provided within TensorFlow Probability to sample model parameters from the posterior distribution (described below in quantification and statistical analysis). The priors for all models are specified in Methods S1.

##### Model 1: Glucose model

We model glucose dynamics (Figure 2A) with a two-dimensional system of SDEs, where the second variable $x_{\text{GLUC}2}$ represents the glucose levels and the first variable $x_{\text{GLUC}1}$ represents an unobserved latent variable that allows negative feedback within the system. In matrix form, the model is expressed as follows

(Equation 6) $$\begin{array}{c} dx\left(t\right)=Wx\left(t\right)dt+d\beta \text{,}\\ x\left(t\right)=\left[\begin{array}{c} x_{\text{GLUC}1}\\ x_{\text{GLUC}2} \end{array}\right]\text{,}\\ W=\left[\begin{array}{cc} {-A}_{11} & {-A}_{12}\\ A_{21} & {-A}_{22} \end{array}\right]\text{,}\\ Q=\left[\begin{array}{cc} 0 & 0\\ 0 & B_{22} \end{array}\right]\text{,} \end{array}$$

and where the coefficients $A_{ij}$ are constrained to be positive. The covariance of the brownian noise term β is given by Q. The ‘damping coefficient’ is determined by whether the eigenvalues of the matrix W are real or complex. For the 2x2 matrix W, this damping coefficient can be determined by $-\det\left(W-I\;\text{tr}\left(W\right)/2\right)/{\left(\text{tr}\left(W\right)/2\right)}^{2}$.

The smartphone application provides a list of the recorded ingestion event times ${\left\{t_{m}\right\}}_{m=1}^{M}$ for a total of M meals. We incorporate meal events (recorded at time $t_{m}$) as producing a response function $r_{m}\left(t,t_{m},\theta \right)$ by perturbing the first variable $x_{\text{GLUC}1}$ to higher values (see Methods S1 for precise functional form), and then the total meal function is the sum over all M individual meal responses

(Equation 7) $$r\left(t\right)=\sum_{m=1}^{M}r_{m}\left(t,t_{m},\theta \right)\text{.}$$

We define the glucose half-life parameter as the model-predicted time to return to 0.5 mol/L after a standardised increase of 1mmol/L. We also add an underlying circadian trend in glucose levels using a sinusoidal function

(Equation 8) $$g_{\text{GLUC}}\left(t\right)=A_{0,\text{GLUC}}+A_{1,\text{GLUC}}\left(1+\cos\;\left(\omega t-\varphi_{\text{GLUC}}\right)\right)/2$$

where $A_{0,\text{GLUC}}$ is the baseline level, $A_{1,\text{GLUC}}$ is the amplitude, ω is the frequency (fixed at 2π/24), and $\varphi_{\text{GLUC}}$ is the peak time of the maximum. The observation model for the glucose model is then as follows

(Equation 9) $$\begin{array}{c} y\left(t_{k}\right)=H_{k}x\left(t_{k}\right)+N\left(m_{k},R_{k}\right)\text{,}\\ H_{k}=\left[0\;1\right]\\ m_{k}=r\left(t\right)+g_{\text{GLUC}}\left(t\right)\text{,}\\ R_{k}=\sigma_{\text{GLUC}}^{2}\text{,} \end{array}$$

We compared Model 1 with an alternative version without circadian oscillations using the Bayesian Information Criterion (BIC) $\text{BIC}=k\;\ln\left(n\right)-2\;\ln\left(p\left(y_{1:T}|\theta \right)\right)$, where k is the number of parameters and n is the number of data points. We calculated the difference in BIC score using Model 1 both with and without a circadian components and used a cut-off of 2ln⁡(10) to indicate that the strength of evidence favoured a particular model.^75^

##### Model 2: Physical and heart activity model

We model physical and heart activity dynamics (Figure 2B) with a three-dimensional system of SDEs, where the first variable $x_{\text{ACT}}$ represents physical activity, the second variable $x_{\text{HR}}$ represents heart rate and the third variable $x_{\text{HRV}}$ represents heart rate variability, where we use the inverse of the root mean square of successive differences between normal heartbeats (RMSSD^-1^). We normalise all three variables by their respective standard deviations before inferring parameters. In matrix form, the model is expressed as follows

(Equation 10) $$\begin{array}{c} dx\left(t\right)=Wx\left(t\right)dt+d\beta \text{,}\\ x\left(t\right)=\left[\begin{array}{c} x_{\text{ACT}}\\ x_{\text{HR}}\\ x_{\text{HRV}} \end{array}\right]\text{,}\\ W=\left[\begin{array}{ccc} {-C}_{11} & 0 & 0\\ C_{21} & {-C}_{22} & 0\\ C_{31} & 0 & {-C}_{33} \end{array}\right]\text{,}\\ Q=\left[\begin{array}{ccc} D_{11} & 0 & 0\\ 0 & D_{22} & \rho \sqrt{D_{22}D_{33}}\\ 0 & \rho \sqrt{D_{22}D_{33}} & D_{33} \end{array}\right]\text{,} \end{array}$$

and where the coefficients $C_{ij}$ are constrained to be positive and the covariance of the brownian noise term β is given by Q. In the model, the correlation in the fluctuations between HR and HRV is quantified with the correlation parameter ρ. The observation model is then given by

(Equation 11) $$\begin{array}{c} y\left(t_{k}\right)=H_{k}x\left(t_{k}\right)+N\left(m_{k},R_{k}\right)\text{,}\\ H_{k}=\left[\begin{array}{ccc} 1 & 0 & 0\\ 0 & 1 & 0\\ 0 & 0 & 1 \end{array}\right]\text{,}\\ m_{k}=\left[\begin{array}{c} g_{\text{ACT}}\left(t\right)\\ g_{\text{HR}}\left(t\right)\\ g_{\text{HRV}}\left(t\right) \end{array}\right]\text{,}\\ R_{k}=\left[\begin{array}{ccc} \sigma_{\text{ACT}}^{2} & 0 & 0\\ 0 & \sigma_{\text{HR}}^{2} & 0\\ 0 & 0 & \sigma_{\text{HRV}}^{2} \end{array}\right]\text{,} \end{array}$$

where $g_{\text{ACT}}\left(t\right)$, $g_{\text{HR}}\left(t\right)$ and $g_{\text{HRV}}\left(t\right)$ are circadian oscillatory functions (Methods S1).

##### Model 3: Combined model

The final model (Figure 2C) connects the physical and heart activity signals with CGM dynamics by stitching the previous glucose (Model 1) and physical and heart activity models (Model 2) together. Both models are otherwise left unchanged, but there is an introduction of three new parameters $C_{51},C_{52}$ and $C_{53}$ that describe the effect of physical activity, HR and HRV on glucose levels, respectively. These three parameters are left unconstrained and can take either positive or negative values. To simplify the model inference problem, the parameters from Model 2 describing the physical and heart activity model in isolation were locked to their posterior mean values.

(Equation 12) $$\begin{array}{c} dx\left(t\right)=Wx\left(t\right)dt+d\beta \text{,}\\ x\left(t\right)=\left[\begin{array}{c} x_{\text{ACT}}\\ x_{\text{HR}}\\ x_{\text{HRV}}\\ x_{\text{GLUC}1}\\ x_{\text{GLUC}2} \end{array}\right]\text{,}\\ W=\left[\begin{array}{ccccc} {-C}_{11} & 0 & 0 & 0 & 0\\ C_{21} & {-C}_{22} & 0 & 0 & 0\\ C_{31} & 0 & {-C}_{33} & 0 & 0\\ 0 & 0 & 0 & {-A}_{11} & {-A}_{12}\\ 0 & 0 & 0 & A_{21} & {-A}_{22} \end{array}\right]\text{,}\\ Q=\left[\begin{array}{ccccc} D_{11} & 0 & 0 & 0 & 0\\ 0 & D_{22} & \rho \sqrt{D_{22}D_{33}} & 0 & 0\\ 0 & \rho \sqrt{D_{22}D_{33}} & D_{33} & 0 & 0\\ 0 & 0 & 0 & 0 & 0\\ 0 & 0 & 0 & 0 & B_{22} \end{array}\right]\text{,} \end{array}$$

The observation model is then given by

(Equation 13) $$\begin{array}{c} y\left(t_{k}\right)=H_{k}x\left(t_{k}\right)+N\left(m_{k},R_{k}\right)\text{,}\\ H_{k}=\left[\begin{array}{ccccc} 1 & 0 & 0 & 0 & 0\\ 0 & 1 & 0 & 0 & 0\\ 0 & 0 & 1 & 0 & 0\\ 0 & 0 & 0 & 0 & 1 \end{array}\right]\text{,}\\ m_{k}=\left[\begin{array}{c} g_{\text{ACT}}\left(t\right)\\ g_{\text{HR}}\left(t\right)\\ g_{\text{HRV}}\left(t\right)\\ {r\left(t\right)+g}_{\text{GLUC}}\left(t\right) \end{array}\right]\text{,}\\ R_{k}=\left[\begin{array}{cccc} \sigma_{\text{ACT}}^{2} & 0 & 0 & 0\\ 0 & \sigma_{\text{HR}}^{2} & 0 & 0\\ 0 & 0 & \sigma_{\text{HRV}}^{2} & 0\\ 0 & 0 & 0 & \sigma_{\text{GLUC}}^{2} \end{array}\right]\text{,} \end{array}$$

### Quantification and statistical analysis

The wearable data for each participant is analysed separately and the inferred parameters are presented along with the uncertainty for each individual as obtained through Markov Chain Monte Carlo (MCMC) sampling. The parameter posterior distribution was sampled using Hamiltonian Markov Chain Monte Carlo (HMC), which uses the gradients of the posterior to improve the efficiency of the sampling. To initialise the sampler, we found the maximum a posteriori probability (MAP) parameter estimate using the BFGS optimiser 'bfgs_minimize' within TensorFlow Probability. We then used the 'HamiltonianMonteCarlo' function with TensorFlow Probability with 5 leapfrog steps, and we scaled the step size of each variable to approximately match the standard deviation of the posterior distribution. To achieve this, we sampled posterior parameters using two steps. Firstly, we sampled 10,000 parameters (with a burn-in of 10,000 samples) using the 'SimpleStepSizeAdaptation' kernel to select the global step size, which adapts the global step size to achieve a target acceptance probability of 0.75.^76^ We then scaled the step size of each variable according to the standard deviation of this initial posterior distribution. Next, we resampled model parameters from the posterior distribution using 4 different chains with 10,000 samples each (with a burn-in of 10,000 samples), again using 'SimpleStepSizeAdaptation' kernel to globally rescale the step size. The 'SimpleStepSizeAdaptation' kernel was only applied to first 80% of the burn-in samples. From the MCMC samples, the percentiles of the posterior parameter distributions are shown graphically for each participant with boxplots. We then estimate the explained variance using $1-\text{Var}\left(y-\tilde{y}\right)/\text{Var}\left(y\right)$, using the model predictions $\tilde{y}$ from the MCMC parameter samples.

## Data Availability

De-identified participant data has been deposited at https://github.com/naef-lab/MultiSensor and at https://doi.org/10.5281/zenodo.8028677. All original code has been deposited at https://github.com/naef-lab/MultiSensor and at https://doi.org/10.5281/zenodo.8028677. Any additional information required to implement the method of this study is available from the lead contact upon request.

## Supplemental information

Document S1. Table S1 and Figures S1–S12 and Methods S1

Document S2. Article plus supplemental information
