# Supplementary material for: Uncovering personalized glucose responses and circadian rhythms from multiple wearable biosensors with Bayesian dynamical modeling
Source: Cell Rep Methods. 2023 Jul 31;3(8):100545. doi: 10.1016/j.crmeth.2023.100545 (PMC10475794; doi:10.1016/j.crmeth.2023.100545)
Supplement: Document S2. Article plus supplemental information [file mmc2.pdf]

Cell Reports
Methods

Article

Uncovering personalized glucose responses and circadian rhythms from multiple wearable biosensors with Bayesian dynamical modeling

Graphical abstract

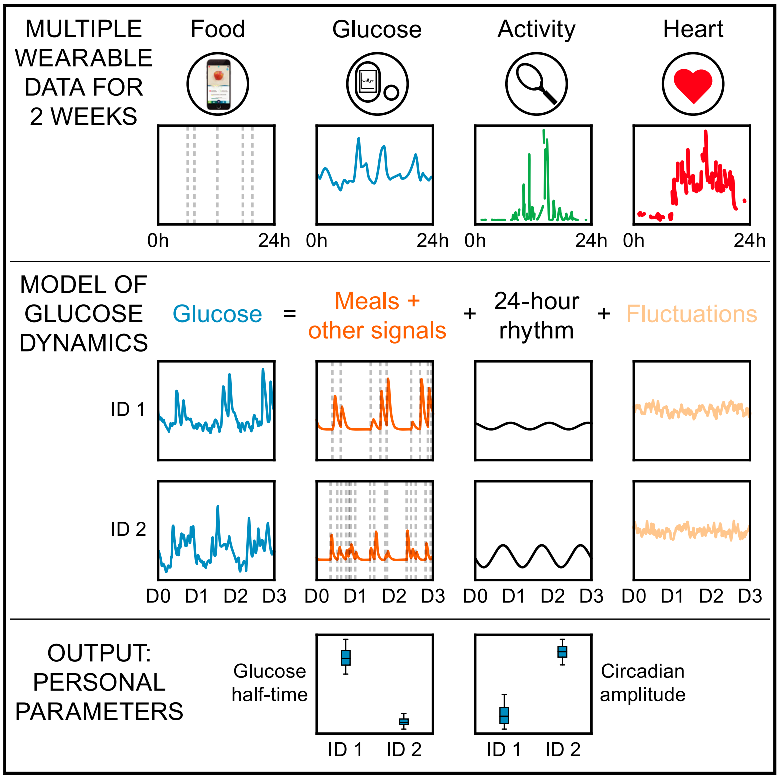

Highlights

- Simultaneous monitoring of food intake, glucose levels, and physical and heart activity
- Glucose dynamics modeled with meal responses plus a 24-h rhythmic baseline
- Model parameters learned on a personalized level with exact Bayesian inference
- Addition of physical and heart activity helps better explain glucose variability

Authors

Nicholas E. Phillips, Tinh-Hai Collet, Felix Naef

Correspondence

tinh-hai.collet@hcuge.ch (T.-H.C.),
felix.naef@epfl.ch (F.N.)

In brief

Phillips et al. simultaneously measure consumed food and drinks, continuous glucose levels, and physical and heart activity and use dynamical modeling to integrate these data streams. The method uses Bayesian inference to learn personalized parameters related to the dynamical responses to external stressors and 24-h rhythms of the wearable signals.

Phillips et al., 2023, Cell Reports Methods *3*, 100545
August 28, 2023 © 2023 The Authors.
https://doi.org/10.1016/j.crmeth.2023.100545

CellPress

Cell Reports Methods

CellPress
OPEN ACCESS

Article

Uncovering personalized glucose responses and circadian rhythms from multiple wearable biosensors with Bayesian dynamical modeling

Nicholas E. Phillips,[1,2] Tinh-Hai Collet,[2,3,4,*] and Felix Naef[1,4,5,*]

[1]Institute of Bioengineering, School of Life Sciences, Ecole Polytechnique Fédérale de Lausanne (EPFL), 1015 Lausanne, Switzerland
[2]Nutrition Unit, Service of Endocrinology, Diabetology, Nutrition and Therapeutic Education, Department of Medicine, Geneva University Hospitals (HUG), 1211 Geneva, Switzerland
[3]Diabetes Centre, Faculty of Medicine, University of Geneva, 1211 Geneva, Switzerland
[4]These authors contributed equally
[5]Lead contact
*Correspondence: tinh-hai.collet@hcuge.ch (T.-H.C.), felix.naef@epfl.ch (F.N.)
https://doi.org/10.1016/j.crmeth.2023.100545

MOTIVATION Wearable biosensors measure physiological variables with high temporal resolution over multiple days and are increasingly employed in clinical settings, such as continuous glucose monitoring in diabetes care. Such datasets bring new opportunities and challenges, and patients, clinicians, and researchers are today faced with a common challenge: how to best summarize and capture relevant information from multimodal wearable time series? Here, we aim to provide insights into individual glucose dynamics and their relationships with food and drink ingestion, time of day, and coupling with other physiological states such as physical and heart activity. To this end, we generate and analyze multiple wearable-device data through the lens of a parsimonious mathematical model with interpretable components and parameters. A key innovation of our method is that the models are learned on a personalized level for each participant within a Bayesian framework, which enables the characterization of interindividual heterogeneity in features such as the glucose response time following meals or underlying circadian baseline rhythm. This framework may prove useful in populations at risk for cardiometabolic diseases.

SUMMARY

Wearable biosensors and smartphone applications can measure physiological variables over multiple days in free-living conditions. We measure food and drink ingestion, glucose dynamics, physical activity, heart rate (HR), and heart rate variability (HRV) in 25 healthy participants over 14 days. We develop a Bayesian inference framework to learn personal parameters that quantify circadian rhythms and physiological responses to external stressors. Modeling the effects of ingestion events on glucose levels reveals that slower glucose decay kinetics elicit larger postprandial glucose spikes, and we uncover a circadian baseline rhythm for glucose with high amplitudes in some individuals. Physical activity and circadian rhythms explain as much as 40%–65% of the HR variance, whereas the variance explained for HRV is more heterogeneous across individuals. A more complex model incorporating activity, HR, and HRV explains up to 15% of additional glucose variability, highlighting the relevance of integrating multiple biosensors to better predict glucose dynamics.

INTRODUCTION

Wearable biosensors and smartphone applications are increasingly used to measure multiple physiological variables, including glucose levels, food consumption, and physical and heart activity. In contrast to traditional lab measurements taken at a single time point, the high-resolution wearable time series data record dynamic changes of physiological variables in response to external perturbations and as a function of the time of day. While these wearable data have the potential to provide a dynamic view of health states,[1] a major challenge in both clinical and research settings is how to extract physiologically relevant information from wearable time series data, and, in particular, when multiple data modalities are combined.

© 2023 The Authors.
This is an open access article under the CC BY license (http://creativecommons.org/licenses/by/4.0/).

Glucose regulation is a prime example of a dynamic and complex physiological system, as the body is confronted with irregular inputs (i.e., food intake, especially of carbohydrates) and controlled glucose uptake by organs (e.g., muscles, liver). As such, glycemic regulation employs a range of homeostatic mechanisms, including the glucose-insulin negative feedback loop, whereby insulin secretion by the pancreas is tightly regulated to avoid both low (hypoglycemic) and high (hyperglycemic) levels of glucose.[2,3] Understanding glucose regulation is important for human health, as long-term chronic hyperglycemia in diabetes can lead to micro- and macrovascular complications,[4] and glucose levels show a non-linear association with vascular diseases even in populations without diabetes.[5–7]

As glucose homeostasis is inherently dynamic and glucose levels fluctuate throughout the day, continuous glucose monitors (CGMs) have gained popularity due to their high temporal resolution. CGMs measure glucose in interstitial fluid continuously for up to 10–14 days with satisfactory clinical accuracy compared with reference capillary blood glucose values.[8,9] Standardized CGM-derived metrics such as the coefficient of variation (CV) and the time in range (the fraction of time spent within the desired range of 3.9–10.0 mmol/L, or 70–180 mg/dL) have been adopted in clinical practice to assess glycemic control in diabetes with insulin treatment.[10–13] At a more fine-grained level, CGMs have been combined with smartphone records of ingestion events to predict postprandial (postmeal) glycemic responses (PPGRs), where the PPGR is often defined as the area under the glucose curve for the 2 h following a recorded ingestion event.[14–16]

Nonetheless, neither the standardized CGM metrics nor the PPGR approach provides a complete picture of the entire glucose time series and its fluctuations over the 24-h clock. Physiological processes in humans, including glucose metabolism, follow circadian rhythms,[17–20] and responses to oral glucose tests are more pronounced in the evening than in the morning.[21] A pre-breakfast rise in glucose levels, termed the "dawn phenomenon," has been observed since the early 1980s and is often linked with a concomitant early morning rise of cortisol,[22,23] but the amplitude and phase of circadian rhythms in baseline glucose levels have thus far not been well described at an individual level. Identifying the relative contribution of the circadian rhythms to the glucose time series would be particularly helpful for the interpretation of 24-h CGM reports, which are often discussed with patients to identify patterns of low and high glucose values and to guide treatment.[24]

In addition to glucose, other physiological responses are accessible with biosensors, such as heart rate (HR; beats per minute) and heart rate variability (HRV), where HRV is typically quantified with metrics such as the root-mean-square of successive differences (RMSSD) between heart beats.[25] Epidemiological data have linked low HRV with high glucose levels,[26,27] and a reduction in HRV has been shown to predict the development of autonomic neuropathy before symptom onset among diabetic patients.[28] The simultaneous measurement of HR and HRV can provide insights into the autonomic nervous system activity,[29] as HR receives inputs from both the sympathetic nervous system (SNS; the "flight or fight" response) and parasympathetic nervous system (PNS; the "rest and digest" response), while the HRV-derived RMSSD metric is dominated by the PNS via vagal nerve activity.[30] Both HR and HRV are modulated by physical activity, which can now also be conveniently measured with a triaxial accelerometer. Furthermore, it is known that glucose levels are affected during exercise, which motivates attempts to connect physical activity from wearable device signals to continuous glucose data.[31–33]

Regarding the analysis of wearable data streams, a diverse range of glucose models have been proposed over the last decades,[34–36] ranging from minimal models[37] to more detailed simulators with dozens of parameters[38] and neural networks.[39,40] Recent efforts have also attempted to utilize additional multimodal wearable signals to either improve glucose forecasting or provide more accessible proxies for glucose without using CGMs.[33,41–43] Many of these methods are specialized toward short-range forecasting, which is certainly useful in applications like the artificial pancreas.[44] In a different context, researchers and clinicians need new wearable data analysis tools to perform statistical comparisons between individuals and quantify changes in glucose regulation across multiple time points and different disease states, but such approaches to extract personalized summary metrics from the global recordings remain comparatively unexplored.

In this study, we acquired multiple wearable biosensor data to monitor food and drink ingestion, glucose excursions, physical activity, HR, and HRV in individuals in free-living conditions. Our aim was to quantify how external perturbations (such as ingestion events and physical activity) and baseline circadian rhythms determine temporal glucose levels on a personalized level. To this end, we develop data-driven computational models to analyze data streams from multiple wearables with distinct model components that capture the interactions between physiological variables, 24-h rhythms, and random fluctuations. Individual-specific parameters are learned in a Bayesian framework providing parameter uncertainties and enabling statistical comparisons between participants. We subdivide the problem of analyzing the multiple signals by creating three successive mathematical models that include different subsets of variables. Our three-tiered modeling reveals the high degree of personalization across a wide range of metrics, even within a healthy population, from glucose decay kinetics, circadian rhythms in baseline glucose levels, and the dependence between HR and HRV. Future studies will be able to re-use the framework to describe personalized longitudinal changes over time in response to interventions and to cardiometabolic diseases.

RESULTS

Measuring multivariable physiological time series in free-living conditions

To quantify the personalized dynamics of individuals in free-living conditions, we measured ingestion events, glucose levels, physical activity, HR, and HRV for 25 participants over a 2-week period. Participants (16 males, 9 females) were young (mean age 33.0 ± SD 11.0), had a normal weight (mean BMI 22.7 ± 2.8 kg/m^2; one person with overweight and one person with obesity), and had a normal blood pressure (systolic 117.6 ± 11.4 mm Hg, diastolic 75.3 ± 7.9 mm Hg) (participant

CellPress
OPEN ACCESS

characteristics shown in Figure S1). Participant ID 14 was previously diagnosed with diabetes, but currently treated only with lifestyle measures (and not pharmacological treatment), and hypertension, treated with perindopril. Participants were asked to record all food and drink consumption and add a manual free text annotation of the content with the smartphone application myCircadianClock.[45] Each ingestion event was automatically time stamped by the app. The adherence (defined as at least two meals separated by at least 5 h in a given day[46]) was above 83% for all participants (Table S1).

We measured glucose levels continuously using the Abbott FreeStyle Libre Pro CGM, which records interstitial glucose levels every 15 min over a 2-week period. As the device is blinded, participants were unable to access their glucose data during the study period, thus avoiding feedback on their eating behavior. Five participants wore two sensors (on different arms), with the aim of validating that parameters estimated from the model were consistent between the two sensors (noted ID A and B in the figures). Physical activity, HR, and HRV were measured for each participant over the 2-week study period using the CamNtech Actiheart version 5 device, and the physical and heart activity data were also blinded to participants during the study.

Multiple wearable time series data reveal complex dynamical responses as a function of external inputs and time of day

Before developing a detailed mathematical model, we performed initial data exploration to identify the key features that we wanted to capture in the model (overview of all data streams shown in Figures S2–S6). First, we superposed the recorded days of glucose data based on time of day and found marked individual-specific mean 24-h patterns, with the highest mean glucose levels occurring at different times of the day depending on the individual (Figure 1A; all participants shown in Figure S7). These unique 24-h trends could be caused by either food or drink ingestion (i.e., external perturbations) and/or an underlying circadian baseline trend in glucose. This motivated the inclusion of both ingestion events and circadian rhythms in the model of glucose dynamics as separate components.

Further exploratory analysis of the multiple wearable signals showed rich interactions between subsets of the five measured variables (i.e., ingestion events, glucose, activity, HR, and HRV). As expected, glucose levels often rose following ingestion events, and for some of the individuals, recorded meals seemed to lead to large, predictable peaks in glucose (Figure 1B, IDs 14 and 23), while others showed a more complex relationship, with small postprandial glucose spikes that were barely larger than the glucose fluctuations between meals (Figure 1B, ID 06). Based on these observations, and compared with CGM analysis methods that focus exclusively on PPGRs for 2–3 h,[14–16] our goal is now to dynamically model the entire glucose time series over 2 weeks, including the fluctuating glucose levels occurring overnight or during longer intervals between ingestion events.

Visual inspection of the physical and heart activity data showed that spikes in physical activity typically coincided with an increased HR and HRV (as measured with RMSSD^{-1}) (Figures 1C and 1D). By creating a joint dynamical model of the three signals (physical activity, HR, and HRV), we aimed to uncover the interindividual heterogeneity in the coupling between the multiple signals as well as the underlying circadian rhythms.

Finally, we observed spikes in glucose levels following physical activity for some individuals (Figures 1B–1D, ID 23), which could be caused by the release of glucose under the influence of adrenaline/epinephrine or glucagon. However, to establish more firmly whether physical and heart activity signals can explain glucose variation, we develop below a dynamical model to mathematically assess the extent to which the total glucose signal across the 2-week study period is predictable by the combined meal, physical, HR, and HRV data.

Slow glucose dynamics is associated with large postprandial glucose spikes

The overall data modeling strategy is shown in Figures 2A–2C, where we first focus on ingestion events, glucose, and circadian rhythms (model 1, Figure 2A), then the relationships between the physical and the heart activity signals (model 2, Figure 2B), before finally adding interactions from the physical activity and heart signals to the glucose levels (model 3, Figure 2C). Based on the visual exploration (Figure 1) and physiological knowledge, we first built a minimal dynamical model of glucose levels (model 1) that included the following four features: (1) the ability to produce a continuous postprandial glucose response following an ingestion event, (2) negative feedback (representing the regulating action of insulin, depicted as a feedback loop in Figure 2A), (3) a random component that captures the glucose fluctuations between ingestion events and overnight, and (4) a circadian baseline rhythm (discussed in the next section).

These features were modeled with a system of stochastic differential equations (SDEs) (see STAR Methods), where ingestion events act to perturb glucose to higher levels. We refer to the increase in glucose levels relative to the baseline level caused by meals as the “meal height” (Figure 2D). As meals can cause different glucose responses according to their content, we allow each meal with a unique text entry to have a separate meal height parameter and report the mean across all meals for each participant. After ingestion causes a glucose increase, glucose levels return to their steady-state values (reflecting homeostasis). The decay kinetics and precise shape of the response will depend on the parameters of the model (Figure 2D), which are learned for each participant. Specifically, this individual-specific response to a meal perturbation can be summarized with three parameters: a half-life reflecting the time taken for glucose to return to baseline levels, the mean increase in glucose levels caused by meal consumption (referred to as the mean meal height), and a damping coefficient specifying whether the response profile is akin to an overdamped (a rapid glucose increase followed by a monotonous slower decay, i.e., non-dipping) or an underdamped (leading to a slower initial increase followed by decay and overshoot, i.e., dipping) response. To account for noisy fluctuations in the data, the glucose dynamics is also subjected to random perturbations in the corresponding SDE, meaning that the glucose time series data can show noisy deviations from the idealized meal response.

For each participant, the entire glucose time series is probabilistically matched (using exact likelihood calculations) to the model using a Gaussian state space model (a.k.a. a Kalman

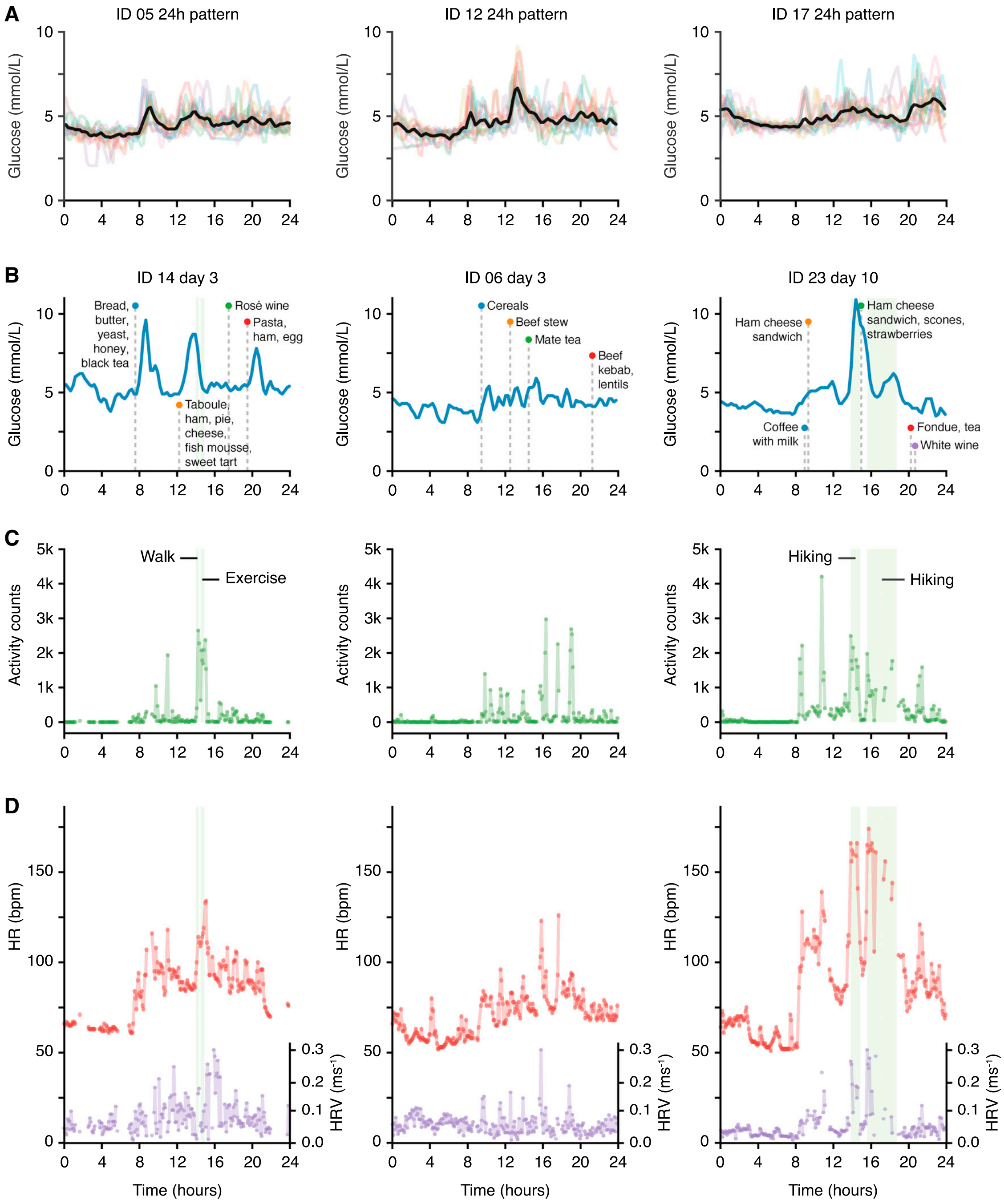

Figure 1. Exploratory analysis of wearable signals: examples of 24-h trends and responses to external stressors

(A) Continuous glucose monitoring (CGM) data: superposition of all recorded days of data shown on the same 24-h scale for three different participants (see related Figure S7 for all participants). Black, average over all days; colored lines, data for individual days; time axis, wall clock time.

(B) Selected day examples of CGM glucose levels alongside recorded ingestion events for three participants (same individuals shown in B–D). Blue, glucose levels; green shade, recorded activity events; time axis, wall clock time; vertical dashed lines, ingestion events.

(C) Selected day examples of physical activity measured with the CamNtech Actiheart device. Green, physical activity; green shade, recorded activity events; time axis, clock time.

(D) Selected day examples of HR and HRV measured with the CamNtech Actiheart device. Green shade, recorded activity events; purple, heart rate variability (HRV) (quantified with $RMSSD^{-1}$ in ms^{-1}); red, heart rate (HR) in beats per minute (bpm); time axis, wall clock time.

See also Figures S2–S7 for visualization of all data for all participants.

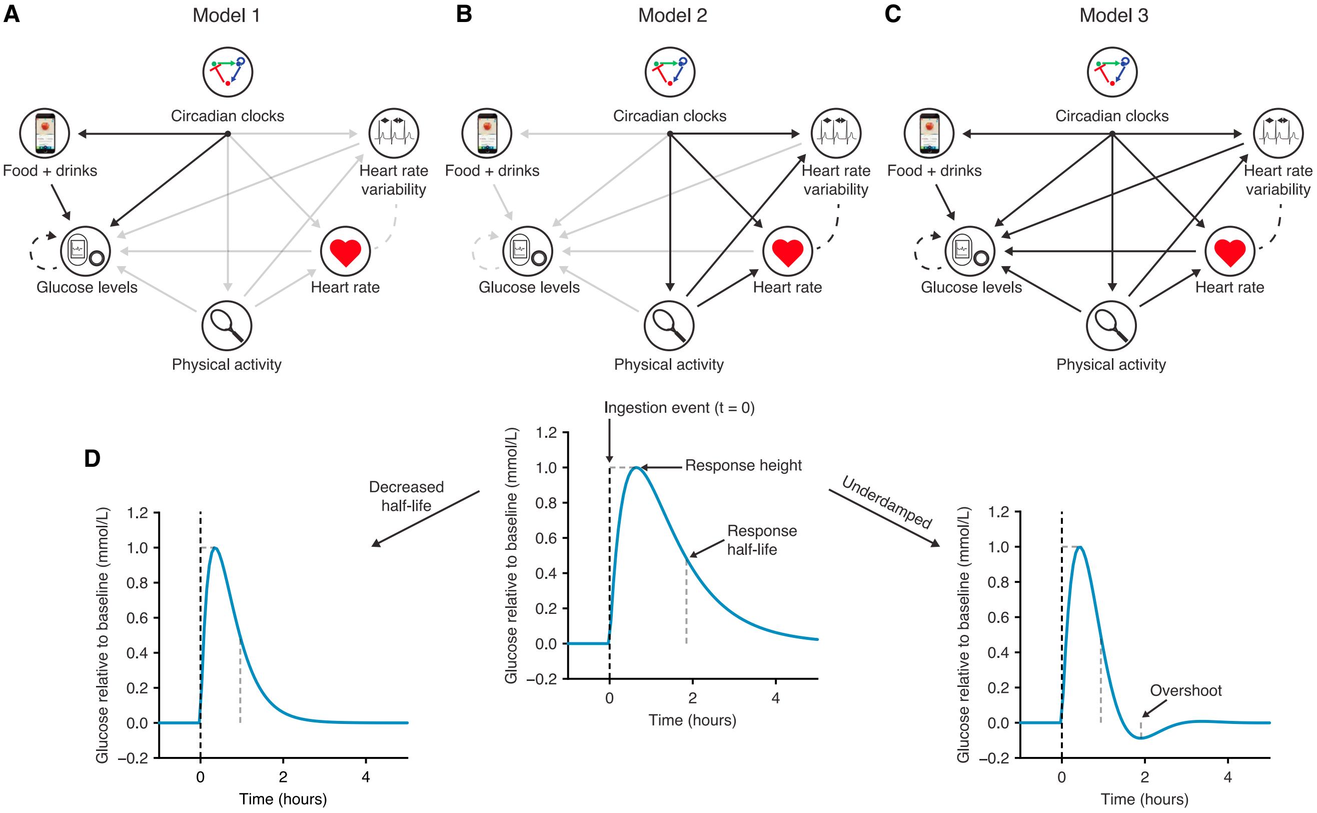

Figure 2. Schematic showing the three different models and parameter interpretation

(A–C) The glucose and ingestion events interaction model (A, model 1), the physical and heart activity interaction model (B, model 2), and the full model (C, model 3). Solid arrows represent direct unidirectional influences, while dashed lines represent correlated fluctuations that are not specifically directional.

(D) A meal or drink event causes a glucose increase to a specific meal height relative to the baseline glucose value. The response half-life determines how quickly glucose returns to baseline. Underdamping (defined as a negative damping coefficient) leads to an overshoot below the baseline values.

filter), and we infer each of the model parameters using Markov chain Monte Carlo (MCMC) sampling within a Bayesian framework that yields uncertainty estimates for each parameter (STAR Methods and supplemental information).

We first verified model performance by assessing the correlation coefficient between the fitted meal response function and the data (Figure S8). The correlation coefficient generally ranged from 0.5 to 0.8 but was particularly low for participant ID 04. Visual inspection of this participant's raw data showed large glucose spikes following physical activity (Figure S9) and hence were not explained in this initial model, which we address below with more complex modeling. While all model parameters are shown in Figure S8, we here focus on three summary metrics of the glucose dynamics.

Response half-lives ranged from 1 to 2.2 h (Figure 3A), thus showing a dynamic range of 220%. The mean meal response heights ranged from 0.5 to 1.5 mmol/L above baseline (Figure 3B), and a more detailed examination revealed that glucose responses for a given individual vary according to the specific item consumed (Figure S9). The posterior parameter distributions for each participant (Figures 3A and 3B) quantify the uncertainty associated with the parameter estimates for each participant; in some cases the distributions were overlapping between two individuals, while in other cases the distributions were clearly separated (e.g., half-life comparing IDs 20 and 23, Figure 3A).[47] Comparing parameter values across participants, we found a positive relationship between response half-lives and mean meal heights, with slower glucose response half-lives associated with larger postprandial glucose spikes ($R = 0.44$, $p = 0.02$, Figure 3C). This suggested that postprandial glucose control (i.e., the height of glucose spikes following meals) depends on glucose clearance time, which might be determined physiologically by insulin sensitivity or β cell function (discussion).

The damping coefficients describing the shapes of glucose responses were clustered around 0 across all participants (Figure S8), where values of 0 represent "critical" damping at the border between overdamped (non-dipping profiles, damping coefficient >0) and underdamped (profiles with a dip, damping coefficient <0). Interestingly, glucose responses were proposed to be critically damped in an early glucose model,[48] which would be consistent with our finding that the inferred values are scattered around 0. However, we clearly find individual-specific response profiles, with participant ID 14 showing a distinct underdamped glucose response compared with the critically damped response in ID 23 (Figures 3D–3F). The inferred meal response and circadian time functions (orange) are smoother

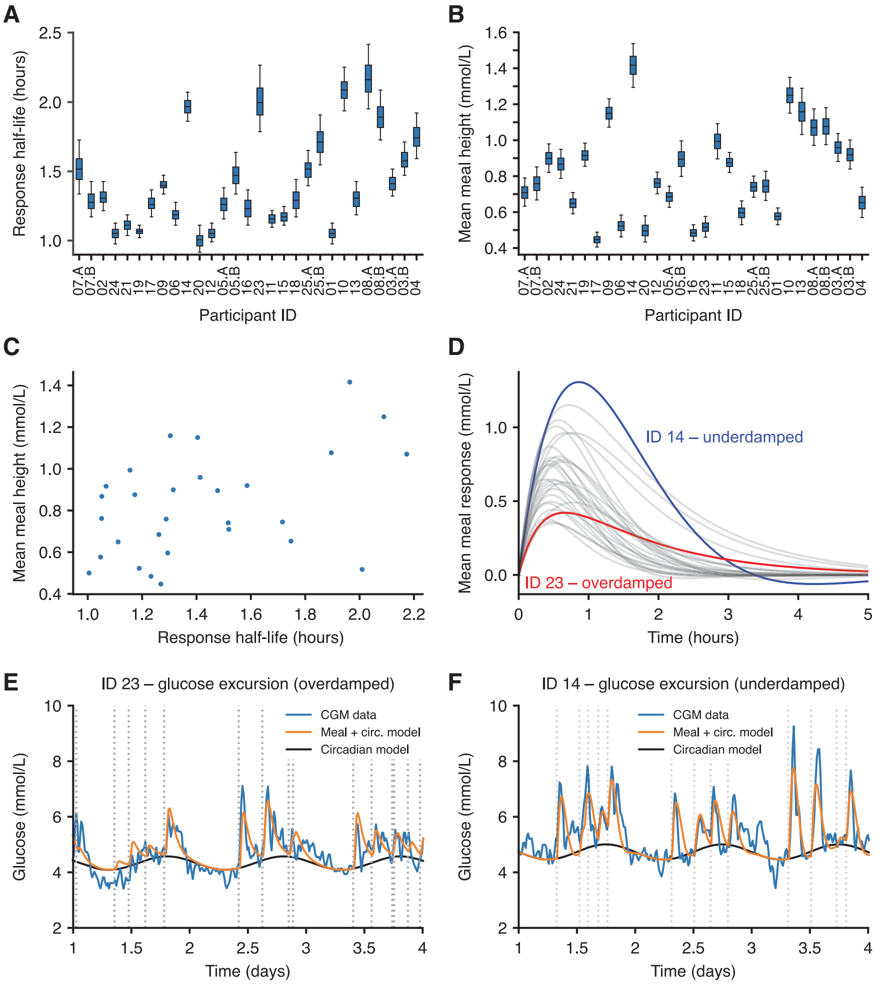

Figure 3. Characterizing participant-specific postmeal glycemic responses

(A) The inferred glucose response half-life for each participant, defined as the model-predicted time it would take for glucose levels to fall to 0.5 mmol/L following a peak of 1 mmol/L. The boxplots represent the 25th, 50th (median), and 75th percentiles of the posterior distribution and the whiskers represent the 5th and 95th percentiles.

(B) The average meal glucose spike height calculated as the mean height over all meals consumed during the experiment.

(C) The average meal height as a function of the glucose meal response half-life. Points represent the mean posterior value for each participant.

(D) Average meal response profiles using the posterior mean parameter values.

(E and F) Examples comparing the CGM data (blue) with the model prediction incorporating circadian dynamics (black) plus meal consumption (orange) for two participants with overdamped and underdamped dynamics, respectively. The time stamps of meals are shown as dashed lines. Participant order is the same in Figures 3A, 3B, 4A, and 4B.

See also Figures S8 and S9.

than the glucose data (blue, Figures 3E and 3F), but the full model that also adds random fluctuations produces glucose traces that closely resemble the glucose data (Figure S9).

The measured glucose CV, a metric of glycemic control used in clinical settings,[10,11] showed significant associations with both the response half-lives (linear regression p = 0.03) and the average meal heights (p = 0.01, linear regression model R^2 using both variables = 0.63). While the damping coefficient was not significantly associated with glucose CV, the individual shapes of glucose responses might play a role in other aspects of glucose dynamics such as overshooting and hunger.[49] Our results highlight that glucose response half-lives play a role in glycemic control and may be a relevant metric for both fundamental research and clinical purposes.

Circadian rhythms in baseline glucose levels are individual-specific

In addition to the input from ingestion events, the model also allows for an underlying circadian rhythm in glucose levels described with three parameters: a baseline level that specifies the glucose at the trough of the oscillation, an amplitude parameter denoting the difference between the trough and the peak of the oscillation, and the peak time of the oscillation. These circadian parameters are inferred for each individual jointly with the meal response parameters when fitting model 1 to the glucose data using MCMC (STAR Methods).

The amplitudes of underlying circadian glucose rhythms were participant specific (Figure 4A), being virtually null for some individuals, while exceeding 1 mmol/L for others (Figure 4C, IDs 03 and 07). Notably, the parameter uncertainty was small enough that there was no overlap in the estimates for IDs 03 and 07 (Figure 4A). To identify subjects whose profiles do not support a circadian baseline trend, we fitted an alternative model that lacked a circadian baseline and compared the two models using the Bayesian information criterion (BIC). For IDs 10, 13, 08, 25, 03, and 04 (which have the weakest amplitude according to Figure 4A), the BIC indicated evidence for the model lacking the circadian baseline, while the BIC favored the model with an additional circadian component for all remaining participants (Figure S10). The combination of the amplitude posterior estimates and heterogeneous model preference according to BIC thus suggests that circadian baseline glucose oscillations are individual-specific physiological characteristics.

The peak times of the glucose circadian oscillations similarly varied between participants (Figure 4B, with the same participant order as in Figure 4A), with peak times of the circadian baselines typically falling around the mid-afternoon, but with significant shifts in some individuals. For example, participant ID 20 had a peak time at 10:00, while it occurred much later for

CellPress
OPEN ACCESS

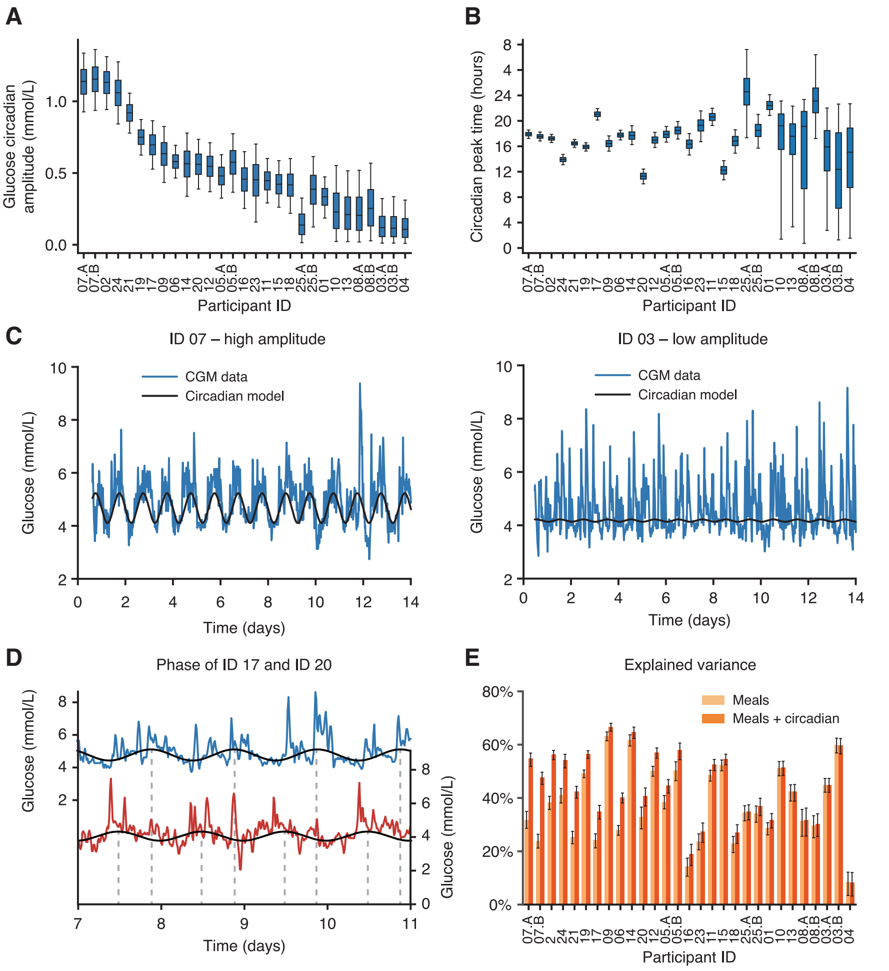

Figure 4. Amplitudes and peak times of circadian baseline levels of glucose are highly heterogeneous between participants

(A) The amplitude of the 24-h sinusoidal circadian rhythm in baseline glucose levels after model fitting to the CGM data for all participants. The boxes represent the 25th, 50th (median), and 75th percentiles of the posterior distribution and the whiskers represent the 5th and 95th percentiles.

(B) The circadian peak time of the glucose circadian rhythm across all participants.

(C) Examples of participants with a high (ID 07) and a low (ID 03) amplitude glucose circadian rhythm. Blue, CGM data; black, fitted model of circadian baseline (using mean posterior parameter values).

(D) Examples showing two participants with large phase difference in underlying glucose rhythm (ID 20 peak phase 10:00, ID 17 peak phase 20:00).

(E) The explained variance in glucose levels using just the meal component of the model (light orange) compared with the inclusion of the circadian rhythm (dark orange). Error bars represent the 5th and 95th percentiles of the posterior distribution. Participant order is the same in Figures 3A, 3B, 4A, and 4B.

See also Figure S10.

participant ID 17, falling at 20:00 (Figure 4D). The peak time distributions showed tight confidence intervals for participants with large amplitudes and wide intervals for participants with weaker amplitudes (Figure 4B). This relationship is probably caused by a lower signal-to-noise ratio for participants with a low circadian amplitude.

Overall, the underlying circadian glucose rhythm can explain >15% of glycemic variability in addition to the meal model for participants with large amplitudes (Figure 4E). While we have not tested whether it would be possible to modify either the peak time or the amplitude of this rhythm, these personalized parameters should prove to be useful in applications such as personalized meal timing (discussion).

HR is well predicted by physical activity and time of day, but the predictability of HRV varies between individuals

We next focused on the physical activity, HR, and HRV data, where we aimed to model the dependencies between the variables and quantify the ability of subsets of the three signals to explain the variance of others, in addition to the contribution of circadian oscillations. For this, we created a new model (model 2, Figure 2B) that incorporated the influence of physical activity on HR and HRV, and we used MCMC to sample from model parameters and quantify differences between individuals (all parameters shown in Figure S11).

For HR, the combination of a circadian trendline and physical activity as two inputs was consistently predictive, explaining 40%–65% of HR variance across all participants (Figure 5A). Figures 5C and 5D show an example of the predicted HR (orange) for two different participants using the underlying circadian trend (black) and integrating the physical activity (green). While the circadian contribution to the explained HR variance differs for these two participants (Figure 5A), the correlation between the predicted and the observed HR was ~0.8 for both participants, demonstrating that time of day and activity state are necessary for optimal personalized modeling of HR, which is consistent with previous studies.[50]

The predictability of HRV was, in contrast, much more heterogeneous between participants, with total variance explained between 20% and 80% (Figure 5B). This notable difference is illustrated with two participants, showing a favorable prediction for participant ID 25 (R = 0.89, Figure 5F) compared with ID 14 (R = 0.44, Figure 5E). In addition to inputs from physical activity and the circadian trend, we evaluated whether the correlations between HR and HRV could be exploited by using HR to predict HRV (which is technically more difficult to measure than HR). The dependence between HR and HRV showed marked interindividual differences, where for ID 25 the HR signal explained 40% of the variance compared with using only activity and circadian trend, but for ID 14 the addition of HR made no difference in HRV prediction (Figures 5E, 5F, and S11). Given that HR and

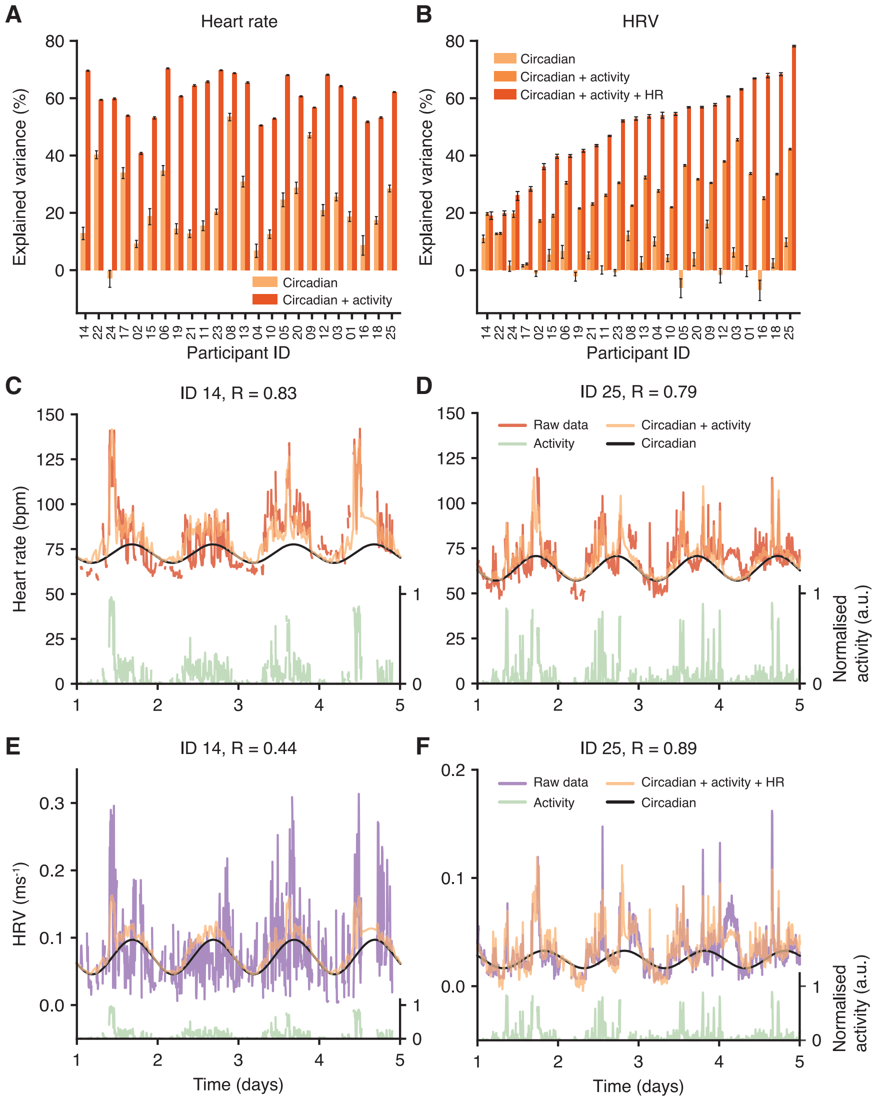

Figure 5. HRV predictions using multisignal inputs and circadian rhythms are more heterogeneous than for HR

(A) The amount of variance of the HR signal explained by circadian rhythms (light orange) and a combined model with circadian rhythms and physical activity (dark orange). Error bars represent the 5th and 95th percentiles of the posterior distribution.

(B) The amount of variance of the HRV ($RMSSD^{-1}$) signal explained by circadian rhythms (light orange), a combined model with circadian rhythms and physical activity (medium orange), and a combined model with circadian rhythms, physical activity, and HR (dark orange).

(C and D) Examples comparing HR data with model predictions for two participants. Red, HR data; black, baseline circadian rhythm; green, physical activity (shown on normalized scale where 1 represents the maximum value); orange, model prediction with circadian rhythm and integrating activity.

(E and F) Examples comparing HRV data with model predictions for two participants. Purple, HRV data; black, baseline circadian rhythm; green, physical activity (shown on normalized scale where 1 represents the maximum value); orange, model prediction using circadian rhythms, physical activity, and HR. Participant order is the same in (A) and (B).

See also Figure S11.

HRV receive different inputs from the SNS and PNS,[25] the strength of this dependence may be a function of the autonomic nervous system. Of note, ID 14 was previously diagnosed with diabetes (currently treated only with lifestyle measures and not pharmacological treatment), and autonomic dysfunction is a known complication of diabetes.[51]

Integrating physical and heart activity signals helps explain glycemic dynamics

As a final modeling step, we integrated the physical and heart activity signals with the glucose-ingestion model to quantify how much of the glucose dynamics can be accounted for with physical activity, HR, and HRV (model 3, Figure 2C). To simplify the model inference, the parameters describing the physical and heart activity model in isolation (model 2, Figure 2B) were locked to their posterior mean values, and we added three new parameters describing the input of physical activity, HR, and HRV on glucose levels, respectively (model 3, Figure 2C). These influences were left unconstrained and could have positive, negative, or zero effect on glucose levels.

Model fitting revealed that the effect of physical activity accelerometer counts on glucose (parameter $C_{5,1}$) was generally negative, the effect of HR (parameter $C_{5,2}$) was generally positive, and the effect of HRV (parameter $C_{5,3}$) was typically neutral across all participants (Figures 6A–6C). Given that we observed increased glucose levels during some periods of exercise during data visualization (Figure 1B), the negative influence of physical activity accelerometer counts on glucose as revealed by the model parameter $C_{5,1}$ (Figure 6A) was not expected. To test the robustness of this prediction, we therefore re-fitted the data using three simpler models, where there was only one input at a time from the physical and heart activity signals (Figure S12). The influence of physical activity on glucose remained negative, even when it was the sole input from model 2 into glucose levels, further suggesting that the overall dominating effect of physical activity is to deplete glucose levels among the participants of our study. Meanwhile, as HR acts to increase glucose levels ($C_{5,2}$), increased HR during intense exercise can still lead to a net increase in predicted glucose levels.

We next quantified the importance of heart and physical activity for glucose predictions by comparing the variance explained with meals and circadian rhythms compared with all

CellPress
OPEN ACCESS

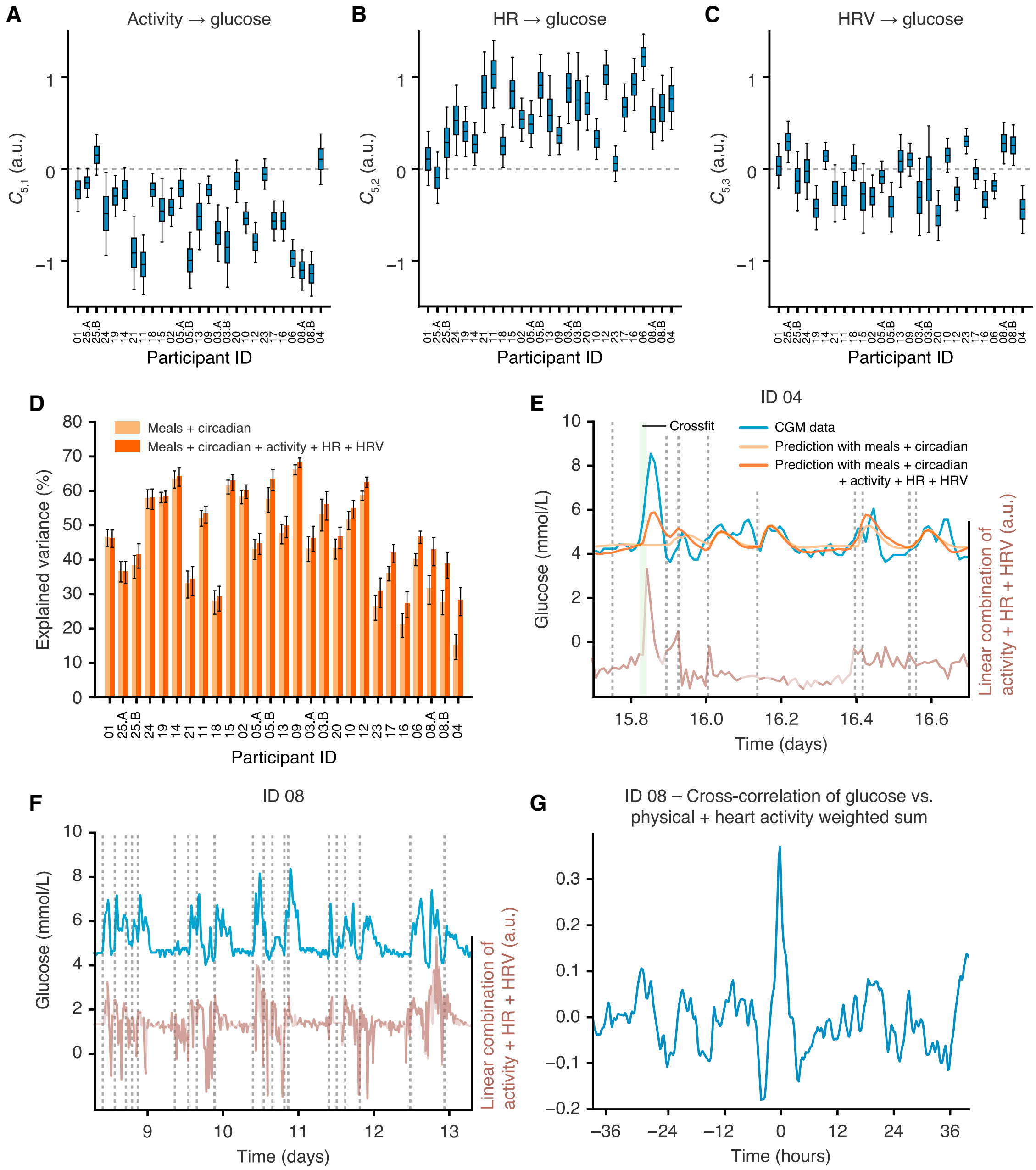

Figure 6. Adding physical activity, HR, and HRV into the glucose dynamics model can help explain glucose dynamics

(A–C) Posterior distributions (shown as boxplots) of the model coefficients $C_{5,1}$, $C_{5,2}$, and $C_{5,3}$ across all participants, which correspond to the influence on glucose of physical activity, HR, and HRV, respectively. The boxplots represent the 25th, 50th (median), and 75th percentiles of the posterior distribution and the whiskers represent the 5th and 95th percentiles.

(D) A comparison of the variance explained in the glucose signal using just the meal and circadian rhythm model (light orange) compared with the prediction that also incorporates physical activity, HR, and HRV (dark orange). Error bars represent the 5th and 95th percentiles of the posterior distribution.

(E) Example from ID 04 shows that the physical and heart activity data partially explain an exercise-induced glucose spike. Blue, glucose data; light orange, prediction using meal and circadian model components; dark orange, prediction including meal and circadian model components, physical activity, HR, and HRV; green shade, recorded activity events; brown, the weighted sum of the physical activity, HR, and HRV variables according to the inferred coefficients $C_{5,1}$, $C_{5,2}$, and $C_{5,3}$; vertical dashed lines, ingestion events.

(F) Example from ID 08 showing how glucose dynamics (blue) track with the weighted sum of the physical activity, HR, and HRV variables according to the inferred coefficients $C_{5,1}$, $C_{5,2}$, and $C_{5,3}$ (dark brown, data only; light brown, using filtered estimations from model 2 to fill missing physical and heart activity data; vertical dashed lines, ingestion events).

(G) The cross-correlation of the glucose (blue in F) with the weighted sum of the physical activity, HR, and HRV variables (brown in F) using all recorded data.

Participant order is the same between (A), (B), and (C).

See also Figure S12.

components, including physical activity, HR, and HRV (Figure 6D). Overall, the amount of additional explained variance by the physical and heart activity signals was modest for most participants but contributed up to 15% in some individuals (Figure 6D).

Among the participants whose glucose dynamics benefit most from the physical and heart activity signals, the mode of action also differed. For participant ID 04, where the prediction of glucose was the lowest with model 1 (Figure 4E), the inclusion of physical and heart activity signals allowed for partial prediction of exercise-induced glucose spikes that were otherwise absent from the glucose model that contained meals only (Figure 6E). For participant ID 08, there were no notable isolated glucose spikes that could be predicted by the physical and heart activity data (Figure 6F). Instead, the linear combination of the physical and heart activity variables (weighted according to the inferred coefficients $C_{5,1}$–$C_{5,3}$) appeared to track the glucose levels (Figure 6F), and the cross-correlation profile showed a maximum correlation between the two signals without time delay (Figure 6G). This suggests that for this mode of action, the physical and heart activity signals contribute diffusely to capture glucose baseline trends spread over the time series.

DISCUSSION

This study makes two contributions to the quantitative analysis of glucose dynamics in terms of study design and computational analysis. First, we used several wearable biosensors to measure multiple, interconnected data streams simultaneously. Inferring dynamical models would not be possible without such high-resolution time series. On a practical level, the main advantages of health measurements with wearable biosensors are their efficiency, non-invasiveness, and relative simplicity: in our study it took a team of two researchers 2 days to complete questionnaires and set up devices to record data for all 25 participants simultaneously. Second, we developed a data analysis method that combines stochastic dynamical modeling with Bayesian inference to learn personal parameters along with their associated uncertainty, and this parameter uncertainty was necessary to compare participants. The main insight of such personalized modeling is the possibility to robustly quantify individualized response patterns across a range of parameters and metrics, from the glucose half-lives and circadian oscillations, the coupling between HR and HRV, to the benefit of adding additional signals to predicting glucose.

The output of this modeling has wide implications for understanding both the biological underpinnings of cardiometabolic dysfunction as well as consequences for the use of wearables in a clinical setting. We first started by combining the food and drink events with the glucose time series, where we found that slow glucose dynamics are associated with large postprandial glucose spikes. Mechanistically, slow glucose disposal could relate to the quantity of ingested carbohydrates, the rate of gut absorption,[52] its metabolism,[53] the suppression of endogenous glucose production,[54] insulin resistance, or β cell function.[2]

We also detected highly personalized circadian rhythms in the baseline glucose levels by quantifying amplitude and peak-time parameters in each individual along with uncertainty estimates (Figures 4A and 4B). While diurnal rhythms in β cell function and insulin sensitivity have previously been shown within healthy populations,[55–57] it was unexpected to find significant differences in the amplitudes and phases of circadian oscillations in baseline glucose levels between healthy individuals. The extent to which these 24-h rhythms are determined by central or peripheral clocks or whether they are largely entrained by meal timing[58] cannot be deduced without further information. Future studies will determine whether this circadian glucose baseline trend is predictive of responses to specific meal times, e.g., in time-restricted eating, an intervention that restricts eating to a specific window within the 24-h clock.[59]

Our results also have practical implications for clinicians as the physical and heart activity data explained up to 15% of glucose variability in our study, although this was highly variable between participants. From a clinical perspective, the incorporation of these additional physical and heart activity signals for some individuals might help both patients and clinicians understand glucose dynamics that seem otherwise disconnected from meal consumption (e.g., Figure 6E). As outlined in the motivation paragraph, clinicians and diabetic patients can link glucose excursion with ingestion events and intensive physical activity but find it challenging to do so for the remaining glucose dynamics observed throughout the day.

There are multiple possible approaches to modeling multimodal data such as we collected, and the particular structure of the glucose model has often been dictated by the data available and by the stated goal.[34–36] Models based on differential equations range from simple, minimal models[37] to mechanistically detailed descriptions that include more variables, more spatial compartments, and dozens of additional parameters.[38] Inspired by these more complex models, there are many possible extensions that could be added to our glucose model, such as glucose absorption rates of mixed meals due to food content in carbohydrates, but also fat, fiber, and protein contents, which are known to slow down nutrient absorption,[14] although the addition of meal-specific response shapes would effectively double the number of meal-related parameters. In addition to differential equation models, time series methods such as wavelets can also be applied to multimodal continuous wearable signals to detect, for example, changes in period and amplitude over time, to identify transient events, to assess associations between signals, and to perform signal denoising.[60–62] Explicit models and time series analysis methods are ultimately complementary tools that come with their own sets of advantages and limitations.

An area that has seen a broad spectrum of time series models concerns short-term glucose forecasting, typically for applications in closed-loop insulin delivery systems.[33,44] Gaussian state-space models that are conceptually similar to ours but with more variables have been deployed in artificial pancreas devices.[63,64] Machine learning methods, including support vector machines (SVMs) and neural networks, have also been used for short-range forecasting,[39,40] where the advantage of such methods is that more complex non-linear dependencies and long-range memory can be captured. As our focus was on explaining the total time series rather than short-term forecasting, we here traded some of this flexibility for explainability by

assuming a relatively simple dynamical model with interpretable components and parameters. In the future, we envisage several applications, such as larger-scale epidemiological studies (e.g., do inferred parameters track with health state?) and clinical trials to see whether parameters change in response to an intervention. Particularly for clinical trials, point estimation of parameters is not adequate, and uncertainty estimates are required to perform statistical tests for a given individual, which we achieve here through MCMC. In health care, there is increasing interest in digital twins[65,66] to integrate multiple clinical data streams, devise personalized treatments, and perform risk modeling. As our approach contains interpretable parameters, it lends itself readily to simulating hypothetical situations (e.g., by altering circadian amplitude or glucose response time). Overall, our method transforms a multivariable wearable data input into a series of metrics that describe the dependencies between physiological variables, including the relaxation timescales after external perturbations and circadian properties, and this approach provides a platform for probing physiological changes across circadian perturbations, aging, and cardiometabolic disorders.

Limitations of the study

Our study population was young and in good health overall, and we lack additional, more detailed health information or standard clinical metrics such as glycated hemoglobin (HbA1c). Since we opted here for a small-scale study with a focus on the methodology and models to combine multiple wearable sensors, we have sought to identify differences between individuals without attempting to associate them with either good or bad health outcomes. Future studies that use larger and more diverse cohorts could use the proposed method to relate the inferred personal glycemic parameters to lifestyle, environmental, or genetic factors, and it would also be useful to explore whether candidates that would benefit from inclusion of physical and heart activity to glucose modeling can be predicted in advance.

With respect to the modeling, a potential limitation of our approach is the use of a linear differential equation model, which may not be able to capture more complex phenomena such as eventual decreases in hepatic glucose production during prolonged exercise.[67] A recent study based on deep learning found that the addition of wristband activity data improved the root-mean-square error of 60-min glucose forecasting by 2.25 mg/dL (0.1 mmol/L) from a baseline of 35.3 mg/dL (2.0 mmol/L), and hence, more substantial improvements in glucose predictions may prove to be a difficult challenge even with more flexible models.[68]

STAR★METHODS

Detailed methods are provided in the online version of this paper and include the following:

- KEY RESOURCES TABLE
- RESOURCE AVAILABILITY
  - Lead contact
  - Materials availability
  - Data and code availability
- EXPERIMENTAL MODEL AND STUDY PARTICIPANT DETAILS
- METHOD DETAILS
  - Devices and experimental design
  - Pre-processing CGM data
  - Data analysis
- QUANTIFICATION AND STATISTICAL ANALYSIS

SUPPLEMENTAL INFORMATION

Supplemental information can be found online at https://doi.org/10.1016/j.crmeth.2023.100545.

ACKNOWLEDGMENTS

The authors wish to thank all participants in the study and the team of Professor Satchidananda Panda (Salk Institute) for the use of the myCircadianClock smartphone application. This project was supported by grant 2018-427 of the Strategic Focal Area “Personalized Health and Related Technologies (PHRT)” of the ETH Domain to N.E.P. T.-H.C.’s research is supported by the Leenaards Foundation, the Vontobel Foundation, the Nutrition 2000plus Foundation, the SwissLife Jubiläumsstiftung Foundation, the Swiss Society of Endocrinology and Diabetes, and the Swiss Multiple Sclerosis Society. The work in the Naef lab and purchase of the wearable devices was supported by the EPFL.

AUTHOR CONTRIBUTIONS

Conceptualization, N.E.P., T.-H.C., and F.N.; methodology, N.E.P., T.-H.C., and F.N.; data collection, N.E.P. and T.-H.C.; computation and data analysis, N.E.P.; data visualization, N.E.P., T.-H.C., and F.N.; writing – original draft preparation, N.E.P.; writing – review & editing, N.E.P., T.-H.C., and F.N.; project administration, N.E.P., T.-H.C., and F.N.; supervision, T.-H.C. and F.N.; funding acquisition, N.E.P., T.-H.C., and F.N. All authors have read and agreed to the published version of the manuscript.

DECLARATION OF INTERESTS

The authors declare no competing interests.

INCLUSION AND DIVERSITY

We support inclusive, diverse, and equitable conduct of research.

Received: December 21, 2022
Revised: April 13, 2023
Accepted: July 6, 2023
Published: July 31, 2023

REFERENCES

1. Trachana, K., Bargaje, R., Glusman, G., Price, N.D., Huang, S., and Hood, L.E. (2018). Taking systems medicine to heart. Circ. Res. *122*, 1276–1289. https://doi.org/10.1161/CIRCRESAHA.117.310999.
2. Kahn, S.E., Cooper, M.E., and Del Prato, S. (2014). Pathophysiology and treatment of type 2 diabetes: Perspectives on the past, present, and future. Lancet *383*, 1068–1083. https://doi.org/10.1016/S0140-6736(13)62154-6.
3. Tirone, T.A., and Brunicardi, F.C. (2001). Overview of glucose regulation. World J. Surg. *25*, 461–467. https://doi.org/10.1007/s002680020338.
4. Forbes, J.M., and Fotheringham, A.K. (2017). Vascular complications in diabetes: old messages, new thoughts. Diabetologia *60*, 2129–2138. https://doi.org/10.1007/s00125-017-4360-x.
5. Coutinho, M., Gerstein, H.C., Wang, Y., and Yusuf, S. (1999). The relationship between glucose and incident cardiovascular events. Diabetes Care *22*, 233–240. https://doi.org/10.2337/diacare.22.2.233.

6. Emerging Risk Factors Collaboration; Sarwar, N., Gao, P., Seshasai, S.R.K., Gobin, R., Kaptoge, S., Di Angelantonio, E., Ingelsson, E., Lawlor, D.A., Selvin, E., et al. (2010). Diabetes mellitus, fasting blood glucose concentration, and risk of vascular disease: A collaborative meta-analysis of 102 prospective studies. Lancet *375*, 2215–2222. https://doi.org/10.1016/S0140-6736(10)60484-9.

7. Selvin, E., Steffes, M.W., Zhu, H., Matsushita, K., Wagenknecht, L., Pankow, J., Coresh, J., and Brancati, F.L. (2010). Glycated Hemoglobin, Diabetes, and Cardiovascular Risk in Nondiabetic Adults. N. Engl. J. Med. *362*, 800–811. https://doi.org/10.1056/nejmoa0908359.

8. Agarwal, S., Mathew, J., Davis, G.M., Shephardson, A., Levine, A., Louard, R., Urrutia, A., Perez-Guzman, C., Umpierrez, G.E., Peng, L., and Pasquel, F.J. (2021). Continuous Glucose Monitoring in the Intensive Care Unit During the COVID-19 Pandemic. Diabetes Care *44*, 847–849. https://doi.org/10.2337/dc20-2219.

9. Hansen, E.A., Klee, P., Dirlewanger, M., Bouthors, T., Elowe-Gruau, E., Stoppa-Vaucher, S., Phan-Hug, F., Antoniou, M.C., Pasquier, J., Dwyer, A.A., et al. (2018). Accuracy, satisfaction and usability of a flash glucose monitoring system among children and adolescents with type 1 diabetes attending a summer camp. Pediatr. Diabetes *19*, 1276–1284. https://doi.org/10.1111/pedi.12723.

10. Battelino, T., Danne, T., Bergenstal, R.M., Amiel, S.A., Beck, R., Biester, T., Bosi, E., Buckingham, B.A., Cefalu, W.T., Close, K.L., et al. (2019). Clinical targets for continuous glucose monitoring data interpretation: Recommendations from the international consensus on time in range. Diabetes Care *42*, 1593–1603. https://doi.org/10.2337/dci19-0028.

11. Ceriello, A., Monnier, L., and Owens, D. (2019). Glycaemic variability in diabetes: clinical and therapeutic implications. Lancet Diabetes Endocrinol. *7*, 221–230. https://doi.org/10.1016/S2213-8587(18)30136-0.

12. Danne, T., Nimri, R., Battelino, T., Bergenstal, R.M., Close, K.L., DeVries, J.H., Garg, S., Heinemann, L., Hirsch, I., Amiel, S.A., et al. (2017). International consensus on use of continuous glucose monitoring. Diabetes Care *40*, 1631–1640. https://doi.org/10.2337/dc17-1600.

13. Monnier, L., Colette, C., Wojtusciszyn, A., Dejager, S., Renard, E., Molinari, N., and Owens, D.R. (2017). Toward defining the threshold between low and high glucose variability in diabetes. Diabetes Care *40*, 832–838. https://doi.org/10.2337/dc16-1769.

14. Berry, S.E., Valdes, A.M., Drew, D.A., Asnicar, F., Mazidi, M., Wolf, J., Capdevila, J., Hadjigeorgiou, G., Davies, R., Al Khatib, H., et al. (2020). Human postprandial responses to food and potential for precision nutrition. Nat. Med. *26*, 964–973. https://doi.org/10.1038/s41591-020-0934-0.

15. Korem, T., Zeevi, D., Zmora, N., Weissbrod, O., Bar, N., Lotan-Pompan, M., Avnit-Sagi, T., Kosower, N., Malka, G., Rein, M., et al. (2017). Bread Affects Clinical Parameters and Induces Gut Microbiome-Associated Personal Glycemic Responses. Cell Metabol. *25*, 1243–1253.e5. https://doi.org/10.1016/j.cmet.2017.05.002.

16. Zeevi, D., Korem, T., Zmora, N., Israeli, D., Rothschild, D., Weinberger, A., Ben-Yacov, O., Lador, D., Avnit-Sagi, T., Lotan-Pompan, M., et al. (2015). Personalized Nutrition by Prediction of Glycemic Responses. Cell *163*, 1079–1094. https://doi.org/10.1016/j.cell.2015.11.001.

17. Flanagan, A., Bechtold, D.A., Pot, G.K., and Johnston, J.D. (2021). Chrono-nutrition: From molecular and neuronal mechanisms to human epidemiology and timed feeding patterns. J. Neurochem. *157*, 53–72. https://doi.org/10.1111/jnc.15246.

18. Levi, F., and Schibler, U. (2007). Circadian Rhythms: Mechanisms and Therapeutic Implications. Annu. Rev. Pharmacol. Toxicol. *47*, 593–628. https://doi.org/10.1146/annurev.pharmtox.47.120505.105208.

19. Qian, J., and Scheer, F.A.J.L. (2016). Circadian System and Glucose Metabolism: Implications for Physiology and Disease. Trends Endocrinol. Metabol. *27*, 282–293. https://doi.org/10.1016/j.tem.2016.03.005.

20. Van Cauter, E., Polonsky, K.S., and Scheen, A.J. (1997). Roles of circadian rhythmicity and sleep in human glucose regulation. Endocr. Rev. *18*, 716–738. https://doi.org/10.1210/er.18.5.716.

21. Aparicio, N.J., Puchulu, F.E., Gagliardino, J.J., Ruiz, M., Llorens, J.M., Ruiz, J., Lamas, A., and De Miguel, R. (1974). Circadian variation of the blood glucose, plasma insulin and human growth hormone levels in response to an oral glucose load in normal subjects. Diabetes *23*, 132–137. https://doi.org/10.2337/diab.23.2.132.

22. Porcellati, F., Lucidi, P., Bolli, G.B., and Fanelli, C.G. (2013). Thirty Years of Research on the Dawn Phenomenon: Lessons to Optimize Blood Glucose Control in Diabetes. Diabetes Care *36*, 3860–3862. https://doi.org/10.2337/dc13-2088.

23. Schmidt, M.I., Hadji-Georgopoulos, A., Rendell, M., Margolis, S., and Kowarski, A. (1981). The dawn phenomenon, an early morning glucose rise: Implications for diabetic intraday blood glucose variation. Diabetes Care *4*, 579–585. https://doi.org/10.2337/diacare.4.6.579.

24. Johnson, M.L., Martens, T.W., Criego, A.B., Carlson, A.L., Simonson, G.D., and Bergenstal, R.M. (2019). Utilizing the Ambulatory Glucose Profile to Standardize and Implement Continuous Glucose Monitoring in Clinical Practice. Diabetes Technol. Therapeut. *21*, S217–S225. https://doi.org/10.1089/dia.2019.0034.

25. Shaffer, F., and Ginsberg, J.P. (2017). An Overview of Heart Rate Variability Metrics and Norms. Front. Public Health *5*, 258–317. https://doi.org/10.3389/fpubh.2017.00258.

26. Singh, J.P., Larson, M.G., O'Donnell, C.J., Wilson, P.F., Tsuji, H., Lloyd-Jones, D.M., and Levy, D. (2000). Association of hyperglycemia with reduced heart rate variability (The Framingham Heart Study). Am. J. Cardiol. *86*, 309–312. https://doi.org/10.1016/S0002-9149(00)00920-6.

27. Wu, J.S., Yang, Y.C., Lin, T.S., Huang, Y.H., Chen, J.J., Lu, F.H., Wu, C.H., and Chang, C.J. (2007). Epidemiological evidence of altered cardiac autonomic function in subjects with impaired glucose tolerance but not isolated impaired fasting glucose. J. Clin. Endocrinol. Metab. *92*, 3885–3889. https://doi.org/10.1210/jc.2006-2175.

28. Ewing, D.J., Campbell, I.W., and Clarke, B.F. (1976). Mortality in diabetic autonomic neuropathy. Lancet *1*, 601–603. https://doi.org/10.1016/S0140-6736(76)90413-X.

29. Wehrwein, E.A., Orer, H.S., and Barman, S.M. (2016). Overview of the Anatomy, Physiology, and Pharmacology of the Autonomic Nervous System. Compr. Physiol. *6*, 1239–1278. https://doi.org/10.1002/CPHY.C150037.

30. Shaffer, F., McCraty, R., and Zerr, C.L. (2014). A healthy heart is not a metronome: an integrative review of the heart's anatomy and heart rate variability. Front. Psychol. *5*, 1040–1119. https://doi.org/10.3389/fpsyg.2014.01040.

31. Riddell, M.C., Gallen, I.W., Smart, C.E., Taplin, C.E., Adolfsson, P., Lumb, A.N., Kowalski, A., Rabasa-Lhoret, R., McCrimmon, R.J., Hume, C., et al. (2017). Exercise management in type 1 diabetes: a consensus statement. Lancet Diabetes Endocrinol. *5*, 377–390. https://doi.org/10.1016/S2213-8587(17)30014-1.

32. Yardley, J.E., and Colberg, S.R. (2017). Update on Management of Type 1 Diabetes and Type 2 Diabetes in Athletes. Curr. Sports Med. Rep. *16*, 38–44. https://doi.org/10.1249/jsr.0000000000000327.

33. Woldaregay, A.Z., Årsand, E., Walderhaug, S., Albers, D., Mamykina, L., Botsis, T., and Hartvigsen, G. (2019). Data-driven modeling and prediction of blood glucose dynamics: Machine learning applications in type 1 diabetes. Artif. Intell. Med. *98*, 109–134. https://doi.org/10.1016/j.artmed.2019.07.007.

34. Cobelli, C., Man, C.D., Sparacino, G., Magni, L., De Nicolao, G., and Kovatchev, B.P. (2009). Diabetes: Models, Signals, and Control. IEEE Rev. Biomed. Eng. *2*, 54–96. https://doi.org/10.1109/RBME.2009.2036073.

35. McGrath, T., Murphy, K.G., and Jones, N.S. (2018). Quantitative approaches to energy and glucose homeostasis: Machine learning and modelling for precision understanding and prediction. J. R. Soc. Interface *15*, 20170736–20170833. https://doi.org/10.1098/rsif.2017.0736.

CellPress
OPEN ACCESS

36. Pattaranit, R., and Van Den Berg, H.A. (2008). Mathematical models of energy homeostasis. J. R. Soc. Interface *5*, 1119–1135. https://doi.org/10.1098/rsif.2008.0216.

37. Dalla Man, C., Caumo, A., and Cobelli, C. (2002). The oral glucose minimal model: Estimation of insulin sensitivity from a meal test. IEEE Trans. Biomed. Eng. *49*, 419–429. https://doi.org/10.1109/10.995680.

38. Dalla Man, C., Rizza, R.A., and Cobelli, C. (2007). Meal simulation model of the glucose-insulin system. IEEE Trans. Biomed. Eng. *54*, 1740–1749. https://doi.org/10.1109/TBME.2007.893506.

39. Deng, Y., Lu, L., Aponte, L., Angelidi, A.M., Novak, V., Karniadakis, G.E., and Mantzoros, C.S. (2021). Deep transfer learning and data augmentation improve glucose levels prediction in type 2 diabetes patients. NPJ Digit. Med. *4*, 109. https://doi.org/10.1038/s41746-021-00480-x.

40. Li, K., Daniels, J., Liu, C., Herrero, P., and Georgiou, P. (2020). Convolutional Recurrent Neural Networks for Glucose Prediction. IEEE J. Biomed. Health Inform. *24*, 603–613. https://doi.org/10.1109/JBHI.2019.2908488.

41. Bent, B., Cho, P.J., Henriquez, M., Wittmann, A., Thacker, C., Feinglos, M., Crowley, M.J., and Dunn, J.P. (2021). Engineering digital biomarkers of interstitial glucose from noninvasive smartwatches. NPJ Digit. Med. *4*, 89–11. https://doi.org/10.1038/s41746-021-00465-w.

42. Rashtian, H., Torbaghan, S.S., Rahili, S., Snyder, M., and Aghaeepour, N. (2021). Heart Rate and CGM Feature Representation Diabetes Detection from Heart Rate: Learning Joint Features of Heart Rate and Continuous Glucose Monitors Yields Better Representations. IEEE Access *9*, 83234–83240. https://doi.org/10.1109/ACCESS.2021.3085544.

43. Sobel, S.I., Chomentowski, P.J., Vyas, N., Andre, D., and Toledo, F.G.S. (2014). Accuracy of a Novel Noninvasive Multisensor Technology to Estimate Glucose in Diabetic Subjects During Dynamic Conditions. J. Diabetes Sci. Technol. *8*, 54–63. https://doi.org/10.1177/1932296813516182.

44. Boughton, C.K., and Hovorka, R. (2021). New closed-loop insulin systems. Diabetologia *64*, 1007–1015. https://doi.org/10.1007/s00125-021-05391-w.

45. Gill, S., and Panda, S. (2015). A Smartphone App Reveals Erratic Diurnal Eating Patterns in Humans that Can Be Modulated for Health Benefits. Cell Metabol. *22*, 789–798. https://doi.org/10.1016/j.cmet.2015.09.005.

46. Chow, L.S., Manoogian, E.N.C., Alvear, A., Fleischer, J.G., Thor, H., Dietsche, K., Wang, Q., Hodges, J.S., Esch, N., Malaeb, S., et al. (2020). Time-Restricted Eating Effects on Body Composition and Metabolic Measures in Humans who are Overweight: A Feasibility Study. Obesity *28*, 860–869. https://doi.org/10.1002/oby.22756.

47. Kruschke, J.K. (2013). Bayesian estimation supersedes the t test. J. Exp. Psychol. Gen. *142*, 573–603. https://doi.org/10.1037/a0029146.

48. Bolie, V.W. (1961). Coefficients of normal blood glucose regulation. J. Appl. Physiol. *16*, 783–788. https://doi.org/10.1152/jappl.1961.16.5.783.

49. Wyatt, P., Berry, S.E., Finlayson, G., O'Driscoll, R., Hadjigeorgiou, G., Drew, D.A., Khatib, H.A., Nguyen, L.H., Linenberg, I., Chan, A.T., et al. (2021). Postprandial glycaemic dips predict appetite and energy intake in healthy individuals. Nat. Metab. *3*, 523–529. https://doi.org/10.1038/s42255-021-00383-x.

50. Bowman, C., Huang, Y., Walch, O.J., Fang, Y., Frank, E., Tyler, J., Mayer, C., Stockbridge, C., Goldstein, C., Sen, S., and Forger, D.B. (2021). A method for characterizing daily physiology from widely used wearables. Cell Rep. Methods *1*, 100058. https://doi.org/10.1016/j.crmeth.2021.100058.

51. Schroeder, E.B., Chambless, L.E., Liao, D., Prineas, R.J., Evans, G.W., Rosamond, W.D., and Heiss, G.; Atherosclerosis Risk in Communities ARIC study (2005). Diabetes, Glucose, Insulin, and Heart Rate Variability. Diabetes Care *28*, 668–674. https://doi.org/10.2337/diacare.28.3.668.

52. Tambascia, M.A., Malerbi, D.A.C., and Eliaschewitz, F.G. (2014). Influence of gastric emptying on the control of postprandial glycemia: physiology and therapeutic implications. Einstein (São Paulo, Brazil) *12*, 251–253. https://doi.org/10.1590/S1679-45082014RB2862.

53. Moore, M.C., Coate, K.C., Winnick, J.J., An, Z., and Cherrington, A.D. (2012). Regulation of hepatic glucose uptake and storage in vivo. Adv. Nutr. *3*, 286–294. https://doi.org/10.3945/an.112.002089.

54. Bansal, P., and Wang, Q. (2008). Insulin as a physiological modulator of glucagon secretion. Am. J. Physiol. Endocrinol. Metab. *295*, E751–E761. https://doi.org/10.1152/ajpendo.90295.2008.

55. Petrenko, V., Gandasi, N.R., Sage, D., Tengholm, A., Barg, S., and Dibner, C. (2020). In pancreatic islets from type 2 diabetes patients, the dampened circadian oscillators lead to reduced insulin and glucagon exocytosis. Proc. Natl. Acad. Sci. USA *117*, 2484–2495. https://doi.org/10.1073/pnas.1916539117.

56. Saad, A., Dalla Man, C., Nandy, D.K., Levine, J.A., Bharucha, A.E., Rizza, R.A., Basu, R., Carter, R.E., Cobelli, C., Kudva, Y.C., and Basu, A. (2012). Diurnal pattern to insulin secretion and insulin action in healthy individuals. Diabetes *61*, 2691–2700. https://doi.org/10.2337/db11-1478.

57. Sinturel, F., Makhlouf, A.M., Meyer, P., Tran, C., Pataky, Z., Golay, A., Rey, G., Howald, C., Dermitzakis, E.T., Pichard, C., et al. (2019). Cellular circadian period length inversely correlates with HbA1c levels in individuals with type 2 diabetes. Diabetologia *62*, 1453–1462. https://doi.org/10.1007/s00125-019-4907-0.

58. Isherwood, C.M., van der Veen, D.R., Hassanin, H., Skene, D.J., and Johnston, J.D. (2023). Human glucose rhythms and subjective hunger anticipate meal timing. Curr. Biol. *33*, 1321–1326.e3. https://doi.org/10.1016/j.cub.2023.02.005.

59. Chaix, A., Manoogian, E.N.C., Melkani, G.C., and Panda, S. (2019). Time-Restricted Eating to Prevent and Manage Chronic Metabolic Diseases. Annu. Rev. Nutr. *39*, 291–315. https://doi.org/10.1146/annurev-nutr-082018-124320.

60. Grant, A.D., Upton, T.J., Terry, J.R., Smarr, B.L., and Zavala, E. (2022). Analysis of wearable time series data in endocrine and metabolic research. Curr. Opin. Endocr. Metab. Res. *25*, 100380. https://doi.org/10.1016/j.coemr.2022.100380.

61. Leise, T.L. (2013). Wavelet analysis of circadian and ultradian behavioral rhythms. J. Circadian Rhythms *11*, 5. https://doi.org/10.1186/1740-3391-11-5.

62. Farabi, S.S., Carley, D.W., and Quinn, L. (2017). Glucose Variations and Activity Are Strongly Coupled in Sleep and Wake in Young Adults With Type 1 Diabetes. Biol. Res. Nurs. *19*, 249–257. https://doi.org/10.1177/1099800416685177.

63. Soru, P., De Nicolao, G., Toffanin, C., Dalla Man, C., Cobelli, C., and Magni, L. (2012). MPC based Artificial Pancreas: Strategies for individualization and meal compensation. Annu. Rev. Control *36*, 118–128. https://doi.org/10.1016/j.arcontrol.2012.03.009.

64. Toffanin, C., Messori, M., Di Palma, F., De Nicolao, G., Cobelli, C., and Magni, L. (2013). Artificial pancreas: Model predictive control design from clinical experience. J. Diabetes Sci. Technol. *7*, 1470–1483. https://doi.org/10.1177/193229681300700607.

65. Coorey, G., Figtree, G.A., Fletcher, D.F., and Redfern, J. (2021). The health digital twin: advancing precision cardiovascular medicine. Nat. Rev. Cardiol. *18*, 803–804. https://doi.org/10.1038/s41569-021-00630-4.

66. Kamel Boulos, M.N., and Zhang, P. (2021). Digital twins: From personalised medicine to precision public health. J. Personalized Med. *11*, 745. https://doi.org/10.3390/jpm11080745.

67. Roy, A., and Parker, R.S. (2007). Dynamic modeling of exercise effects on plasma glucose and insulin levels. J. Diabetes Sci. Technol. *1*, 338–347. https://doi.org/10.1177/193229680700100305.

68. Zhu, T., Uduku, C., Li, K., Herrero, P., Oliver, N., and Georgiou, P. (2022). Enhancing self-management in type 1 diabetes with wearables and deep learning. NPJ Digit. Med. *5*, 78–11. https://doi.org/10.1038/s41746-022-00626-5.

69. Craig, C.L., Marshall, A.L., Sjöström, M., Bauman, A.E., Booth, M.L., Ainsworth, B.E., Pratt, M., Ekelund, U., Yngve, A., Sallis, J.F., and Oja, P. (2003). International physical activity questionnaire: 12-Country reliability

and validity. Med. Sci. Sports Exerc. *35*, 1381–1395. https://doi.org/10.1249/01.MSS.0000078924.61453.FB.

70. Roenneberg, T., Wirz-Justice, A., and Merrow, M. (2003). Life between clocks: Daily temporal patterns of human chronotypes. J. Biol. Rhythm. *18*, 80–90. https://doi.org/10.1177/0748730402239679.

71. Buysse, D.J., Reynolds, C.F., Monk, T.H., Berman, S.R., and Kupfer, D.J. (1989). The Pittsburgh sleep quality index: A new instrument for psychiatric practice and research. Psychiatr. Res. *28*, 193–213. https://doi.org/10.1016/0165-1781(89)90047-4.

72. De, A.G., Matthews, G., Nickson, T., Fujii, K., Boukouvalas, A., León-Villagrá, P., Ghahramani, Z., and Hensman, J. (2017). GPflow: A Gaussian Process Library using TensorFlow Mark van der Wilk. J. Mach. Learn. Res. *18*, 1–6. https://doi.org/10.48550/arXiv.1610.08733.

73. Särkkä, S., and Solin, A. (2019). Applied Stochastic Differential Equations (Cambridge University Press). https://doi.org/10.1007/0-8176-4428-8_4.

74. Abadi, M., Agarwal, A., Paul Barham, E.B., Chen, Z., Craig, C., Greg, S., Corrado, A.D., Dean, J., and Matthieu Devin, S.G. (2015). TensorFlow: Large-Scale Machine Learning on Heterogeneous Distributed Systems. https://doi.org/10.1080/09548980500300507.

75. Kass, R.E., and Raftery, A.E. (1995). Bayes Factors. J. Am. Stat. Assoc. *90*, 773–795. https://doi.org/10.1080/01621459.1995.10476572.

76. Andrieu, C., and Thoms, J. (2008). A tutorial on adaptive MCMC. Stat. Comput. *18*, 343–373. https://doi.org/10.1007/s11222-008-9110-y.

STAR★METHODS

KEY RESOURCES TABLE

REAGENT or RESOURCE	SOURCE	IDENTIFIER
Deposited data		
Food app, CGM and ActiHeart data	This paper	https://github.com/naef-lab/MultiSensor https://doi.org/10.5281/zenodo.8028677
Software and algorithms		
Python version 3.7.4	Python Software Foundation	https://www.python.org
The MultiSensor Study code	This paper	https://github.com/naef-lab/MultiSensor https://doi.org/10.5281/zenodo.8028677

RESOURCE AVAILABILITY

Lead contact

Further information and requests for resources should be directed to and will be fulfilled when possible by the lead contact, Felix Naef (felix.naef@epfl.ch).

Materials availability

This study did not generate new physical materials.

Data and code availability

- De-identified participant data has been deposited at https://github.com/naef-lab/MultiSensor and at https://doi.org/10.5281/zenodo.8028677.
- All original code has been deposited at https://github.com/naef-lab/MultiSensor and at https://doi.org/10.5281/zenodo.8028677.
- Any additional information required to implement the method of this study is available from the lead contact upon request.

EXPERIMENTAL MODEL AND STUDY PARTICIPANT DETAILS

The Multi-Sensor Study (MSS) was approved by the local ethics committee (CER-VD, BASEC no. 2019-02245) and each participant signed a written informed consent. Recruitment was performed via posters at the École Polytechnique Fédérale de Lausanne (EPFL), Lausanne University Hospital (CHUV) and the University of Lausanne (UNIL) and via presentations given in the EPFL School of Life Sciences.

We included adults aged $\geq$ 18 years, with a smartphone compatible with the myCircadianClock app (iOS or Android systems[45]) and able to take pictures of food/drinks, and who self-identified as disciplined enough and motivated to record all data for two weeks. The exclusion criteria were major illness/fever, surgery over the previous month, eating disorder, major mental illness, unable to give informed consent, taking medicines including paracetamol, aspirin or vitamin C supplements, enrolled in another interventional clinical trial (medication, medical device), shift work or travel to a different time zone before and during the study. 25 participants (16 males, 9 females) were recruited, and participant characteristics are shown in Figure S1.

METHOD DETAILS

Devices and experimental design

At baseline, we collected data on demographics, medical history, physical activity (short form of International Physical Activity Questionnaire, IPAQ-SF),[69] chronotype (The Munich ChronoType Questionnaire),[70] sleeping habits (Pittsburgh Sleep Quality Index)[71] and eating timing (with a custom questionnaire on eating habits during work and free days).

For each participant, we collected data for two weeks using the following devices: 1) Timestamps of food/drinks and text annotations collected with the smartphone application (app) myCircadianClock[45]; 2) Continuous glucose monitoring (CGM) using the Abbott FreeStyle Libre Pro device; 3) Physical activity, heart rate (HR) and heart rate variability (HRV using RMSSD^{-1}) using the CamNtech ActiHeart device version 5. Participants were instructed to take pictures of all consumed food and drink with the research-dedicated myCircadianClock smartphone. Recorded entries included a timestamped picture and a free-text annotation,

and entries with the same annotation were considered as the same meal type. Participants could annotate photographs either immediately or in the following hours. Optionally, participants could type text-only entries without any picture, e.g., if the smartphone ran out of battery, or if it was not socially acceptable to take pictures in the current context. Participants were also asked to optionally log physical exercise using the app. While the CamNtech Actiheart device is waterproof, participants were permitted to briefly remove the device during showers and baths. Specific information on individual device technical failure, handling of missing data and data quality is included in the Supplementary Information (Methods S1).

Pre-processing CGM data

We used nonparametric regression with Gaussian processes (GPs) to remove the long-term trends observed in the data. After mean-centring the data, we fitted a GP with a squared exponential kernel $K_{SE}(t,t') = (-|t - t'|^2/2l^2)$ and a length scale l=48 hours using GPflow.[72]

Data analysis

See the Supplementary Information (Methods S1) for detailed computational methods which are summarised here. We use a linear Gaussian state space model (otherwise known as a Kalman filter[73]) to analyse the time series generated by the wearable devices, which was implemented using the 'LinearGaussianStateSpaceModel' distribution within TensorFlow Probability.[74] We will first describe the general data analysis framework before providing details on each of the three models used (Models 1-3). For each model we define a dynamic model that describes the time evolution of the underlying physiological variables and a measurement model that incorporates measurement noise. For the dynamic model, we use a system of stochastic differential equations (SDEs).

$$\mathrm{d}\boldsymbol{x}(t) = W\boldsymbol{x}(t)\mathrm{d}t + \mathrm{d}\boldsymbol{\beta}, \qquad \text{(Equation 1)}$$

where W is a matrix describing the interactions between the variables $\boldsymbol{x}(t)$, and $\boldsymbol{\beta}$ is a brownian noise term with covariance matrix Q. The specific forms of W and Q are unique for each model and will be described below. To keep the model exact while benefiting from the generic framework of Gaussian state space models (a.k.a. as Kalman filters), we then convert this system of continuous-time SDEs into a model where time is discrete (see Methods S1 for details).

$$\boldsymbol{x}(t_k) = F_{k-1}\boldsymbol{x}(t_{k-1}) + N(0, \Sigma_{k-1}), \qquad \text{(Equation 2)}$$

where F_k is the state-transition model and Σ_k is the covariance of the process noise. The measurement model describes the observation process and assumes that variables are observed with normally distributed measurement noise

$$\boldsymbol{y}(t_k) = H_k\boldsymbol{x}(t_k) + N(\boldsymbol{m}_k, R_k), \qquad \text{(Equation 3)}$$

where H_k is the observation matrix and $\boldsymbol{m_k}$ and R_k represents the mean and covariance of the observation noise, respectively. The goal is to use the wearable time series data $\boldsymbol{y}_{1:T}$ to estimate parameters (denoted by $\boldsymbol{\theta}$) for each participant. Within a Bayesian inference framework, the parameters of the model can be estimated from the data as follows

$$p(\boldsymbol{\theta}|\boldsymbol{y}_{1:T}) \propto p(\boldsymbol{\theta})p(\boldsymbol{y}_{1:T}|\boldsymbol{\theta}), \qquad \text{(Equation 4)}$$

where $p(\boldsymbol{\theta})$ is the prior distribution of parameters and $p(\boldsymbol{y}_{1:T}|\boldsymbol{\theta})$ is the likelihood of observing the temporal data $\boldsymbol{y}_{1:T}$ given the set of parameters $\boldsymbol{\theta}$. Considering the time series sequence of data, the likelihood term for a given set of parameters $\boldsymbol{\theta}$ can be expressed as

$$p(\boldsymbol{y}_{1:T}|\boldsymbol{\theta}) = p(\boldsymbol{y}_1|\boldsymbol{\theta}) \prod_{k=2}^{T} p(\boldsymbol{y}_k|\boldsymbol{y}_{1:k-1}, \boldsymbol{\theta}), \qquad \text{(Equation 5)}$$

and the sequence of distributions $p(\boldsymbol{y}_k|\boldsymbol{y}_{1:k-1}, \boldsymbol{\theta})$ are calculated within a Kalman filtering framework. Once the likelihood and priors are specified for each model, we used the Hamiltonian Monte Carlo sampler provided within TensorFlow Probability to sample model parameters from the posterior distribution (described below in quantification and statistical analysis). The priors for all models are specified in Methods S1.

Model 1: Glucose model

We model glucose dynamics (Figure 2A) with a two-dimensional system of SDEs, where the second variable x_{GLUC2} represents the glucose levels and the first variable x_{GLUC1} represents an unobserved latent variable that allows negative feedback within the system. In matrix form, the model is expressed as follows

$$\mathrm{d}\boldsymbol{x}(t) = W\boldsymbol{x}(t)\mathrm{d}t + \mathrm{d}\boldsymbol{\beta},$$
$$\boldsymbol{x}(t) = \begin{bmatrix} x_{\mathrm{GLUC1}} \\ x_{\mathrm{GLUC2}} \end{bmatrix},$$
$$W = \begin{bmatrix} -A_{11} & -A_{12} \\ A_{21} & -A_{22} \end{bmatrix}, \qquad \text{(Equation 6)}$$
$$Q = \begin{bmatrix} 0 & 0 \\ 0 & B_{22} \end{bmatrix},$$

and where the coefficients A_{ij} are constrained to be positive. The covariance of the brownian noise term β is given by Q. The 'damping coefficient' is determined by whether the eigenvalues of the matrix W are real or complex. For the 2x2 matrix W, this damping coefficient can be determined by $-\det(W - I\,\mathrm{tr}(W)/2)/(\mathrm{tr}(W)/2)^2$.

The smartphone application provides a list of the recorded ingestion event times $\{t_m\}_{m=1}^{M}$ for a total of M meals. We incorporate meal events (recorded at time t_m) as producing a response function $r_m(t, t_m, \boldsymbol{\theta})$ by perturbing the first variable x_{GLUC1} to higher values (see Methods S1 for precise functional form), and then the total meal function is the sum over all M individual meal responses

$$r(t) = \sum_{m=1}^{M} r_m(t, t_m, \boldsymbol{\theta}). \qquad \text{(Equation 7)}$$

We define the glucose half-life parameter as the model-predicted time to return to 0.5 mol/L after a standardised increase of 1mmol/L. We also add an underlying circadian trend in glucose levels using a sinusoidal function

$$g_{\mathrm{GLUC}}(t) = A_{0,\mathrm{GLUC}} + A_{1,\mathrm{GLUC}}(1 + \cos(\omega t - \varphi_{\mathrm{GLUC}}))/2 \qquad \text{(Equation 8)}$$

where $A_{0,\mathrm{GLUC}}$ is the baseline level, $A_{1,\mathrm{GLUC}}$ is the amplitude, ω is the frequency (fixed at $2\pi/24$), and φ_{GLUC} is the peak time of the maximum. The observation model for the glucose model is then as follows

$$\boldsymbol{y}(t_k) = H_k\boldsymbol{x}(t_k) + N(\boldsymbol{m}_k, R_k),$$
$$H_k = [0\ 1]$$
$$\boldsymbol{m}_k = r(t) + g_{\mathrm{GLUC}}(t), \qquad \text{(Equation 9)}$$
$$R_k = \sigma^2_{\mathrm{GLUC}},$$

We compared Model 1 with an alternative version without circadian oscillations using the Bayesian Information Criterion (BIC) $\mathrm{BIC} = k\ln(n) - 2\ln(p(\boldsymbol{y}_{1:T}|\boldsymbol{\theta}))$, where k is the number of parameters and n is the number of data points. We calculated the difference in BIC score using Model 1 both with and without a circadian components and used a cut-off of $2\ln(10)$ to indicate that the strength of evidence favoured a particular model.[75]

Model 2: Physical and heart activity model

We model physical and heart activity dynamics (Figure 2B) with a three-dimensional system of SDEs, where the first variable x_{ACT} represents physical activity, the second variable x_{HR} represents heart rate and the third variable x_{HRV} represents heart rate variability, where we use the inverse of the root mean square of successive differences between normal heartbeats (RMSSD^{-1}). We normalise all three variables by their respective standard deviations before inferring parameters. In matrix form, the model is expressed as follows

$$\mathrm{d}\boldsymbol{x}(t) = W\boldsymbol{x}(t)\mathrm{d}t + \mathrm{d}\boldsymbol{\beta},$$
$$\boldsymbol{x}(t) = \begin{bmatrix} x_{\mathrm{ACT}} \\ x_{\mathrm{HR}} \\ x_{\mathrm{HRV}} \end{bmatrix},$$
$$W = \begin{bmatrix} -C_{11} & 0 & 0 \\ C_{21} & -C_{22} & 0 \\ C_{31} & 0 & -C_{33} \end{bmatrix}, \qquad \text{(Equation 10)}$$
$$Q = \begin{bmatrix} D_{11} & 0 & 0 \\ 0 & D_{22} & \rho\sqrt{D_{22}D_{33}} \\ 0 & \rho\sqrt{D_{22}D_{33}} & D_{33} \end{bmatrix},$$

and where the coefficients C_{ij} are constrained to be positive and the covariance of the brownian noise term β is given by Q. In the model, the correlation in the fluctuations between HR and HRV is quantified with the correlation parameter ρ. The observation model is then given by

$$\begin{aligned} \boldsymbol{y}(t_k) &= H_k \boldsymbol{x}(t_k) + N(\boldsymbol{m}_k, R_k), \\ H_k &= \begin{bmatrix} 1 & 0 & 0 \\ 0 & 1 & 0 \\ 0 & 0 & 1 \end{bmatrix}, \\ \boldsymbol{m}_k &= \begin{bmatrix} g_{\mathrm{ACT}}(t) \\ g_{\mathrm{HR}}(t) \\ g_{\mathrm{HRV}}(t) \end{bmatrix}, \\ R_k &= \begin{bmatrix} \sigma^2_{\mathrm{ACT}} & 0 & 0 \\ 0 & \sigma^2_{\mathrm{HR}} & 0 \\ 0 & 0 & \sigma^2_{\mathrm{HRV}} \end{bmatrix}, \end{aligned} \quad \text{(Equation 11)}$$

where $g_{\mathrm{ACT}}(t)$, $g_{\mathrm{HR}}(t)$ and $g_{\mathrm{HRV}}(t)$ are circadian oscillatory functions (Methods S1).

Model 3: Combined model

The final model (Figure 2C) connects the physical and heart activity signals with CGM dynamics by stitching the previous glucose (Model 1) and physical and heart activity models (Model 2) together. Both models are otherwise left unchanged, but there is an introduction of three new parameters C_{51}, C_{52} and C_{53} that describe the effect of physical activity, HR and HRV on glucose levels, respectively. These three parameters are left unconstrained and can take either positive or negative values. To simplify the model inference problem, the parameters from Model 2 describing the physical and heart activity model in isolation were locked to their posterior mean values.

$$\begin{aligned} \mathrm{d}\boldsymbol{x}(t) &= W\boldsymbol{x}(t)\mathrm{d}t + \mathrm{d}\boldsymbol{\beta}, \\ \boldsymbol{x}(t) &= \begin{bmatrix} x_{\mathrm{ACT}} \\ x_{\mathrm{HR}} \\ x_{\mathrm{HRV}} \\ x_{\mathrm{GLUC1}} \\ x_{\mathrm{GLUC2}} \end{bmatrix}, \\ W &= \begin{bmatrix} -C_{11} & 0 & 0 & 0 & 0 \\ C_{21} & -C_{22} & 0 & 0 & 0 \\ C_{31} & 0 & -C_{33} & 0 & 0 \\ 0 & 0 & 0 & -A_{11} & -A_{12} \\ 0 & 0 & 0 & A_{21} & -A_{22} \end{bmatrix}, \\ Q &= \begin{bmatrix} D_{11} & 0 & 0 & 0 & 0 \\ 0 & D_{22} & \rho\sqrt{D_{22}D_{33}} & 0 & 0 \\ 0 & \rho\sqrt{D_{22}D_{33}} & D_{33} & 0 & 0 \\ 0 & 0 & 0 & 0 & 0 \\ 0 & 0 & 0 & 0 & B_{22} \end{bmatrix}, \end{aligned} \quad \text{(Equation 12)}$$

The observation model is then given by

$$\boldsymbol{y}(t_k) = H_k \boldsymbol{x}(t_k) + N(\boldsymbol{m}_k, R_k),$$
$$H_k = \begin{bmatrix} 1 & 0 & 0 & 0 & 0 \\ 0 & 1 & 0 & 0 & 0 \\ 0 & 0 & 1 & 0 & 0 \\ 0 & 0 & 0 & 0 & 1 \end{bmatrix},$$
$$\boldsymbol{m}_k = \begin{bmatrix} g_{\mathrm{ACT}}(t) \\ g_{\mathrm{HR}}(t) \\ g_{\mathrm{HRV}}(t) \\ r(t)+g_{\mathrm{GLUC}}(t) \end{bmatrix}, \quad \text{(Equation 13)}$$
$$R_k = \begin{bmatrix} \sigma^2_{\mathrm{ACT}} & 0 & 0 & 0 \\ 0 & \sigma^2_{\mathrm{HR}} & 0 & 0 \\ 0 & 0 & \sigma^2_{\mathrm{HRV}} & 0 \\ 0 & 0 & 0 & \sigma^2_{\mathrm{GLUC}} \end{bmatrix},$$

QUANTIFICATION AND STATISTICAL ANALYSIS

The wearable data for each participant is analysed separately and the inferred parameters are presented along with the uncertainty for each individual as obtained through Markov Chain Monte Carlo (MCMC) sampling. The parameter posterior distribution was sampled using Hamiltonian Markov Chain Monte Carlo (HMC), which uses the gradients of the posterior to improve the efficiency of the sampling. To initialise the sampler, we found the maximum a posteriori probability (MAP) parameter estimate using the BFGS optimiser 'bfgs_minimize' within TensorFlow Probability. We then used the 'HamiltonianMonteCarlo' function with TensorFlow Probability with 5 leapfrog steps, and we scaled the step size of each variable to approximately match the standard deviation of the posterior distribution. To achieve this, we sampled posterior parameters using two steps. Firstly, we sampled 10,000 parameters (with a burn-in of 10,000 samples) using the 'SimpleStepSizeAdaptation' kernel to select the global step size, which adapts the global step size to achieve a target acceptance probability of 0.75.[76] We then scaled the step size of each variable according to the standard deviation of this initial posterior distribution. Next, we resampled model parameters from the posterior distribution using 4 different chains with 10,000 samples each (with a burn-in of 10,000 samples), again using 'SimpleStepSizeAdaptation' kernel to globally rescale the step size. The 'SimpleStepSizeAdaptation' kernel was only applied to first 80% of the burn-in samples. From the MCMC samples, the percentiles of the posterior parameter distributions are shown graphically for each participant with boxplots. We then estimate the explained variance using $1 - \mathrm{Var}(\boldsymbol{y} - \tilde{\boldsymbol{y}})/\mathrm{Var}(\boldsymbol{y})$, using the model predictions $\tilde{\boldsymbol{y}}$ from the MCMC parameter samples.

Cell Reports Methods, Volume *3*

Supplemental information

Uncovering personalized glucose responses and circadian rhythms from multiple wearable biosensors with Bayesian dynamical modeling

Nicholas E. Phillips, Tinh-Hai Collet, and Felix Naef

1 Supplementary Tables

Study ID	MSF_{SC} (hrs)	IPAQ (METs-hrs/wk)	PSQI	App adherence (%)	Valid Actiheart (%)
01	4.8	13.65	7	100.0	72.5
02	5.5	9.65	2	100.0	58.6
03	3.3	5.30	1	100.0	59.7
04	4.9	15.30	7	100.0	80.5
05	5.0	37.65	3	100.0	85.5
06	5.3	9.49	4	100.0	73.3
07	3.3	35.30	4	100.0	Not used
08	4.2	24.95	5	100.0	70.3
09	3.7	2.47	7	100.0	93.7
10	4.6	53.10	7	100.0	90.8
11	3.6	3.10	3	100.0	72.4
12	5.6	28.95	2	100.0	94.4
13	4.5	14.47	9	100.0	83.4
14	2.3	10.95	2	100.0	74.3
15	4.7	23.30	5	100.0	41.4
16	3.6	39.30	5	100.0	89.6
17	4.5	5.00	5	83.3	82.6
18	5.0	25.10	4	100.0	85.6
19	3.8	18.60	4	100.0	91.3
20	4.2	9.60	8	100.0	89.2
21	4.5	14.16	5	100.0	60.9
22	5.8	3.22	6	Not used	81.3
23	4.3	24.95	7	100.0	86.3
24	2.5	28.82	5	100.0	74.6
25	4.2	9.65	5	100.0	91.8

Table S1: Chronotype and data quality metrics for study participants. Related to STAR Methods. MSF_{SC} (hours): chronotype variable calculated from the Munich Chronotype Questionnaire (MCTQ); IPAQ: physical activity as measured with the International Physical Activity Questionnaire (IPAQ); PSQI: sleep quality as assessed with the Pittsburgh Sleep Quality Index. Range 0-27, where higher scores reflect lower quality sleep.; App adherence (%): the percentage of days with two recorded meals separated by at least 5 hours, calculated using all valid study days and excluding the first and last study days; Valid Actiheart (%): the percentage of Actiheart data that passes quality filtering for each participant.

2 Supplementary Figures

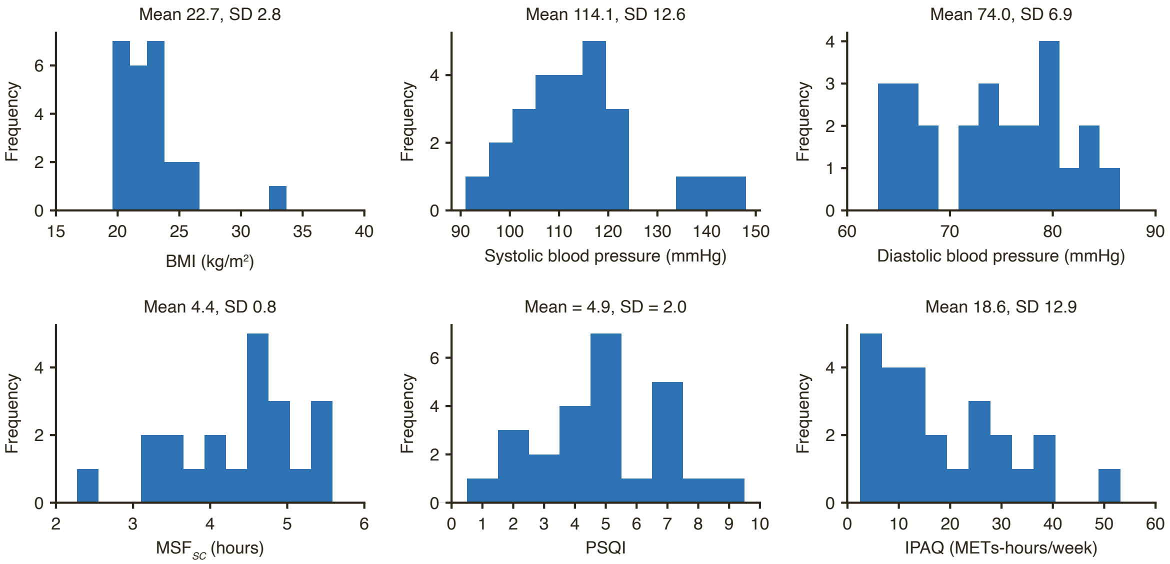

Figure S1: The distribution of participant characteristics in the study population, displayed as histograms. Related to STAR Methods. BMI: body mass index. MSF_{SC} (hours): chronotype variable calculated from the Munich Chronotype Questionnaire (MCTQ); PSQI: sleep quality as assessed with the Pittsburgh Sleep Quality Index. Range 0-27, where higher scores reflect lower quality sleep; IPAQ: physical activity as measured with the International Physical Activity Questionnaire (IPAQ).

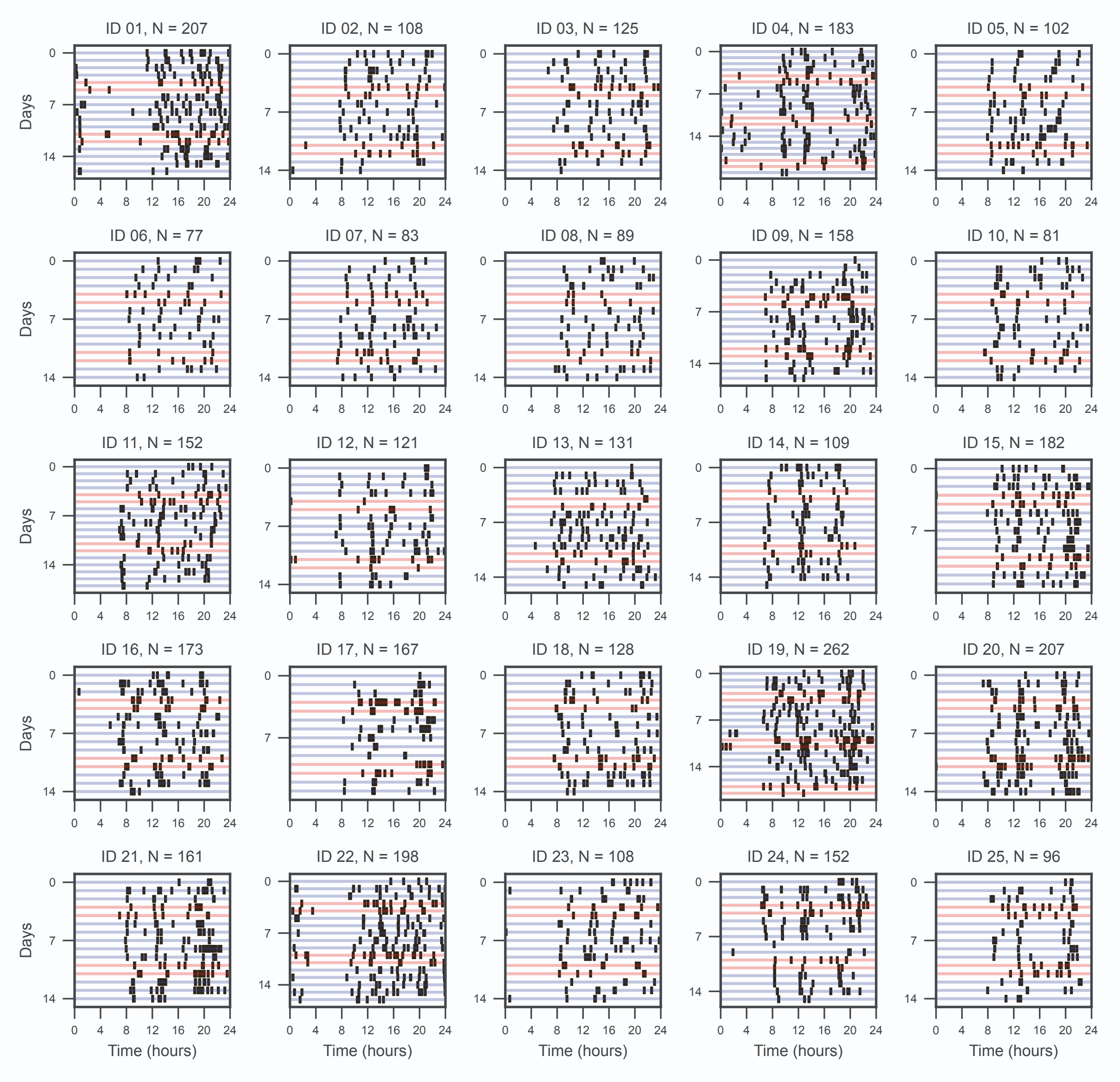

Figure S2: All recorded food and drink events for all study participants. Related to Figure 1. Each horizontal line represents a different study day, and food and drink events are marked as vertical lines. Day 0 represents the first study day. Blue horizontal lines denote weekdays while red horizontal lines represent the weekend. The total number of recorded ingestion events is indicated with N.

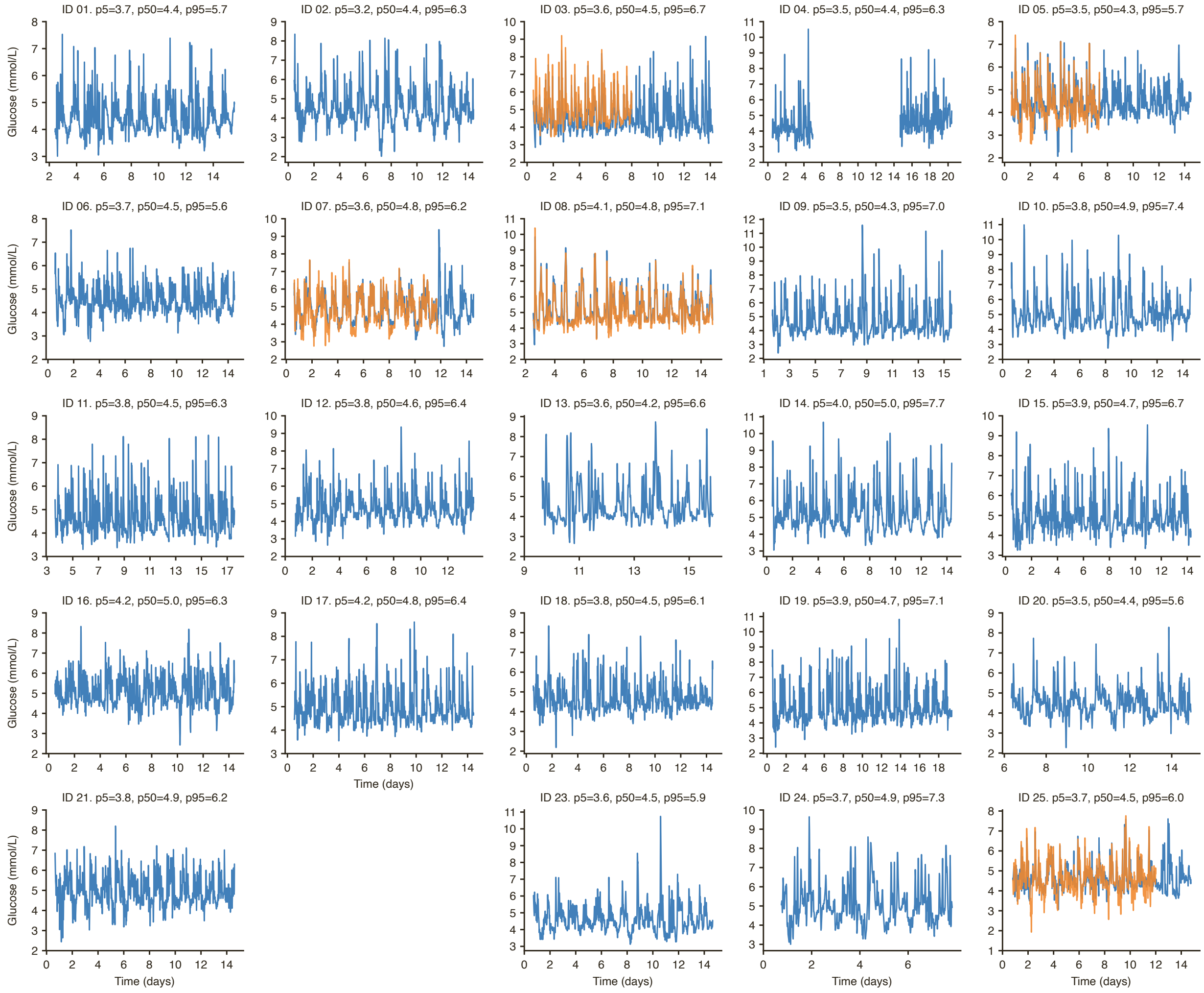

Figure S3: CGM data for all study participants. Related to Figure 1. For each study participant, the 5th, 50th and 95th percentiles are indicated along with the study ID. For five participants there is a second sensor (orange line). Device failure for ID 22 meant that the data is missing for this participant, and an empty space is left.

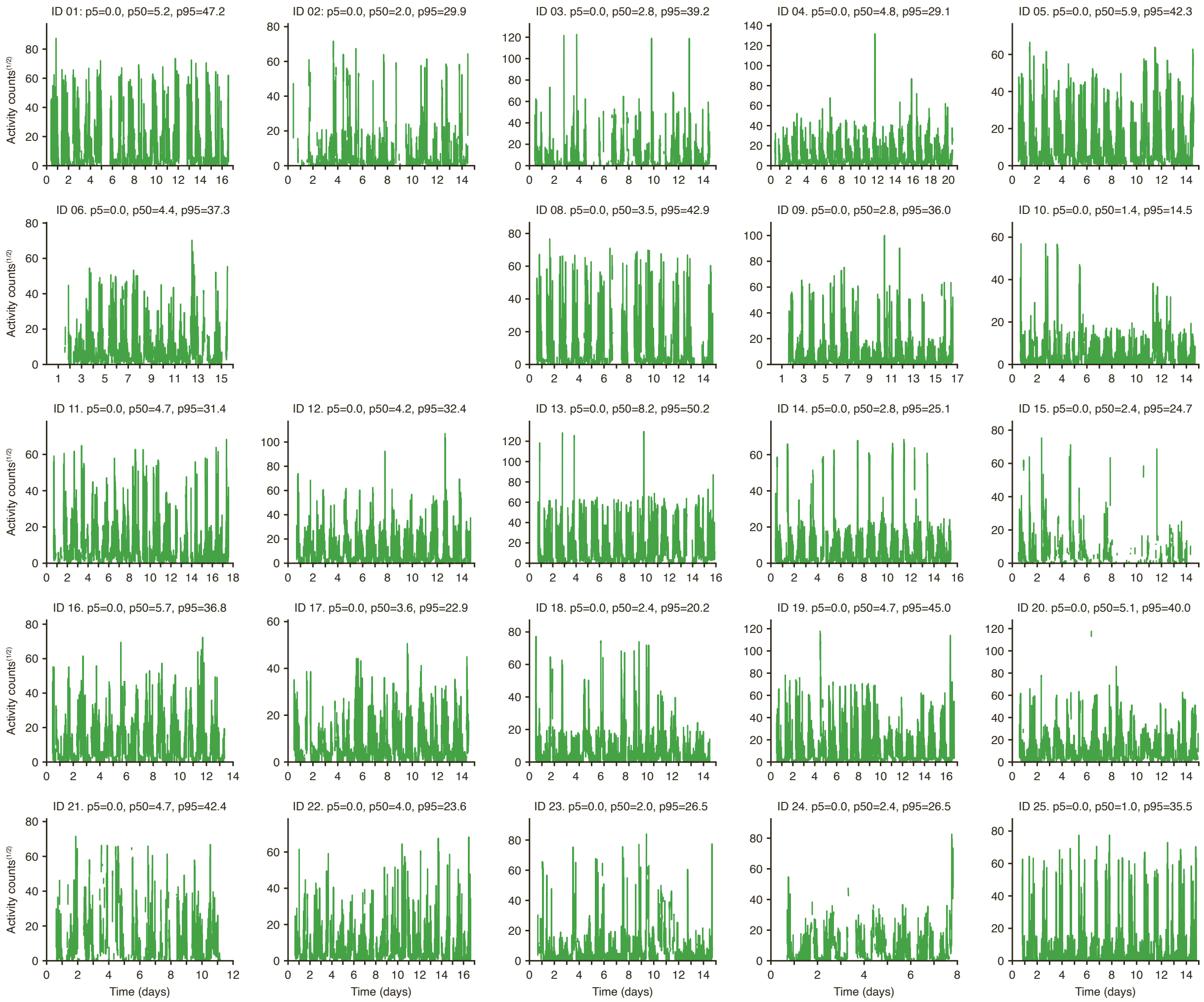

Figure S4: Activity data for all study participants, as measured with the Actiheart device. Related to Figure 1. For each study participant, the 5th, 50th and 95th percentiles are indicated along with the study ID. Device failure for ID 07 meant that the data is missing for this participant, and an empty space is left.

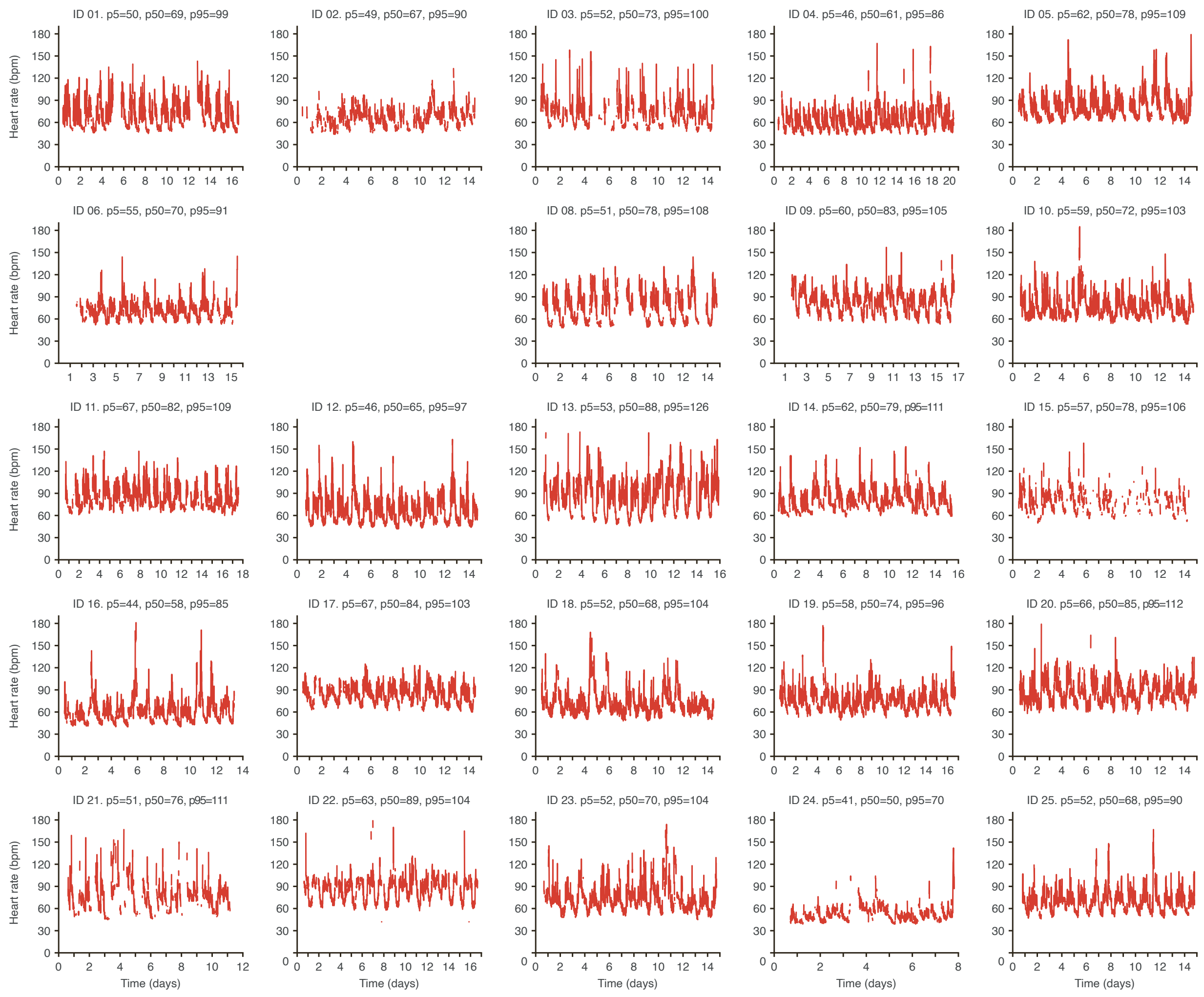

Figure S5: Heart rate data for all study participants (bpm: beats per minute), as measured with the Actiheart device. Related to Figure 1. For each study participant, the 5th, 50th and 95th percentiles are indicated along with the study ID. Device failure for ID 07 meant that the data is missing for this participant, and an empty space is left.

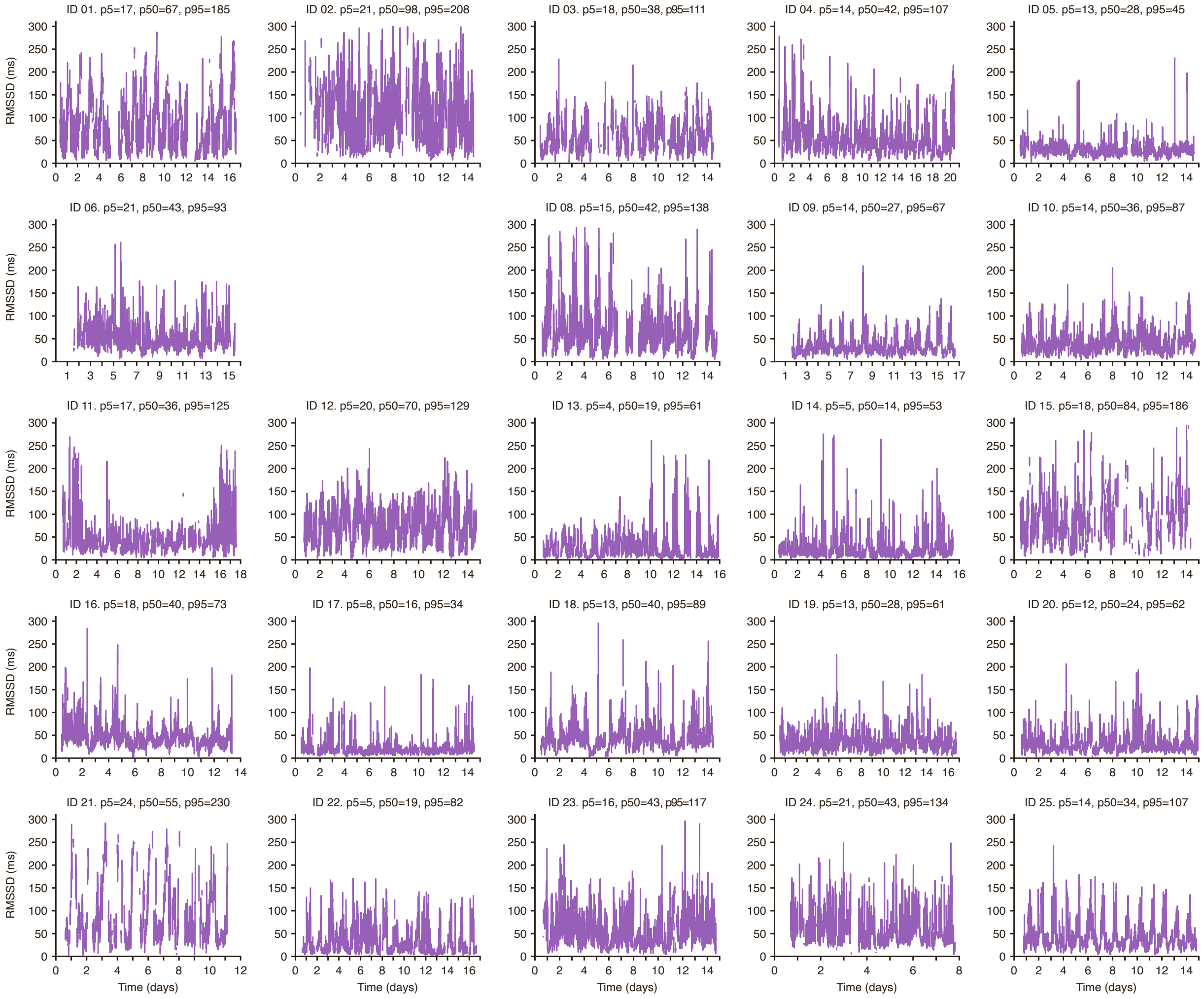

Figure S6: Heart rate variability data for all study participants (RMSSD: root mean square of successive differences between heartbeats), as measured with the Actiheart device. Related to Figure 1. For each study participant, the 5, 50 and 95% percentiles are indicated along with the study ID. Device failure for ID 07 meant that the data is missing for this participant, and an empty space is left.

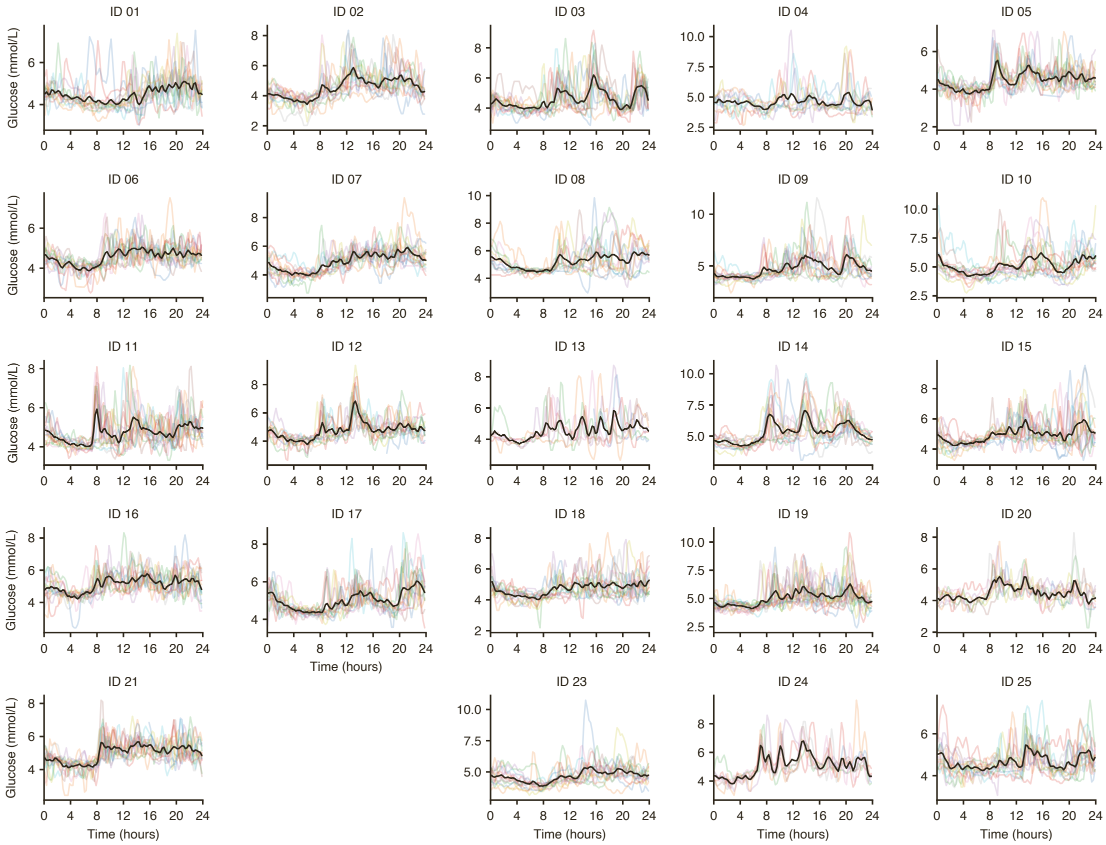

Figure S7: Glucose time-series data over a two-week period for all participants. Related to Figure 1. Individual days are represented by different coloured lines and the mean glucose level over all measured days in black.

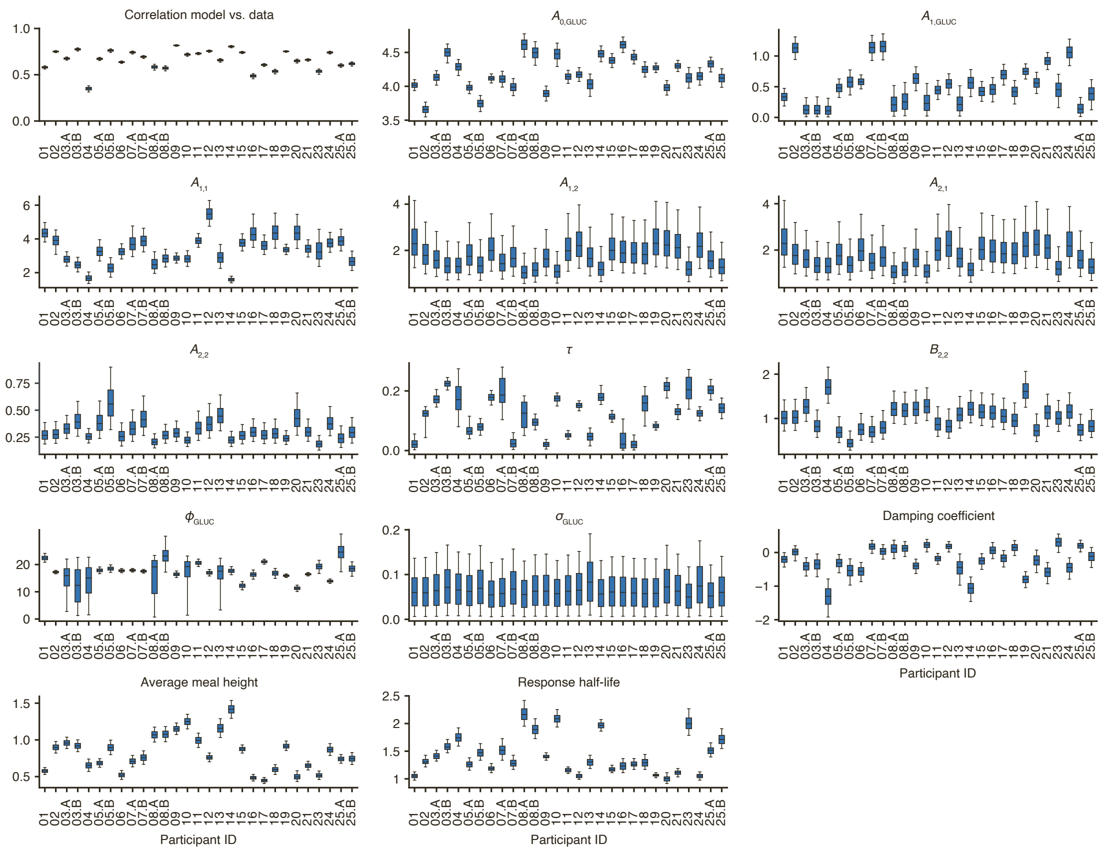

Figure S8: Posterior distributions (shown as boxplots) for all glucose model parameters (Model 1) across all participants. Related to Figure 3. The first panel shows the correlation between the inferred model (including meals and circadian time) and the glucose CGM data for each participant. The boxplots represent the 25th, median (50th) and 75th percentiles of the posterior distribution and the whiskers represent the 5th and 95th percentiles.

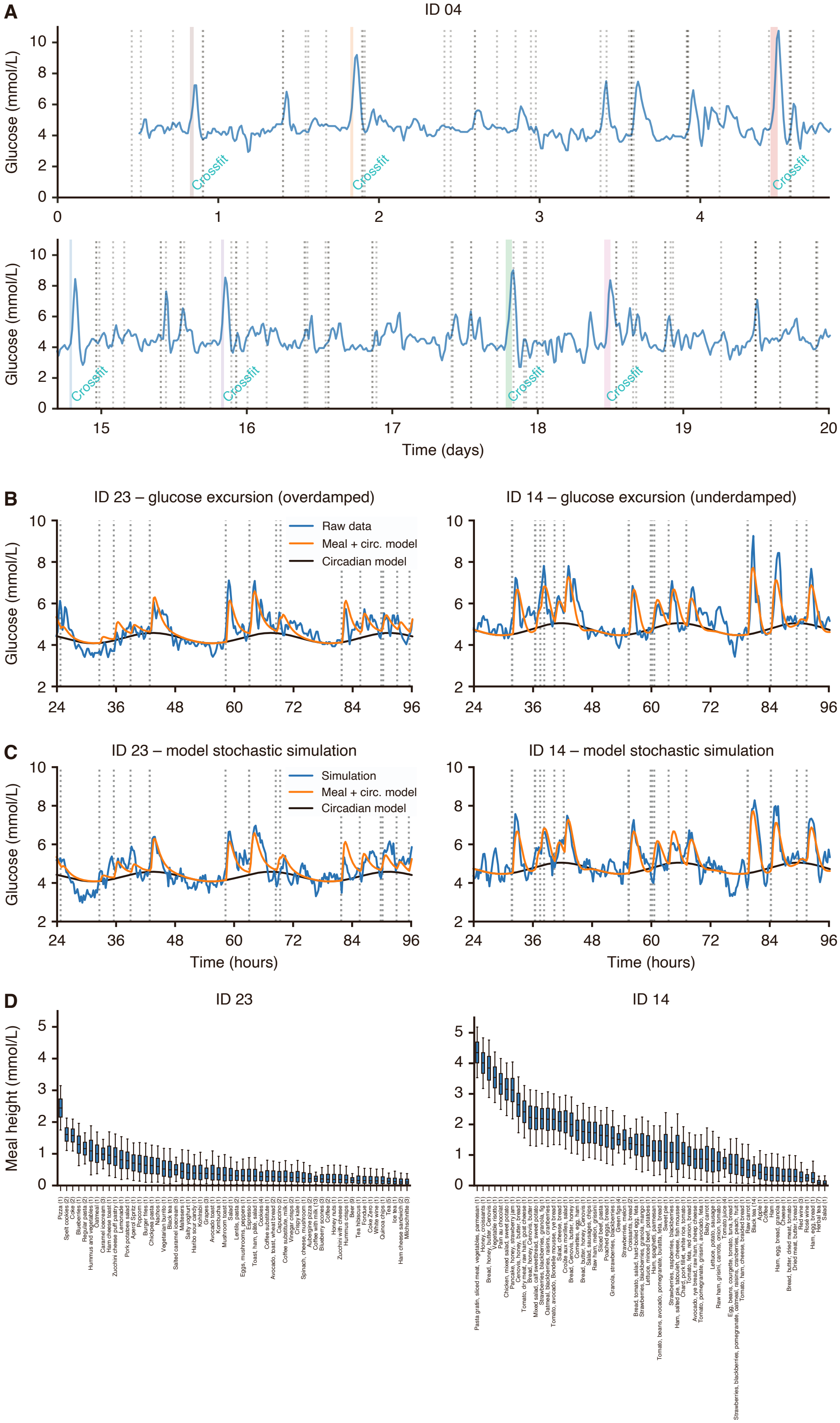
A
ID 04
Glucose (mmol/L)
Crossfit
Time (days)
B
ID 23 – glucose excursion (overdamped)
ID 14 – glucose excursion (underdamped)
Raw data
Meal + circ. model
Circadian model
Glucose (mmol/L)
C
ID 23 – model stochastic simulation
ID 14 – model stochastic simulation
Simulation
Meal + circ. model
Circadian model
Glucose (mmol/L)
Time (hours)
D
ID 23
ID 14
Meal height (mmol/L)

Figure S9: Accessory figures related to Figure 3. (A) The relationship between glucose levels (blue), meals (grey dotted lines) and sport events (shaded areas with annotations) for participant ID 04. Due to a sensor failure, the glucose data for ID 04 is in two blocks. (B-C) Simulating glucose traces using the full model with random fluctuations creates realistic glucose traces. (B) Examples comparing the CGM data (blue) with the model prediction incorporating circadian dynamics (black) plus meal consumption (orange) for two participants with overdamped and underdamped dynamics, respectively. Same panels as Figure 3E-F, repeated here for easy comparison with panel C. (C) Simulations from the full glucose model that also includes random fluctuations (blue). The timestamps of meals are shown as black dashed lines. (D) Posterior distribution over inferred meal heights for ID 14 and 23, where meal heights are defined as glucose increases relative the baseline glucose levels. The number indicates the number of times the meal was consumed. The boxplots represent the 25th, median (50th) and 75th percentiles of the posterior distribution and the whiskers represent the 5th and 95th percentiles.

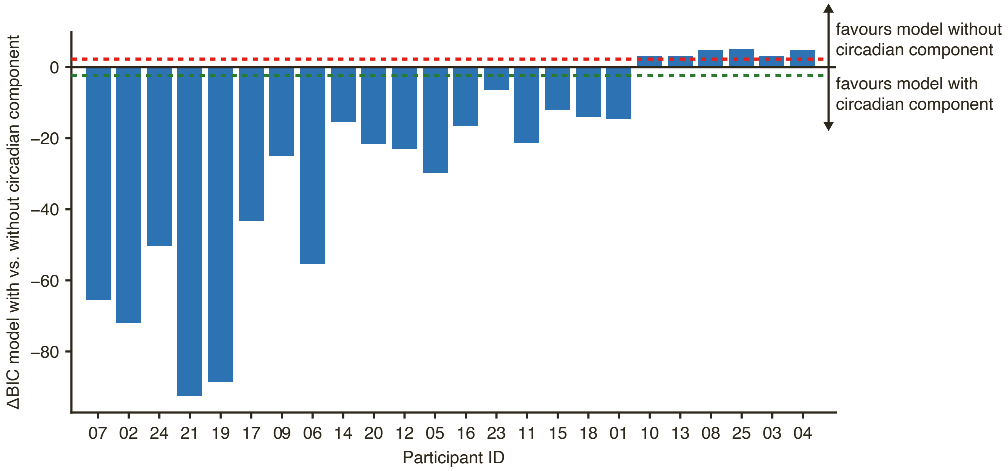

Figure S10: The difference in BIC score between Model 1 with a circadian component and Model 1 without a circadian component. Related to Figure 4. The two models were first optimised to their maximum a posteriori probability (MAP) estimates, then the BIC score was calculated. A negative difference in BIC score favours Model 1 with a circadian component, while a positive difference in BIC score favours the Model 1 without a circadian component. The dotted red line marks the threshold at which Model 1 without the circadian component is preferred, while the dotted green line marks the threshold at which Model 1 with the circadian component is preferred.

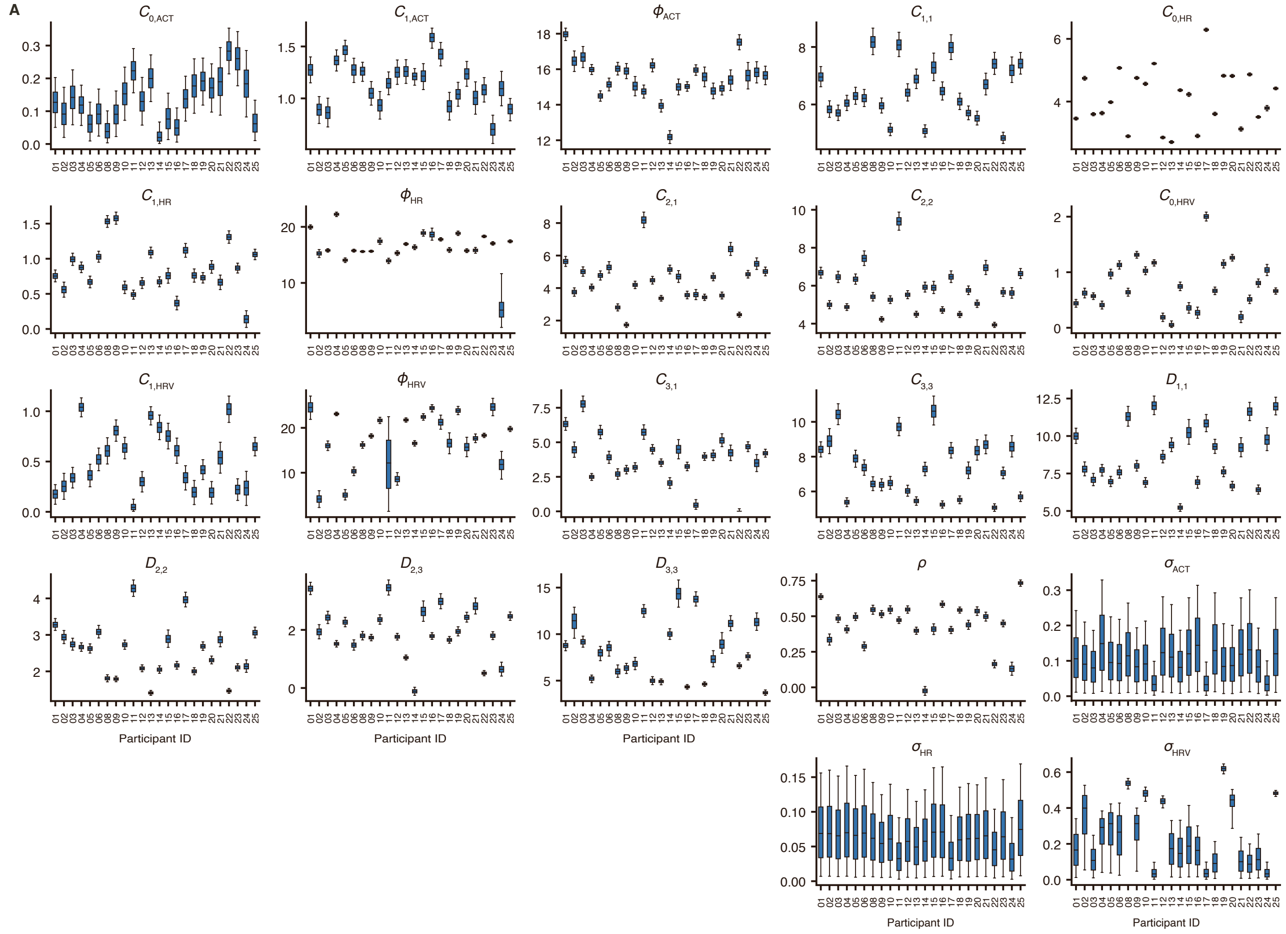

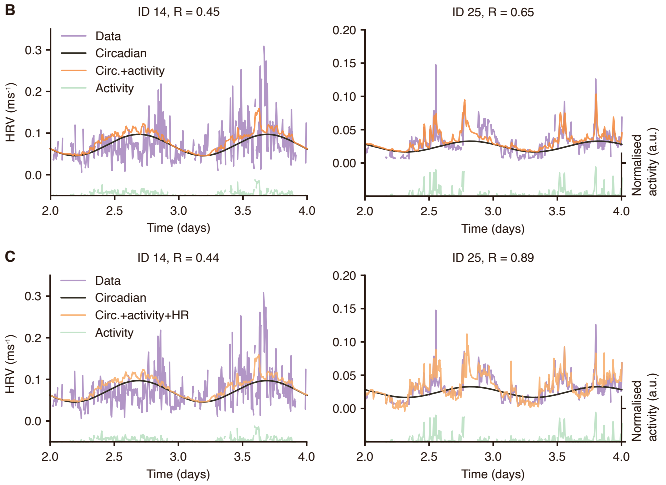

Figure S11: Related to Model 2 and Figure 5. (A) Posterior distributions (shown as boxplots) for all physical and heart activity model parameters (Model 2) across all participants. The boxplots represent the 25th, median (50th) and 75th percentiles of the posterior distribution and the whiskers represent the 5th and 95th percentiles. (B-C) Examples demonstrating the improvement in prediction quality when accounting for HR in predictions of HRV. (B) The prediction of HRV using only circadian time and physical activity. Blue: HRV data; black: baseline circadian rhythm; green: physical activity (shown on normalised scale where 1 represents the maximum value); orange: model prediction using circadian rhythms and physical activity. (C) The prediction of HRV using circadian time, physical activity and HR. Blue: HRV data; black: baseline circadian rhythm; green: physical activity (shown on normalised scale where 1 represents the maximum value); orange: model prediction using circadian rhythms, physical activity and HR.

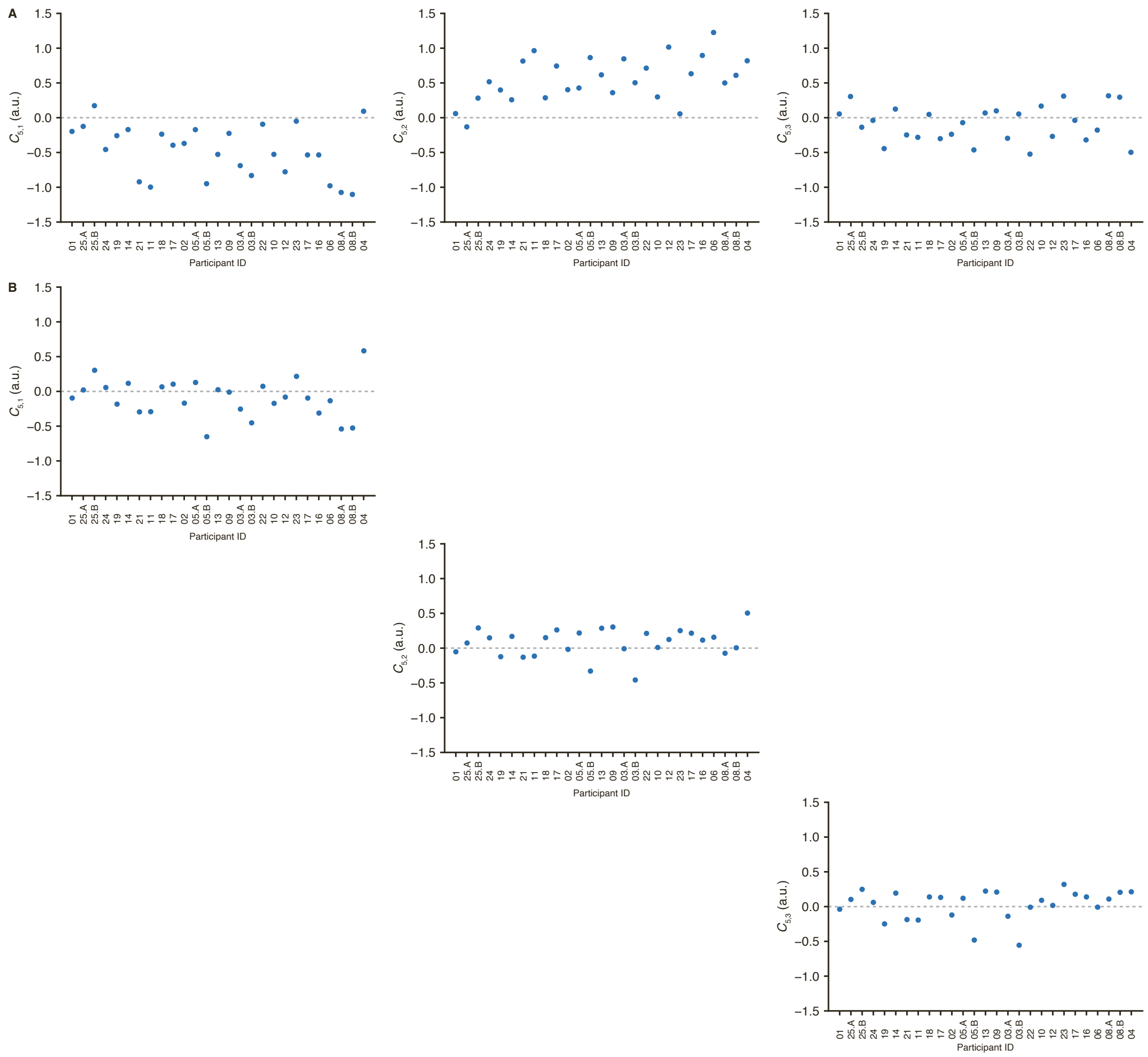

Figure S12: Model parameters when only one input from the physical and heart activity model is allowed. Related to Figure 5. (A) Maximum a posteriori probability (MAP) estimate of coefficients C_{51}, C_{51} and C_{51} with all physical and heart activity inputs. (B) MAP estimate of the coefficients C_{51} when physical activity (via parameter C_{51}) is the only physical and heart activity input into the glucose model. (C) MAP estimate of the coefficients C_{52} when heart rate (via parameter C_{52}) is the only physical and heart activity input into the glucose model. (D) MAP estimate of the coefficients C_{53} when heart rate variability (via parameter C_{53}) is the only physical and heart activity input into the glucose model.

3 Methods S1: details of the Kalman filter model, related to STAR Methods

3.1 Missing data and data quality

For each participant, we collected data for two weeks using the following devices: 1) timestamps of food/drinks and text annotations collected with the smartphone application (app) MyCircadianClock; 2) continuous glucose monitoring (CGM) using the FreeStyle Libre Pro device (Abbott); 3) physical activity, heart rate (HR) and heart rate variability (HRV using RMSSD^{-1}) using the Actiheart device (CamNTech). FreeStyle CGMs were replaced when devices fell off participants within the first week, which occurred 6 times, and data was not used when there was less than 48 hours of valid measurements. For one participant (ID 22), the FreeStyle CGM device failed without falling off, but due to device blinding this was not detected until the end of the experiment, and hence there is no glucose data for this participant. While the Actiheart device is waterproof, participants were permitted to briefly remove the device during showers and baths. At each time point the Actiheart produces an estimated quality of the signal (range 0-1), and we filtered the data based on a threshold of 0.8. After filtering the signal based on the quality, an average of 78.5% of the data was included, although this was specific to each participant (Table S1). There was an Actiheart device failure for one participant (ID 07). The Actiheart data was exported with a time resolution of 5 minutes, while the FreeStyle CGM device records glucose every 15 minutes. To align the Actiheart and FreeStyle CGM time series, we chose the Actiheart measurement that was closest in time to the corresponding CGM measurement. This induces a maximum misalignment of 2.5 minutes, which we consider acceptable given that glucose typically has a response time >1 hour (Figure 3A).

3.2 Modelling wearable time series data

We use a linear Gaussian state space model (otherwise known as a Kalman filter, see Särkkä and Solin [S1] for details) to analyse the time series generated by the wearable devices, and here we will first outline the general parameter inference framework before giving details for the specific models used. To account for both dynamics in underlying physiological variables changes as well as measurement error of the devices, this approach considers underlying physiological variables (referred to as latent state variables) $\boldsymbol{x}(t_k)$ (measured at times t_k) and observed variables $\boldsymbol{y}_k$. The linear Gaussian state space model of the wearable data has the following three components:

3.2.1 Dynamic model

The dynamic model $\boldsymbol{x}(t_k) \sim p\left(\boldsymbol{x}(t_k) \mid \boldsymbol{x}(t_{k-1})\right)$ describes the transition probabilities of the underlying physiological variables. Our starting point is a continuous-time system of stochastic differential equations (SDEs)

$$\mathrm{d}\boldsymbol{x}(t) = W\boldsymbol{x}(t)\mathrm{d}t + \mathrm{d}\boldsymbol{\beta}, \tag{1}$$

where W is a matrix describing the interactions between the variables and $\boldsymbol{\beta}$ is a brownian noise term with covariance matrix Q. The specific forms of W and Q are unique for each model and will be described in the following sections. We then convert this system of continuous-time SDEs into a model where time is discrete.

$$\boldsymbol{x}(t_k) = F_{k-1}\boldsymbol{x}(t_{k-1}) + N(0, \Sigma_{k-1}), \tag{2}$$

where F_k is the state-transition model and Σ_k is the covariance of the process noise. To perform this time discretisation we use the following two formulas, where details on their precise calculation for each model will also be specified below

$$\begin{aligned} F_k &= \exp\left(W\Delta t_k\right), \\ \Sigma_k &= \int_0^{\Delta t_k} \exp\left(W\left(\Delta t_k - \tau\right)\right) Q \exp\left(W\left(\Delta t_k - \tau\right)\right)^\top \mathrm{d}\tau. \end{aligned} \tag{3}$$

3.2.2 Initial distribution

The initial distribution $\boldsymbol{x}(t_0) \sim p\left(\boldsymbol{x}(t_0)\right)$ specifies the distribution of the physiological variables at the initial time $k = 0$. This is specified with $p\left(\boldsymbol{x}\left(t_0\right)\right) = N\left(\boldsymbol{x}\left(t_0\right) \mid \boldsymbol{m}_0, P_0\right)$, and where we use the stationary distribution of the SDE to define the mean and covariance $\boldsymbol{m}_0$ and P_0.

3.2.3 Measurement model

The measurement model $\boldsymbol{y}_k \sim p\left(\boldsymbol{y}_k \mid \boldsymbol{x}(t_k)\right)$ describes the observation process, which again assumes a Gaussian distribution.

$$\boldsymbol{y}(t_k) = H_k\boldsymbol{x}(t_k) + N(\boldsymbol{m}_k, R_k), \tag{4}$$

where H_k is the observation matrix and $\boldsymbol{m}_k$ and R_k represents the mean and covariance of the observation noise, respectively.

3.3 The marginal posterior distribution of parameters

The parameters of the model can be represented with the parameter vector $\boldsymbol{\theta}$, and the goal is to infer the parameters for each participant. Within a Bayesian framework, the parameters of the model can be estimated from the data as follows

$$p\left(\boldsymbol{\theta} \mid \boldsymbol{y}_{1:T}\right) \propto p(\boldsymbol{\theta}) p\left(\boldsymbol{y}_{1:T} \mid \boldsymbol{\theta}\right), \tag{5}$$

where $p(\boldsymbol{\theta})$ is the prior distribution of parameters and $p\left(\boldsymbol{y}_{1:T} \mid \boldsymbol{\theta}\right)$ is the likelihood of observing the temporal data $\boldsymbol{y}_{1:T}$ given the set of parameters $\boldsymbol{\theta}$. Considering the time series sequence of data, the likelihood term for a given set of parameters $\boldsymbol{\theta}$ can be re-expressed as

$$p\left(\boldsymbol{y}_{1:T} \mid \boldsymbol{\theta}\right) = p\left(\boldsymbol{y}_1 \mid \boldsymbol{\theta}\right) \prod_{k=2}^{T} p\left(\boldsymbol{y}_k \mid \boldsymbol{y}_{1:k-1}, \boldsymbol{\theta}\right), \tag{6}$$

and the sequence of distributions $p\left(\boldsymbol{y}_k \mid \boldsymbol{y}_{1:k-1}, \boldsymbol{\theta}\right)$ are calculated within a Kalman filtering framework that we will now describe.

3.4 Kalman Filter

We will now describe the Bayesian filtering approach used to estimate the likelihood term $p\left(\boldsymbol{y}_{1:T} \mid \boldsymbol{\theta}\right)$. Starting with the mean $\boldsymbol{m}_0$ and covariance P_0 of the initial distribution $p\left(\boldsymbol{x}\left(t_0\right)\right) = \mathrm{N}\left(\boldsymbol{x}\left(t_0\right) \mid \boldsymbol{m}_0, P_0\right)$, the Kalman filter recursion operates from the first to last data point $k = 1, 2, ..., T$. At each time point, there is first a prediction step that predicts the current state of the latent variables $\boldsymbol{x}\left(t_k\right)$ based on all previous measurements up to $\boldsymbol{y}_{k-1}$

$$p\left(\boldsymbol{x}\left(t_k\right) \mid \boldsymbol{y}_1, \ldots, \boldsymbol{y}_{k-1}\right) = \mathrm{N}\left(\boldsymbol{x}\left(t_k\right) \mid \boldsymbol{m}_k^-, P_k^-\right), \tag{7}$$

and where the predicted mean and covariance are calculated from

$$\begin{aligned} \boldsymbol{m}_k^- &= F_{k-1}\boldsymbol{m}_{k-1}, \\ P_k^- &= F_{k-1}P_{k-1}F_{k-1}^\top + \Sigma_{k-1}. \end{aligned} \tag{8}$$

The measurement $\boldsymbol{y}_k$ at time t_k is then incorporated to create an updated estimation of the latent state $\boldsymbol{x}\left(t_k\right)$

$$p\left(\boldsymbol{x}\left(t_k\right) \mid \boldsymbol{y}_1, \ldots, \boldsymbol{y}_k\right) = \mathrm{N}\left(\boldsymbol{x}\left(t_k\right) \mid \boldsymbol{m}_k, P_k\right) \tag{9}$$

and where the updated mean and covariance are calculated using the update steps

$$\begin{aligned} \boldsymbol{v}_k &= \boldsymbol{y}_k - H_k\boldsymbol{m}_k^-, \\ S_k &= H_k P_k^- H_k^\top + R_k, \\ K_k &= P_k^- H_k^\top S_k^{-1}, \\ \boldsymbol{m}_k &= \boldsymbol{m}_k^- + K_k\boldsymbol{v}_k, \\ P_k &= P_k^- - K_k S_k K_k^\top. \end{aligned} \tag{10}$$

From these equations the terms necessary for the likelihood (Equation 6) can be calculated as a by-product

$$p\left(\boldsymbol{y}_k \mid \boldsymbol{y}_1, \ldots, \boldsymbol{y}_{k-1}\right) = \mathrm{N}\left(\boldsymbol{y}_k \mid H_k\boldsymbol{m}_k^-, S_k\right). \tag{11}$$

We will now specify the three models used, which allow calculation of the terms in Equation 11 and hence the likelihood using Equation 6. For each of the models we use the "LinearGaussianStateSpaceModel" distribution within Tensorflow Probability. This distribution requires the specification of the mean and covariance of both the dynamics and observation models, and the log likelihood is then calculated using the 'log_prob()' method.

3.5 Model 1: glucose and meals model

We model glucose dynamics with a two-dimensional system of SDEs with two variables: x_{GLUC1} and x_{GLUC2}. The second variable x_{GLUC2} acts as an input into the observation model to produce the measured variable y, which corresponds to the continuous glucose monitor (CGM) data. In matrix form, the model is expressed as follows

$$\begin{aligned} &\mathrm{d}\boldsymbol{x}(t) = W\boldsymbol{x}(t)\mathrm{d}t + \mathrm{d}\boldsymbol{\beta} \\ &\boldsymbol{x}(t) = \begin{bmatrix} x_{\text{GLUC1}}(t) \\ x_{\text{GLUC2}}(t) \end{bmatrix}, W = \begin{bmatrix} -A_{11} & -A_{12} \\ A_{21} & -A_{22} \end{bmatrix}, Q = \begin{bmatrix} 0 & 0 \\ 0 & B_{22} \end{bmatrix}, \end{aligned} \tag{12}$$

where the coefficients A_{ij} are constrained to be positive. Given the coefficients in the W matrix, the positive constraints on the coefficients A_{ij} ensures stability of the matrix (i.e. $-A_{11} - A_{22} < 0$ and $A_{11}A_{22} > -A_{12}A_{21}$). There are two principal reasons for modelling the glucose with two variables: 1) the inclusion of a second variable allows for negative feedback via the negative term $-A_{12}$, as negative feedback can arise due to the action of insulin; 2) when meals are consumed, we model this as impacting the first variable x_{GLUC1}. As the first variable x_{GLUC1} acts to increase levels in the second variable x_{GLUC2} (via the parameter A_{21}), this leads to smooth, continuous increases in glucose after food consumption, which is a feature observed in the data. The covariance of the brownian noise term $\boldsymbol{\beta}$ is given by Q.

3.5.1 State-transition model

The state-transition model F_k can be calculated using a 2x2 matrix exponential

$$\begin{aligned} F_k &= \exp\left(W\Delta t_k\right), \\ &= e^{s\Delta t_k}\left(\left(\cosh(\sqrt{\gamma}\Delta t_k) - s\frac{\sinh(\sqrt{\gamma}\Delta t_k)}{\sqrt{\gamma}}\right)I + \frac{\sinh(\sqrt{\gamma}\Delta t_k)}{\sqrt{\gamma}}W\right), \end{aligned} \tag{13}$$

where

$$\begin{aligned} s &= (-A_{11} - A_{22})/2, \\ \gamma &= (-A_{11} + A_{22})^2/4 - A_{12}A_{21} = s^2 - \det(W). \end{aligned} \tag{14}$$

If $\gamma < 0$ then

$$\cosh \sqrt{\gamma}\Delta t_k = \cos \sqrt{-\gamma}\Delta t_k,$$
$$\frac{\sinh \sqrt{\gamma}\Delta t_k}{\sqrt{\gamma}} = \frac{\sin \sqrt{-\gamma}\Delta t_k}{\sqrt{-\gamma}}.$$

3.5.2 Covariance of the process noise

The covariance of the process noise Σ_k can be calculated from the steady-state covariance P_∞ as follows

$$\Sigma(\Delta t_k) = P_\infty - F_k(\Delta t_k) P_\infty F_k^\top(\Delta t_k), \tag{15}$$

where P_∞ can be calculated symbolically using the following formula

$$W P_\infty + P_\infty W^\top + Q = \mathbf{0}. \tag{16}$$

3.5.3 Incorporating meal events

The smartphone application provides a list of the recorded ingestion event times $\{t_m\}_{m=1}^{M}$ for a total of M meals. We model the effect of meal event m as causing an instantaneous increase of size δ_m to variable x_{GLUC1}. Given the delays from eating to reaching the insterstitial tissue measured by the CGM, we also include a time lag parameter τ for each participant, which is learnt in addition to the other parameters during model fitting. The response function $r_m(t, t_m, \boldsymbol{\theta})$ to a meal is then given by

$$r_m(t, t_m, \boldsymbol{\theta}) = \begin{cases} \Theta(t-\tau-t_m)\frac{A_{21}\,\delta_m\,\mathrm{e}^{s\,(t-\tau-t_m)}\sinh\left(\sqrt{\gamma}\,(t-\tau-t_m)\right)}{\sqrt{\gamma}}, & \gamma > 0 \\ \Theta(t-\tau-t_m)\frac{A_{21}\,\delta_m\,\mathrm{e}^{s\,(t-\tau-t_m)}\sin\left(\sqrt{-\gamma}\,(t-\tau-t_m)\right)}{\sqrt{-\gamma}}, & \gamma < 0 \end{cases}, \tag{17}$$

where the Heaviside function $\Theta(t - \tau - t_m)$ is defined as

$$\Theta(x) = \begin{cases} 1, & x > 0 \\ 0, & x \le 0 \end{cases}. \tag{18}$$

The meal function is then sum of the M individual meals

$$r(t) = \sum_{m=1}^{M} r_m(t, t_m, \boldsymbol{\theta}). \tag{19}$$

To simplify the interpretation of the perturbation parameters δ_m (and to also make the definition of the prior simpler), we reparameterise the parameters δ_m to correspond to the maximum height of the glucose meal response. The time $t_{r_{\max}}$ of the maximum height of the meal response is

$$t_{r_{\max}} = \begin{cases} \tanh^{-1}(-\sqrt{\gamma}/s)/\sqrt{\gamma}, & \gamma > 0 \\ \tan^{-1}(-\sqrt{-\gamma}/s)/\sqrt{-\gamma}, & \gamma < 0 \end{cases}, \tag{20}$$

and the the maximum height of the glucose meal response is then given as

$$\max(r_m(t, t_m, \boldsymbol{\theta})) = \begin{cases} \frac{A_{21}\,\delta_m\,\mathrm{e}^{s\,(t_{r_{\max}})}\,\sinh\left(\sqrt{\gamma}\,(t_{r_{\max}})\right)}{\sqrt{\gamma}}, & \gamma > 0 \\ \frac{A_{21}\,\delta_m\,\mathrm{e}^{s\,(t_{r_{\max}})}\,\sin\left(\sqrt{-\gamma}\,(t_{r_{\max}})\right)}{\sqrt{-\gamma}}, & \gamma < 0 \end{cases} \tag{21}$$

A meal height parameter δ_m^* is then defined as $\delta_m^* = \max(r_m(t, t_m, \boldsymbol{\theta}))$, and the original perturbation parameter δ_m is then found by renormalising.

3.5.4 Circadian dynamics

In addition to the effect of external meal events, we also model underlying circadian rhythms in glucose levels with a sinusoidal function

$$g_{\mathrm{GLUC}}(t) = A_{0,\mathrm{GLUC}} + A_{1,\mathrm{GLUC}}(1 + \cos(\omega t - \phi_{\mathrm{GLUC}}))/2, \tag{22}$$

where $A_{0,\mathrm{GLUC}}$ is the baseline level, $A_{1,\mathrm{GLUC}}$ is the amplitude, ω is the frequency (fixed at $2\pi/24$), and ϕ_{GLUC} is the peak time of the 24-hour function.

3.5.5 Observation matrix

The observation matrix H_k is such that only the second variable is observed

$$H_k = [0, 1]\,. \tag{23}$$

3.5.6 Observation model

Finally, the observation model has the following form

$$\begin{aligned} \boldsymbol{y}_k &= H_k \boldsymbol{x}(t_k) + N(\boldsymbol{m}_k, R_k) \\ \boldsymbol{m}_k &= r(t) + g_{\mathrm{GLUC}}(t), R_k = \sigma^2_{\mathrm{GLUC}}. \end{aligned} \tag{24}$$

3.5.7 Parameter description and priors

Parameter	Description	Prior
$A_{0,\text{GLUC}}$	Circadian baseline	$A_{0,\text{GLUC}} \sim$ half-normal(5)
$A_{1,\text{GLUC}}$	Circadian amplitude	$A_{1,\text{GLUC}} \sim$ half-normal(1)
ϕ_{GLUC}	Circadian phase	$\phi_{\text{GLUC}} \sim \text{U}(0, 24)$
A_{11}	x_{GLUC1} degradation rate	$\log(A_{11}) \sim \text{N}(0, 1)$
A_{12}	Suppression of x_{GLUC1} by x_{GLUC2}	$\log(A_{12}) \sim \text{N}(0, 1)$
A_{21}	Activation of x_{GLUC2} by x_{GLUC1}	$\log(A_{21}) \sim \text{N}(0, 1)$
A_{22}	x_{GLUC2} degradation rate	$\log(A_{22}) \sim \text{N}(0, 1)$
τ	Delay in meal response	$\tau \sim$ half-normal(0.5)
B_{22}	Glucose diffusion noise	$B_{22} \sim$ half-normal(0.5)
δ_m^*	Meal-induced glucose increase	$\delta_m^* \sim$ half-normal(5)

Parameters Model 1 : glucose and meals model.

3.6 Model 2: physical and heart activity model

We model physical and heart activity dynamics with a three-dimensional system of SDEs, where the first variable x_{ACT} represents physical activity, the second variable x_{HR} represents heart rate and the third variable x_{HRV} represents heart rate variability, where we use the inverse of the root mean square of successive differences between normal heartbeats (RMSSD^{-1}). We normalise all three variables by their respective standard deviations before inferring parameters. In matrix form, the model is expressed as follows

$$
\begin{aligned}
&\mathrm{d}\boldsymbol{x}(t) = W\boldsymbol{x}(t)\mathrm{d}t + \mathrm{d}\boldsymbol{\beta}, \\
&\boldsymbol{x}(t) = \begin{bmatrix} x_{\text{ACT}}(t) \\ x_{\text{HR}}(t) \\ x_{\text{HRV}}(t) \end{bmatrix} W = \begin{bmatrix} -C_{11} & 0 & 0 \\ C_{21} & -C_{22} & 0 \\ C_{31} & 0 & -C_{33} \end{bmatrix} Q = \begin{pmatrix} D_{11} & 0 & 0 \\ 0 & D_{22} & \rho\sqrt{D_{22}D_{33}} \\ 0 & \rho\sqrt{D_{22}D_{33}} & D_{33} \end{pmatrix},
\end{aligned} \tag{25}
$$

where the coefficients C_{ij} are constrained to be positive. The covariance of the brownian noise term $\boldsymbol{\beta}$ is given by Q. Dynamic changes and fluctuations in HR and HRV might be correlated, where this correlation is mediated by e.g. the autonomic nervous system. In the model, the correlation in the fluctuations between HR and HRV is quantified with the correlation parameter $\rho \in (-1, 1)$.

3.6.1 State-transition model

The state-transition model F_k can be calculated using a 3x3 matrix exponential

$$
\begin{aligned}
F_k &= \exp\left(W\Delta t_k\right), \\
&= \begin{pmatrix} \mathrm{e}^{-C_{11}\,\Delta t_k} & 0 & 0 \\ -\frac{C_{21}\,\mathrm{e}^{-C_{11}\,\Delta t_k} - C_{21}\,\mathrm{e}^{-C_{22}\,\Delta t_k}}{C_{11}-C_{22}} & \mathrm{e}^{-C_{22}\,\Delta t_k} & 0 \\ -\frac{C_{31}\,\mathrm{e}^{-C_{11}\,\Delta t_k} - C_{31}\,\mathrm{e}^{-C_{33}\,\Delta t_k}}{C_{11}-C_{33}} & 0 & \mathrm{e}^{-C_{33}\,\Delta t_k} \end{pmatrix}.
\end{aligned} \tag{26}
$$

3.6.2 Covariance of the process noise

Similarly to the glucose model, the covariance of the process noise is calculated symbolically using the following formulae

$$
\Sigma(\Delta t_k) = P_\infty - F_k(\Delta t_k) P_\infty F_k^\top(\Delta t_k), \tag{27}
$$

$$
W P_\infty + P_\infty W^\top + Q = \mathbf{0}. \tag{28}
$$

3.6.3 Circadian dynamics

We model the underlying circadian rhythms in activity levels with a sinusoidal function

$$
g_{\mathrm{ACT}}(t) = C_{0,\mathrm{ACT}} + C_{1,\mathrm{ACT}}(1 + \cos(\omega t - \phi_{\mathrm{ACT}}))/2, \tag{29}
$$

where C_0 is the baseline level, C_1 is the amplitude, ω is the frequency (fixed at $2\pi/24$), and ϕ_{ACT} is the peak time of the function. Given that physical activity acts as an input into HR, the circadian function for HR receives an input from physical activity. We therefore model the circadian rhythms for HR using two functions: a term that integrates input from physical activity $g^1_{\mathrm{HR}}(t)$ and an independent function $g^2_{\mathrm{HR}}(t)$. The expression for the term $g^1_{\mathrm{HR}}(t)$ that integrates input from physical activity is then given by

$$
\begin{aligned}
\frac{d}{dt} g^1_{\mathrm{HR}}(t) &= C_{11} g_{\mathrm{ACT}}(t) - C_{22} g^1_{\mathrm{HR}}(t), \\
g^1_{\mathrm{HR}}(t) &= \frac{C_0\,C_{21}}{C_{22}} + \frac{C_1\,C_{21}}{2\,C_{22}} - \frac{C_1\,C_{21}\,\omega\,\sin\left(\varphi_{\mathrm{ACT}} - \omega\,t\right)}{2\,\left({C_{22}}^2 + \omega^2\right)} + \frac{C_1\,C_{21}\,C_{22}\,\cos\left(\varphi_{\mathrm{ACT}} - \omega\,t\right)}{2\,\left({C_{22}}^2 + \omega^2\right)}.
\end{aligned} \tag{30}
$$

We then model the additional circadian component $g^2_{\mathrm{HR}}(t)$ with a separate function

$$
g^2_{\mathrm{HR}}(t) = C_{0,\mathrm{HR}} + C_{1,\mathrm{HR}}(1 + \cos(\omega t - \phi_{\mathrm{HR}}))/2. \tag{31}
$$

The total circadian function is then a sum of these two components

$$
g_{\mathrm{HR}}(t) = g^1_{\mathrm{HR}}(t) + g^2_{\mathrm{HR}}(t). \tag{32}
$$

The same framework is then used for the HRV signal, with a term that integrates input from physical activity $g^1_{\text{HRV}}(t)$ and an independent function $g^2_{\text{HRV}}(t)$. The expression for the term $g^1_{\text{HRV}}(t)$ that integrates input from physical activity is then given by

$$\begin{aligned} \frac{d}{dt} g^1_{\text{HRV}}(t) &= C_{11} g_{\text{ACT}}(t) - C_{22} g^1_{\text{HRV}}(t) \\ g^1_{\text{HRV}}(t) &= \frac{C_0\, C_{31}}{C_{33}} + \frac{C_1\, C_{31}}{2\, C_{33}} - \frac{C_1\, C_{31}\, \omega\, \sin\left(\varphi_{\text{ACT}} - \omega\, t\right)}{2\, \left({C_{33}}^2 + \omega^2\right)} + \frac{C_1\, C_{31}\, C_{33}\, \cos\left(\varphi_{\text{ACT}} - \omega\, t\right)}{2\, \left({C_{33}}^2 + \omega^2\right)}. \end{aligned} \tag{33}$$

We then model additional HRV circadian component $g^2_{\text{HRV}}(t)$ with a separate function

$$g^2_{\text{HR}}(t) = C_{0,\text{HRV}} + C_{1,\text{HRV}}(1 + \cos(\omega t - \phi_{\text{HRV}}))/2. \tag{34}$$

The total circadian function is then a sum of these two components

$$g_{\text{HRV}}(t) = g^1_{\text{HRV}}(t) + g^2_{\text{HRV}}(t). \tag{35}$$

3.6.4 Observation matrix

The observation matrix H_k is such that all three variables are observed

$$H_k = \begin{pmatrix} 1 & 0 & 0 \\ 0 & 1 & 0 \\ 0 & 0 & 1 \end{pmatrix}. \tag{36}$$

3.6.5 Observation model

Finally, the observation model has the following form

$$\begin{aligned} \boldsymbol{y}(t_k) &= H_k \boldsymbol{x}(t_k) + N(\boldsymbol{m}_k, R_k), \\ \boldsymbol{m}_k &= \begin{bmatrix} g_{\text{ACT}}(t) \\ g_{\text{HR}}(t) \\ g_{\text{HRV}}(t) \end{bmatrix}, R_k = \begin{bmatrix} \sigma^2_{\text{ACT}} & 0 & 0 \\ 0 & \sigma^2_{\text{HR}} & 0 \\ 0 & 0 & \sigma^2_{\text{HRV}} \end{bmatrix}. \end{aligned} \tag{37}$$

3.6.6 Parameter description and priors

Parameter	Description	Prior
$C_{0,\mathrm{ACT}}, C_{0,\mathrm{HR}}, C_{0,\mathrm{HRV}}$	Circadian baseline (activity, HR, HRV)	$C_{0,\mathrm{ACT}}, C_{0,\mathrm{HR}}, C_{0,\mathrm{HRV}} \sim$ half-normal(1)
$C_{1,\mathrm{ACT}}, C_{1,\mathrm{HR}}, C_{1,\mathrm{HRV}}$	Circadian amplitude (activity, HR, HRV)	$C_{1,\mathrm{ACT}}, C_{1,\mathrm{HR}}, C_{1,\mathrm{HRV}} \sim$ half-normal(1)
$\phi_{\mathrm{ACT}}, \phi_{\mathrm{HR}}, \phi_{\mathrm{HRV}}$	Circadian phase (activity, HR, HRV)	$\phi_{\mathrm{ACT}}, \phi_{\mathrm{HR}}, \phi_{\mathrm{HRV}} \sim \mathrm{U}(0, 24)$
C_{11}, C_{22}, C_{33}	Relaxation rate (activity, HR, HRV)	$C_{11}^{-1}, C_{22}^{-1}, C_{33}^{-1} \sim$ half-normal(0.01)
C_{21}	Activation of x_{HR} by x_{ACT}	$\log(C_{21}) \sim \mathrm{N}(0, 5)$
C_{31}	Activation of x_{HRV} by x_{ACT}	$\log(C_{31}) \sim \mathrm{N}(0, 5)$
ρ	Correlation in diffusion between x_{HR} and x_{HRV}	$\rho \sim \mathrm{U}(-1, 1)$
D_{11}, D_{22}, D_{33}	Diffusion noise (activity, HR, HRV)	$D_{11}, D_{22}, D_{33} \sim$ half-normal(5)
$\sigma_{\mathrm{ACT}}, \sigma_{\mathrm{HR}}, \sigma_{\mathrm{HRV}}$	Measurement noise (activity, HR, HRV)	$\sigma_{\mathrm{ACT}}, \sigma_{\mathrm{HR}}, \sigma_{\mathrm{HRV}} \sim$ half-normal(1)

Parameters Model 2 : physical and heart activity model.

3.7 Model 3: combined model

The final model connects the physical and heart activity signals with glucose dynamics by connecting Model 1 (the glucose and meals model) with Model 2 (physical and heart activity). Three new parameters C_{51}, C_{52} and C_{53} are introduced that describe the effect of physical activity, HR and HRV on glucose levels (x_{GLUC2}), respectively. These three parameters are left unconstrained and can take either positive, negative or zero values.

$$
\begin{aligned}
\mathrm{d}\boldsymbol{x}(t) &= W\boldsymbol{x}(t)\mathrm{d}t + \mathrm{d}\boldsymbol{\beta} \\
\boldsymbol{x}(t) &= \begin{bmatrix} x_{\mathrm{ACT}}(t) \\ x_{\mathrm{HR}}(t) \\ x_{\mathrm{HRV}}(t) \\ x_{\mathrm{GLUC1}}(t) \\ x_{\mathrm{GLUC2}}(t) \end{bmatrix} W = \begin{bmatrix} -C_{11} & 0 & 0 & 0 & 0 \\ C_{21} & -C_{22} & 0 & 0 & 0 \\ C_{31} & 0 & -C_{33} & 0 & 0 \\ 0 & 0 & 0 & -A_{11} & -A_{12} \\ C_{51} & C_{52} & C_{53} & A_{21} & -A_{22} \end{bmatrix} \\
Q &= \begin{bmatrix} D_{11} & 0 & 0 & 0 & 0 \\ 0 & D_{22} & \rho\sqrt{D_{22}D_{33}} & 0 & 0 \\ 0 & \rho\sqrt{D_{22}D_{33}} & D_{33} & 0 & 0 \\ 0 & 0 & 0 & 0 & 0 \\ 0 & 0 & 0 & 0 & B_{22} \end{bmatrix}.
\end{aligned} \tag{38}
$$

To simplify the model inference problem, the parameters from Model 2 describing the physical and heart activity model in isolation were locked to their posterior mean values. The covariance of the brownian noise

term $\boldsymbol{\beta}$ is given by Q.

3.7.1 State-transition model

Symbolic calculation of the matrix exponential required for the state-transition model becomes too cumbersome for Model 3, and we therefore use a Taylor series to approximate it [S2].

$$\exp\left(W\Delta t_k\right) = \sum_{j=0}^{l} (W\Delta t_k)^j / j!, \tag{39}$$

along with the scaling property

$$e^{(W\Delta t_k)} = \left(e^{(W\Delta t_k)/m}\right)^m. \tag{40}$$

We use $l = 5$ and $m = 2$.

3.7.2 Covariance of the process noise

For the covariance of the process noise, we use the Matrix Fraction Decomposition approach [S3-S4] to solve

$$\frac{\mathrm{d}\Sigma(t)}{\mathrm{d}t} = W\Sigma(t) + \Sigma(t)W^\top + Q, \quad \Sigma(0) = \mathbf{0} \tag{41}$$

If we define matrices $C_\Sigma(\Delta t)$ and $D_\Sigma(\Delta t)$ such that $\Sigma(\Delta t) = C_\Sigma(\Delta t) D_\Sigma^{-1}(\Delta t)$ then we can now use matrix fractions to solve

$$\begin{aligned} \begin{pmatrix} C_\Sigma(\Delta t) \\ D_\Sigma(\Delta t) \end{pmatrix} &= \exp\left[\begin{pmatrix} W & Q \\ \mathbf{0} & -W^\top \end{pmatrix} \Delta t\right] \begin{pmatrix} \mathbf{0} \\ \boldsymbol{I} \end{pmatrix} \\ &= \begin{pmatrix} \exp(W\Delta t) & \Sigma(\Delta t)\exp(W\Delta t)^{-\top} \\ \mathbf{0} & \exp(W\Delta t)^{-\top} \end{pmatrix} \begin{pmatrix} \mathbf{0} \\ \boldsymbol{I} \end{pmatrix} \end{aligned} \tag{42}$$

As $D_\Sigma^{-1}(\Delta t) = \exp(W\Delta t)^\top$, there is no need for a matrix inversion.

3.7.3 Observation matrix

The observation matrix H_k is such that physical activity ($x_{\mathrm{ACT}}(t)$), HR ($x_{\mathrm{HR}}(t)$) and HRV ($x_{\mathrm{HRV}}(t)$) are observed along with the glucose levels ($x_{\mathrm{GLUC2}}(t)$).

$$H_k = \begin{pmatrix} 1 & 0 & 0 & 0 & 0 \\ 0 & 1 & 0 & 0 & 0 \\ 0 & 0 & 1 & 0 & 0 \\ 0 & 0 & 0 & 0 & 1 \end{pmatrix} \tag{43}$$

3.7.4 Observation model

Finally, the observation model has the following form

$$\boldsymbol{y}_k = H_k \boldsymbol{x}(t_k) + N(\boldsymbol{m}_k, R_k)$$
$$\boldsymbol{m}_k = \begin{bmatrix} g_{\mathrm{ACT}}(t) \\ g_{\mathrm{HR}}(t) \\ g_{\mathrm{HRV}}(t) \\ g_{\mathrm{GLUC}}(t) \end{bmatrix}, R_k = \begin{bmatrix} \sigma^2_{\mathrm{ACT}} & 0 & 0 & 0 \\ 0 & \sigma^2_{\mathrm{HR}} & 0 & 0 \\ 0 & 0 & \sigma^2_{\mathrm{HRV}} & 0 \\ 0 & 0 & 0 & \sigma^2_{\mathrm{gluc}} \end{bmatrix} \tag{44}$$

3.7.5 Parameter description and priors

Parameters for the physical and heart activity model (Model 2) are fixed at their posterior mean values, and the parameters for the glucose model (Model 1) are relearnt with the same priors. The priors for the 3 new parameters (C_{51}, C_{52}, C_{53}) in Model 3 are then as follows

Parameter	Description	Prior
C_{51}	Activation of $x_{\mathrm{GLUC2}}(t)$ by activity	$C_{51} \sim \mathrm{N}(0,1)$
C_{52}	Activation of $x_{\mathrm{GLUC2}}(t)$ by HR	$C_{52} \sim \mathrm{N}(0,1)$
C_{53}	Activation of $x_{\mathrm{GLUC2}}(t)$ by HRV	$C_{53} \sim \mathrm{N}(0,1)$

Parameters Model 3 : combined model.

3.8 Posterior parameter sampling with MCMC

The parameter posterior distribution was sampled using Hamiltonian Markov Chain Monte Carlo (HMC), which uses the gradients of the posterior to improve the efficiency of the sampling. To initialise the sampler, we found the maximum a posteriori probability (MAP) parameter estimate using the BFGS optimiser 'bfgs_minimize' within TensorFlow Probability. We then used the 'HamiltonianMonteCarlo' function with TensorFlow Probability with 5 leapfrog steps, and we scaled the step size of each variable to approximately match the standard deviation of the posterior distribution. To achieve this, we sampled posterior parameters using two steps. Firstly, we sampled 10000 parameters (with a burn-in of 10,000 samples) using the 'SimpleStepSizeAdaptation' kernel to select the global step size, which adapts the global step size to achieve a target acceptance probability of 0.75 [S5]. We then scaled the step size of each variable according to the standard deviation of this initial posterior distribution. Next, we resampled model parameters from the posterior distribution using 4 different chains with 10,000 samples each (with a burn-in of 10,000 samples), again using 'SimpleStepSizeAdaptation' kernel to globally rescale the step size. The 'SimpleStepSizeAdaptation' kernel was only applied to first 80% of the burn-in samples.

4 References

S1 Simo Särkkä and Arno Solin (2019). Applied Stochastic Differential Equations. Cambridge University Press.

S2 Moler C, Van Loan C (2003). Nineteen dubious ways to compute the exponential of a matrix, twenty-five years later. SIAM Rev.,45: 3-49.

S3 Simo Särkkä (2006) Recursive Bayesian Inference on Stochastic Differential Equations. Doctoral dissertation, Helsinki University of Technology, Espoo, Finland.

S4 Axelsson, P. and Gustafsson, F. (2015). Discrete-time solutions to the continuous-time differential Lyapunov equation with applications to Kalman filtering. IEEE Transactions on Automatic Control, 60(3), 632-643.

S5 Andrieu, Christophe, Thoms, Johannes. A tutorial on adaptive MCMC. Statistics and Computing, 2008. https://people.eecs.berkeley.edu/~jordan/sail/readings/andrieu-thoms.pdf
